# Exploring baryon semileptonic decays through polarization and entanglement

**DOI:** 10.1038/s41586-026-10818-8

**Published:** 2026-09-02

**Authors:** The BESIII Collaboration, M. Ablikim, M. N. Achasov, P. Adlarson, X. C. Ai, R. Aliberti, A. Amoroso, Q. An, Y. Bai, O. Bakina, Y. Ban, H.-R. Bao, V. Batozskaya, K. Begzsuren, N. Berger, M. Berlowski, M. Bertani, D. Bettoni, F. Bianchi, E. Bianco, A. Bortone, I. Boyko, R. A. Briere, A. Brueggemann, H. Cai, M. H. Cai, X. Cai, A. Calcaterra, G. F. Cao, N. Cao, S. A. Cetin, X. Y. Chai, J. F. Chang, G. R. Che, Y. Z. Che, C. H. Chen, Chao Chen, G. Chen, H. S. Chen, H. Y. Chen, M. L. Chen, S. J. Chen, S. L. Chen, S. M. Chen, T. Chen, X. R. Chen, X. T. Chen, X. Y. Chen, Y. B. Chen, Y. Q. Chen, Y. Q. Chen, Z. Chen, Z. J. Chen, Z. K. Chen, S. K. Choi, X. Chu, G. Cibinetto, F. Cossio, J. Cottee-Meldrum, J. J. Cui, H. L. Dai, J. P. Dai, A. Dbeyssi, R. E. de Boer, D. Dedovich, C. Q. Deng, Z. Y. Deng, A. Denig, I. Denysenko, M. Destefanis, F. De Mori, B. Ding, X. X. Ding, Y. Ding, Y. Ding, Y. X. Ding, J. Dong, L. Y. Dong, M. Y. Dong, X. Dong, M. C. Du, S. X. Du, S. X. Du, Y. Y. Duan, Z. H. Duan, P. Egorov, G. F. Fan, J. J. Fan, Y. H. Fan, J. Fang, J. Fang, S. S. Fang, W. X. Fang, Y. Q. Fang, L. Fava, F. Feldbauer, G. Felici, C. Q. Feng, J. H. Feng, L. Feng, Q. X. Feng, Y. T. Feng, M. Fritsch, C. D. Fu, J. L. Fu, Y. W. Fu, H. Gao, X. B. Gao, Y. Gao, Y. N. Gao, Y. N. Gao, Y. Y. Gao, S. Garbolino, I. Garzia, L. Ge, P. T. Ge, Z. W. Ge, C. Geng, E. M. Gersabeck, A. Gilman, K. Goetzen, J. D. Gong, L. Gong, W. X. Gong, W. Gradl, S. Gramigna, M. Greco, M. H. Gu, Y. T. Gu, C. Y. Guan, A. Q. Guo, L. B. Guo, M. J. Guo, R. P. Guo, Y. P. Guo, A. Guskov, J. Gutierrez, K. L. Han, T. T. Han, F. Hanisch, K. D. Hao, X. Q. Hao, F. A. Harris, K. K. He, K. L. He, F. H. Heinsius, C. H. Heinz, Y. K. Heng, C. Herold, P. C. Hong, G. Y. Hou, X. T. Hou, Y. R. Hou, Z. L. Hou, H. M. Hu, J. F. Hu, Q. P. Hu, S. L. Hu, T. Hu, Y. Hu, Z. M. Hu, G. S. Huang, K. X. Huang, L. Q. Huang, P. Huang, X. T. Huang, Y. P. Huang, Y. S. Huang, T. Hussain, N. Hüsken, N. in der Wiesche, J. Jackson, Q. Ji, Q. P. Ji, W. Ji, X. B. Ji, X. L. Ji, Y. Y. Ji, Z. K. Jia, D. Jiang, H. B. Jiang, P. C. Jiang, S. J. Jiang, T. J. Jiang, X. S. Jiang, Y. Jiang, J. B. Jiao, J. K. Jiao, Z. Jiao, S. Jin, Y. Jin, M. Q. Jing, X. M. Jing, T. Johansson, S. Kabana, N. Kalantar-Nayestanaki, X. L. Kang, X. S. Kang, M. Kavatsyuk, B. C. Ke, V. Khachatryan, A. Khoukaz, R. Kiuchi, O. B. Kolcu, B. Kopf, M. Kuessner, X. Kui, N. Kumar, A. Kupsc, W. Kühn, Q. Lan, W. N. Lan, T. T. Lei, M. Lellmann, T. Lenz, C. Li, C. Li, C. H. Li, C. K. Li, D. M. Li, F. Li, G. Li, H. B. Li, H. J. Li, H. N. Li, Hui Li, J. R. Li, J. S. Li, K. Li, K. L. Li, K. L. Li, L. J. Li, Lei Li, M. H. Li, M. R. Li, P. L. Li, P. R. Li, Q. M. Li, Q. X. Li, R. Li, S. X. Li, T. Li, T. Y. Li, W. D. Li, W. G. Li, X. Li, X. H. Li, X. L. Li, X. Y. Li, X. Z. Li, Y. Li, Y. G. Li, Y. P. Li, Z. J. Li, Z. Y. Li, C. Liang, H. Liang, Y. F. Liang, Y. T. Liang, G. R. Liao, L. B. Liao, M. H. Liao, Y. P. Liao, J. Libby, A. Limphirat, C. C. Lin, D. X. Lin, L. Q. Lin, T. Lin, B. J. Liu, B. X. Liu, C. Liu, C. X. Liu, F. Liu, F. H. Liu, Feng Liu, G. M. Liu, H. Liu, H. B. Liu, H. H. Liu, H. M. Liu, Huihui Liu, J. B. Liu, J. J. Liu, K. Liu, K. Liu, K. Y. Liu, Ke Liu, L. C. Liu, Lu Liu, M. H. Liu, M. H. Liu, P. L. Liu, Q. Liu, S. B. Liu, T. Liu, W. K. Liu, W. M. Liu, W. T. Liu, X. Liu, X. Liu, X. K. Liu, X. L. Liu, X. Y. Liu, Y. Liu, Y. Liu, Y. B. Liu, Z. A. Liu, Z. D. Liu, Z. Q. Liu, X. C. Lou, F. X. Lu, H. J. Lu, J. G. Lu, X. L. Lu, Y. Lu, Y. H. Lu, Y. P. Lu, Z. H. Lu, C. L. Luo, J. R. Luo, J. S. Luo, M. X. Luo, T. Luo, X. L. Luo, Z. Y. Lv, X. R. Lyu, Y. F. Lyu, Y. H. Lyu, F. C. Ma, H. L. Ma, Heng Ma, J. L. Ma, L. L. Ma, L. R. Ma, Q. M. Ma, R. Q. Ma, R. Y. Ma, T. Ma, X. T. Ma, X. Y. Ma, Y. M. Ma, F. E. Maas, I. MacKay, M. Maggiora, S. Malde, Q. A. Malik, H. X. Mao, Y. J. Mao, Z. P. Mao, S. Marcello, A. Marshall, F. M. Melendi, Y. H. Meng, Z. X. Meng, G. Mezzadri, H. Miao, T. J. Min, R. E. Mitchell, X. H. Mo, B. Moses, N. YU. Muchnoi, J. Muskalla, Y. Nefedov, F. Nerling, L. S. Nie, I. B. Nikolaev, Z. Ning, S. Nisar, Q. L. Niu, W. D. Niu, C. Normand, S. L. Olsen, Q. Ouyang, S. Pacetti, X. Pan, Y. Pan, A. Pathak, Y. P. Pei, M. Pelizaeus, H. P. Peng, X. J. Peng, Y. Y. Peng, K. Peters, K. Petridis, J. L. Ping, R. G. Ping, S. Plura, V. Prasad, F. Z. Qi, H. R. Qi, M. Qi, S. Qian, W. B. Qian, C. F. Qiao, J. H. Qiao, J. J. Qin, J. L. Qin, L. Q. Qin, L. Y. Qin, P. B. Qin, X. P. Qin, X. S. Qin, Z. H. Qin, J. F. Qiu, Z. H. Qu, J. Rademacker, C. F. Redmer, A. Rivetti, M. Rolo, G. Rong, S. S. Rong, F. Rosini, Ch. Rosner, M. Q. Ruan, N. Salone, A. Sarantsev, Y. Schelhaas, K. Schoenning, M. Scodeggio, K. Y. Shan, W. Shan, X. Y. Shan, Z. J. Shang, J. F. Shangguan, L. G. Shao, M. Shao, C. P. Shen, H. F. Shen, W. H. Shen, X. Y. Shen, B. A. Shi, H. Shi, J. L. Shi, J. Y. Shi, S. Y. Shi, X. Shi, H. L. Song, J. J. Song, T. Z. Song, W. M. Song, Y. J. Song, Y. X. Song, Zirong Song, S. Sosio, S. Spataro, S. Stansilaus, F. Stieler, S. S. Su, Y. J. Su, G. B. Sun, G. X. Sun, H. Sun, H. K. Sun, J. F. Sun, K. Sun, L. Sun, S. S. Sun, T. Sun, Y. C. Sun, Y. H. Sun, Y. J. Sun, Y. Z. Sun, Z. Q. Sun, Z. T. Sun, C. J. Tang, G. Y. Tang, J. Tang, J. J. Tang, L. F. Tang, Y. A. Tang, L. Y. Tao, M. Tat, J. X. Teng, J. Y. Tian, W. H. Tian, Y. Tian, Z. F. Tian, I. Uman, B. Wang, B. Wang, Bo Wang, C. Wang, C. Wang, Cong Wang, D. Y. Wang, H. J. Wang, J. J. Wang, K. Wang, L. L. Wang, L. W. Wang, M. Wang, M. Wang, N. Y. Wang, Shun Wang, T. Wang, T. J. Wang, W. Wang, W. Wang, W. P. Wang, X. Wang, X. F. Wang, X. J. Wang, X. L. Wang, X. N. Wang, Y. Wang, Y. D. Wang, Y. F. Wang, Y. H. Wang, Y. J. Wang, Y. L. Wang, Y. N. Wang, Y. Q. Wang, Yaqian Wang, Yuan Wang, Z. Wang, Z. L. Wang, Z. L. Wang, Z. Q. Wang, Z. Y. Wang, D. H. Wei, H. R. Wei, F. Weidner, S. P. Wen, Y. R. Wen, U. Wiedner, G. Wilkinson, M. Wolke, C. Wu, J. F. Wu, L. H. Wu, L. J. Wu, L. J. Wu, Lianjie Wu, S. G. Wu, S. M. Wu, X. Wu, X. H. Wu, Y. J. Wu, Z. Wu, L. Xia, X. M. Xian, B. H. Xiang, D. Xiao, G. Y. Xiao, H. Xiao, Y. L. Xiao, Z. J. Xiao, C. Xie, K. J. Xie, X. H. Xie, Y. Xie, Y. G. Xie, Y. H. Xie, Z. P. Xie, T. Y. Xing, C. F. Xu, C. J. Xu, G. F. Xu, H. Y. Xu, H. Y. Xu, M. Xu, Q. J. Xu, Q. N. Xu, T. D. Xu, W. Xu, W. L. Xu, X. P. Xu, Y. Xu, Y. Xu, Y. C. Xu, Z. S. Xu, F. Yan, H. Y. Yan, L. Yan, W. B. Yan, W. C. Yan, W. H. Yan, W. P. Yan, X. Q. Yan, H. J. Yang, H. L. Yang, H. X. Yang, J. H. Yang, R. J. Yang, T. Yang, Y. Yang, Y. F. Yang, Y. H. Yang, Y. Q. Yang, Y. X. Yang, Y. Z. Yang, M. Ye, M. H. Ye, Z. J. Ye, Junhao Yin, Z. Y. You, B. X. Yu, C. X. Yu, G. Yu, J. S. Yu, L. Q. Yu, M. C. Yu, T. Yu, X. D. Yu, Y. C. Yu, C. Z. Yuan, H. Yuan, J. Yuan, J. Yuan, L. Yuan, S. C. Yuan, S. H. Yuan, X. Q. Yuan, Y. Yuan, Z. Y. Yuan, C. X. Yue, Ying Yue, A. A. Zafar, S. H. Zeng, X. Zeng, Y. Zeng, Y. J. Zeng, Y. J. Zeng, X. Y. Zhai, Y. H. Zhan, Zhang, A. Q. Zhang, B. L. Zhang, B. X. Zhang, D. H. Zhang, G. Y. Zhang, G. Y. Zhang, H. Zhang, H. Zhang, H. C. Zhang, H. H. Zhang, H. Q. Zhang, H. R. Zhang, H. Y. Zhang, J. Zhang, J. Zhang, J. J. Zhang, J. L. Zhang, J. Q. Zhang, J. S. Zhang, J. W. Zhang, J. X. Zhang, J. Y. Zhang, J. Z. Zhang, Jianyu Zhang, L. M. Zhang, Lei Zhang, N. Zhang, P. Zhang, Q. Zhang, Q. Y. Zhang, R. Y. Zhang, S. H. Zhang, Shulei Zhang, X. M. Zhang, X. Y. Zhang, X. Y. Zhang, Y. Zhang, Y. Zhang, Y. T. Zhang, Y. H. Zhang, Y. M. Zhang, Y. P. Zhang, Z. D. Zhang, Z. H. Zhang, Z. L. Zhang, Z. L. Zhang, Z. X. Zhang, Z. Y. Zhang, Z. Y. Zhang, Z. Z. Zhang, ZH. ZH. Zhang, G. Zhao, J. Y. Zhao, J. Z. Zhao, L. Zhao, L. Zhao, M. G. Zhao, N. Zhao, R. P. Zhao, S. J. Zhao, Y. B. Zhao, Y. L. Zhao, Y. X. Zhao, Z. G. Zhao, A. Zhemchugov, B. Zheng, B. M. Zheng, J. P. Zheng, W. J. Zheng, X. R. Zheng, Y. H. Zheng, B. Zhong, C. Zhong, H. Zhou, J. Q. Zhou, J. Y. Zhou, S. Zhou, X. Zhou, X. K. Zhou, X. R. Zhou, X. Y. Zhou, Y. X. Zhou, Y. Z. Zhou, A. N. Zhu, J. Zhu, K. Zhu, K. J. Zhu, K. S. Zhu, L. Zhu, L. X. Zhu, S. H. Zhu, T. J. Zhu, W. D. Zhu, W. D. Zhu, W. J. Zhu, W. Z. Zhu, Y. C. Zhu, Z. A. Zhu, X. Y. Zhuang, J. H. Zou, J. Zu

**Affiliations:** 1 Institute of High Energy Physics, Beijing, People’s Republic of China; 2 Budker Institute of Nuclear Physics of Siberian Branch Russian Academy of Sciences (BINP SB RAS), Novosibirsk, Russia; 3 Novosibirsk State University, Novosibirsk, Russia; 4 Uppsala University, Uppsala, Sweden; 5 Zhengzhou University, Zhengzhou, People’s Republic of China; 6 Johannes Gutenberg University of Mainz, Mainz, Germany; 7 University of Turin, Turin, Italy; 8 INFN, Turin, Italy; 9 University of Science and Technology of China, Hefei, People’s Republic of China; 10 State Key Laboratory of Particle Detection and Electronics, Hefei, People’s Republic of China; 11 Southeast University, Nanjing, People’s Republic of China; 12 Joint Institute for Nuclear Research, Dubna, Russia; 13 Peking University, Beijing, People’s Republic of China; 14 State Key Laboratory of Nuclear Physics and Technology, Peking University, Beijing, People’s Republic of China; 15 University of Chinese Academy of Sciences, Beijing, People’s Republic of China; 16 National Centre for Nuclear Research, Warsaw, Poland; 17 Institute of Physics and Technology, Mongolian Academy of Sciences, Ulaanbaatar, Mongolia; 18 INFN Laboratori Nazionali di Frascati, Frascati, Italy; 19 INFN Sezione di Ferrara, Ferrara, Italy; 20 Carnegie Mellon University, Pittsburgh, PA USA; 21 University of Münster, Münster, Germany; 22 Wuhan University, Wuhan, People’s Republic of China; 23 Lanzhou University, Lanzhou, People’s Republic of China; 24 MOE Frontiers Science Center for Rare Isotopes, Lanzhou University, Lanzhou, People’s Republic of China; 25 Lanzhou Center for Theoretical Physics, Lanzhou University, Lanzhou, People’s Republic of China; 26 İstinye University, Istanbul, Turkey; 27 Nankai University, Tianjin, People’s Republic of China; 28 China University of Geosciences, Wuhan, People’s Republic of China; 29 Soochow University, Suzhou, People’s Republic of China; 30 Henan University, Kaifeng, People’s Republic of China; 31 Nanjing University, Nanjing, People’s Republic of China; 32 North China Electric Power University, Beijing, People’s Republic of China; 33 Tsinghua University, Beijing, People’s Republic of China; 34 Institute of Modern Physics, Lanzhou, People’s Republic of China; 35 Fudan University, Shanghai, People’s Republic of China; 36 Key Laboratory of Nuclear Physics and Ion-beam Application (MOE), Institute of Modern Physics, Fudan University, Shanghai, People’s Republic of China; 37 Jilin University, Changchun, People’s Republic of China; 38 Guangxi University of Science and Technology, Liuzhou, People’s Republic of China; 39 Hunan Normal University, Changsha, People’s Republic of China; 40 Hunan University, Changsha, People’s Republic of China; 41 School of Physics and Electronics, Hunan University, Changsha, China; 42 Sun Yat-sen University, Guangzhou, People’s Republic of China; 43 Chung-Ang University, Seoul, Republic of Korea; 44 HH Wills Physics Laboratory, University of Bristol, Bristol, UK; 45 Shandong University, Jinan, People’s Republic of China; 46 Yunnan University, Kunming, People’s Republic of China; 47 Helmholtz Institute Mainz, Mainz, Germany; 48 Ruhr University Bochum, Bochum, Germany; 49 University of South China, Hengyang, People’s Republic of China; 50 University of Jinan, Jinan, People’s Republic of China; 51 Liaoning University, Shenyang, People’s Republic of China; 52 Inner Mongolia University, Hohhot, People’s Republic of China; 53 Moscow Institute of Physics and Technology, Moscow, Russia; 54 Henan Normal University, Xinxiang, People’s Republic of China; 55 University of Eastern Piedmont, Alessandria, Italy; 56 Nanjing Normal University, Nanjing, People’s Republic of China; 57 University of Ferrara, Ferrara, Italy; 58 University of Manchester, Manchester, UK; 59 University of Oxford, Oxford, UK; 60 GSI Helmholtz Centre for Heavy Ion Research GmbH, Darmstadt, Germany; 61 Guangxi University, Nanning, People’s Republic of China; 62 Shandong Normal University, Jinan, People’s Republic of China; 63 Indiana University, Bloomington, IN USA; 64 University of Hawai‘i, Honolulu, HI USA; 65 Suranaree University of Technology, Nakhon Ratchasima, Thailand; 66 South China Normal University, Guangzhou, People’s Republic of China; 67 Guangdong Provincial Key Laboratory of Nuclear Science, Institute of Quantum Matter, South China Normal University, Guangzhou, China; 68 University of the Punjab, Lahore, Pakistan; 69 Hangzhou Normal University, Hangzhou, People’s Republic of China; 70 Huangshan College, Huangshan, People’s Republic of China; 71 Instituto de Alta Investigación, Universidad de Tarapacá, Arica, Chile; 72 University of Groningen, Groningen, The Netherlands; 73 Indian Institute of Technology Madras, Chennai, India; 74 II. Physikalisches Institut, Justus-Liebig-Universität Gießen, Giessen, Germany; 75 Qufu Normal University, Qufu, People’s Republic of China; 76 Liaoning Normal University, Dalian, People’s Republic of China; 77 Renmin University of China, Beijing, People’s Republic of China; 78 Hebei University, Baoding, People’s Republic of China; 79 China Center of Advanced Science and Technology, Beijing, People’s Republic of China; 80 Sichuan University, Chengdu, People’s Republic of China; 81 Guangxi Normal University, Guilin, People’s Republic of China; 82 Shanxi University, Taiyuan, People’s Republic of China; 83 Central China Normal University, Wuhan, People’s Republic of China; 84 Henan University of Science and Technology, Luoyang, People’s Republic of China; 85 Henan University of Technology, Zhengzhou, People’s Republic of China; 86 Central South University, Changsha, People’s Republic of China; 87 Zhejiang University, Hangzhou, People’s Republic of China; 88 Hangzhou Institute for Advanced Study, University of Chinese Academy of Sciences, Hangzhou, China; 89 Goethe University Frankfurt, Frankfurt am Main, Germany; 90 COMSATS University Islamabad, Lahore Campus, Lahore, Pakistan; 91 Department of Mathematical Sciences, Institute of Business Administration, Karachi, Karachi, Pakistan; 92 INFN Sezione di Perugia, Perugia, Italy; 93 University of Perugia, Perugia, Italy; 94 NRC “Kurchatov Institute”, Petersburg Nuclear Physics Institute (PNPI), Gatchina, Russia; 95 Ecole Polytechnique Federale de Lausanne (EPFL), Lausanne, Switzerland; 96 Shanghai Jiao Tong University, Shanghai, People’s Republic of China; 97 Key Laboratory for Particle Physics, Astrophysics and Cosmology, Ministry of Education; Shanghai Key Laboratory for Particle Physics and Cosmology; Institute of Nuclear and Particle Physics, Shanghai, People’s Republic of China; 98 Near East University, Nicosia, Turkey; 99 Southwest University of Science and Technology, Mianyang, People’s Republic of China; 100 Beihang University, Beijing, People’s Republic of China; 101 Yantai University, Yantai, People’s Republic of China; 102 Shanxi Normal University, Linfen, People’s Republic of China; 103 University of Science and Technology Liaoning, Anshan, People’s Republic of China; 104 Present Address: Silesian University in Katowice, Chorzow, Poland

**Keywords:** Experimental particle physics, Experimental nuclear physics

## Abstract

The unitarity of the Cabibbo–Kobayashi–Maskawa (CKM) matrix is a cornerstone of the standard model (SM). Precise tests of this unitarity require independent determinations of its elements, such as |*V*_us_|, which governs the transition between strange and up quarks. Current measurements from kaon and tau decays show tensions that may hint at physics beyond the SM^1–3^. Hyperon semileptonic decays provide alternative probes but have remained largely untapped because previous experiments lacked sufficient kinematic information, making the measurements insensitive to the relevant form factors^4^. Here we report measurements of the axial-vector and weak-magnetism couplings, as well as the first determinations of the absolute branching fraction and weak-electricity coupling in $\Lambda \to {{\rm{pe}}}^{-}{\bar{\nu }}_{{\rm{e}}}$, achieved by exploiting the polarization and quantum entanglement of $\Lambda \bar{\Lambda }$ pairs produced at the J/ψ resonance. Combining our results with recent lattice quantum chromodynamics (QCD) calculations^5^ gives |*V*_us_|_LQCD_ = 0.2339 ± 0.0041, a model-independent determination consistent with CKM unitarity. By pioneering the exploitation of polarization and quantum entanglement in baryon semileptonic decays, our method enhances single-event sensitivity and is broadly applicable to other baryon semileptonic decays, establishing the foundation for a systematic research programme that can achieve precision comparable with that of kaon decays and provide a stringent independent test of the SM.

## Abstract (web summary)

Polarization and quantum entanglement measurements of baryon semileptonic decay greatly enhance single-event sensitivity, and combined with lattice quantum chromodynamics calculations enable determination of the |*V*_us_| element of the Cabibbo–Kobayashi–Maskawa matrix, providing an independent test of the standard model.

## Main

**Fig. 1:**
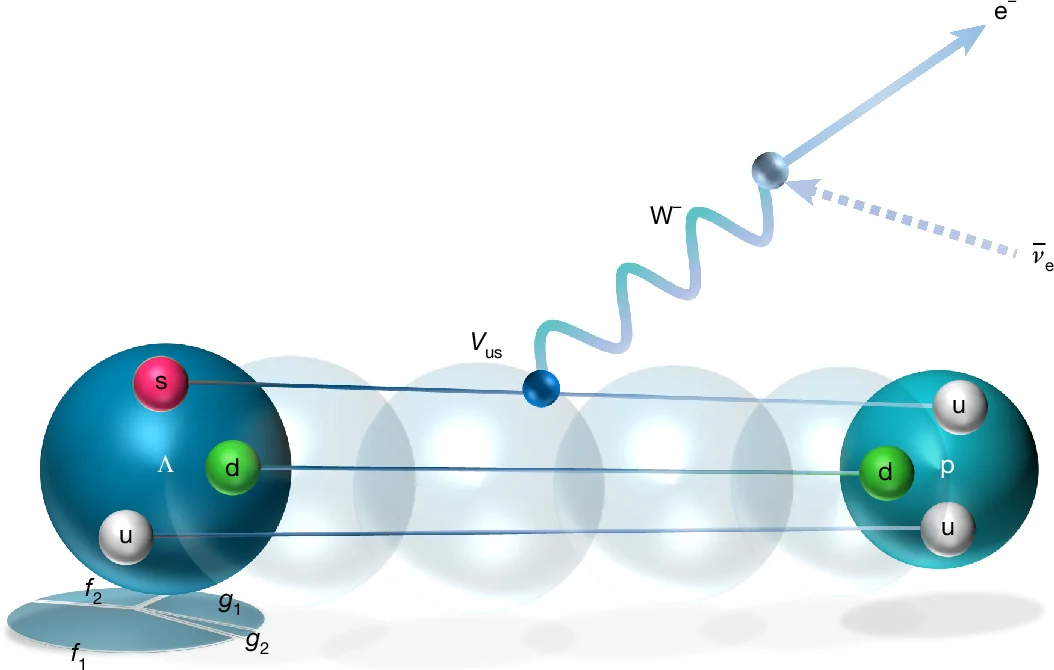
Schematic representation of the Λ decay to a proton, an electron and an electric antineutrino through virtual (mediating) W^−^ boson. The pie chart shows the relevant magnitudes of the dominant vector and axial-vector form factors partaking in the transition.

Transition form factors are fundamental hadron properties describing the dynamic behaviour of the transition between two states. The transition matrix element for a semileptonic weak decay of a hyperon is parametrized by six form factors, *f*_*i*_(*q*^2^) and *g*_*i*_(*q*^2^) (*i* = 1, 2, 3). These represent the vector and axial vector parts of the weak interaction, respectively, and depend on the squared momentum transfer, *q*^2^, of the intermediate W boson. The vector form factors are commonly denoted as the vector *f*_1_(*q*^2^), weak magnetism *f*_2_(*q*^2^) and scalar *f*_3_(*q*^2^) form factors. The axial-vector form factors are further subdivided into the axial *g*_1_(*q*^2^), weak electricity *g*_2_(*q*^2^) and pseudoscalar *g*_3_(*q*^2^) form factors^6^. Each form factor is associated with a specific current and encodes information about the underlying hadronic structure. Because both the scalar and the pseudoscalar form factors are suppressed by the squared ratio of the lepton to hyperon mass, the electron–antineutrino decay modes effectively depend only on four form factors and *V*_us_, as depicted in Fig. 1.

When neglecting the *q*^2^ dependence of the form factors, the branching ratio ${\mathcal{B}}$ of the semileptonic decay $\Lambda \to {{\rm{pe}}}^{-}{\bar{\nu }}_{{\rm{e}}}$ is given by^6^:

1 $${\mathcal{B}}=\frac{{\tau }_{\Lambda }}{\hbar }{G}_{{\rm{F}}}^{2}|{V}_{{\rm{us}}}{|}^{2}\frac{{\beta }^{5}{{M}_{\Lambda }}^{5}}{60{{\rm{\pi }}}^{3}}\left[\left(1-\frac{3}{2}\beta \right)(\,{f}_{1}^{2}+3{g}_{1}^{2})-4\beta {g}_{1}{g}_{2}+\frac{2}{7}{\beta }^{2}{{\mathcal{F}}}_{2}+{\mathcal{O}}({\beta }^{3})\right],$$

in which *β* = (*M*_Λ_ − *M*_p_)/*M*_Λ_ ≈ 0.159, with *M*_Λ_ and *M*_p_ denoting the masses of the Λ and proton, respectively. The weak decay constant *G*_F_ and the Λ lifetime *τ*_Λ_ are known with high accuracy^4^. The form-factor-dependent function ${{\mathcal{F}}}_{2}$ is defined as:

2 $${{\mathcal{F}}}_{2}=3{f}_{1}^{2}+3{f}_{1}{f}_{2}+2{{f}_{2}}^{2}+6{{g}_{1}}^{2}+6{g}_{2}^{2}+21{g}_{1}{g}_{2}.$$

The ratios *g*_1_/*f*_1_ (throughout this paper, *f*_*i*_ ≡ *f*_*i*_(*q*^2^ = 0) and *g*_*i*_ ≡ *g*_*i*_(*q*^2^ = 0) are implied, unless explicitly noted), *f*_2_/*f*_1_ and *g*_2_/*f*_1_ represent the axial-vector (*g*_av_ ≡ *g*_1_/*f*_1_), weak-magnetism (*g*_w_ ≡ *f*_2_/*f*_1_) and weak-electricity (*g*_av2_ ≡ *g*_2_/*f*_1_) couplings at zero momentum transfer *q*^2^, respectively. Therefore, a precise measurement of the decay branching fraction can be used to determine |*V*_us_| if the form factors are known. The relative couplings *g*_av_, *g*_w_ and *g*_av2_ can be determined from kinematic angular variables, but a reliable theoretical determination of *f*_1_ requires an understanding of the subtle differences between the d and s quarks^7^. In this context, approximate flavour SU(3) symmetry serves as a good approximation, as confirmed by previous experimental results^8–10^. This assumption treats the s and d quarks as equivalent, with symmetry breaking arising from their small mass difference relative to both *M*_Λ_ and *M*_p_. For instance, *f*_1_ is protected from leading-order SU(3)-breaking effects^11^. However, corrections are essential at next-to-leading order. There are various model estimates of *f*_1_ using quark models^12,13^, large-*N*_c_ (refs. ^14–17^), chiral expansions^18–20^ and QCD sum rules^21^. A drawback is that these models disagree on the size of the SU(3)-breaking corrections, which affects the precision of |*V*_us_| (ref. ^22^). During the past decade, the lattice QCD community has joined the efforts of determining *f*_1_ using a non-perturbative method^5,23,24^ and a model-independent approach^25^ developed for the form factors of meson decays^26,27^. Still, understanding the quark mass dependence of SU(3)-breaking remains a crucial issue.

Another way to determine the vector form factor is indirectly through the *g*_av_ coupling and *g*_1_ form factor. The axial-vector coupling has been measured experimentally^8–10^, with the most precise determination, *g*_av_ = 0.719 ± 0.016 ± 0.012, performed more than 30 years ago by a Fermilab-based fixed-target experiment using a neutral-hyperon beam^10^, which observed approximately 37 × 10^3^ $\Lambda \to {{\rm{pe}}}^{-}{\bar{\nu }}_{{\rm{e}}}$ candidates. The average value based on the Particle Data Group gives *g*_av_ = 0.718 ± 0.015 (ref. ^4^). In contrast to *f*_1_, the axial-vector form factor is not protected by the Ademollo–Gatto theorem^11^. Thus, SU(3)-breaking corrections must be considered at leading order. During the past several years, the *g*_1_ of hyperon beta decays have been calculated with high accuracy from first principles using the techniques of lattice QCD^28^. Although the hyperon axial form factor can be determined from the lattice, there is at present not enough experimental information. The weak-electricity coupling, *g*_av2_, is intimately linked to SU(3) symmetry. In the absence of second-class currents—which are characterized by their G-parity asymmetric transformation—it vanishes in the SU(3) limit^29^. This quantity has so far only been determined once by the KTeV experiment for the process ${\Xi }^{0}\to {\Sigma }^{+}{{\rm{e}}}^{-}{\bar{\nu }}_{{\rm{e}}}$ decay^30^ and was found to be consistent with zero.

The CKM matrix element |*V*_us_| was evaluated by Cabibbo using the Λ semileptonic decay^7,31^. The analyses were based on the determination of relative branching fractions^8^ and the *g*_av_ coupling^10^, whereas the values of *f*_2_ and *g*_2_ were predicted within the conserved vector current hypothesis^32^ and SU(3) symmetry limit^7,31^, respectively. The obtained precision was found to be *σ*(|*V*_us_|) ≈ 0.0034, for which precise experimental measurements and methods are required to be competitive with the *V*_us_ determined from kaon decays, *σ*(|*V*_us_|) ≈ 0.0009 (ref. ^4^). Thus, in comparison with the traditional way of determining |*V*_us_| using kaon decays, in which the accuracy is limited by knowledge of decay constants in lattice QCD calculation^33^, more studies are required for the relevant transition matrix elements to reach a comparable level^33^.

In this article, we determine the absolute branching fraction for $\Lambda \to {{\rm{pe}}}^{-}{\bar{\nu }}_{{\rm{e}}}$ and the couplings *g*_av_, *g*_w_ and *g*_av2_ simultaneously for the first time. Meanwhile, two values of |*V*_us_| are provided, one under the assumption of SU(3) symmetry conservation and the other using the lattice QCD determination of the form factors. Furthermore, the product of |*V*_us_| and the dominant form-factor combination, $|{V}_{{\rm{us}}}|\cdot \sqrt{{f}_{1}^{2}+3{g}_{1}^{2}}$, has, to our knowledge, been extracted for the first time for any semileptonic baryon decay.

**Fig. 2:**
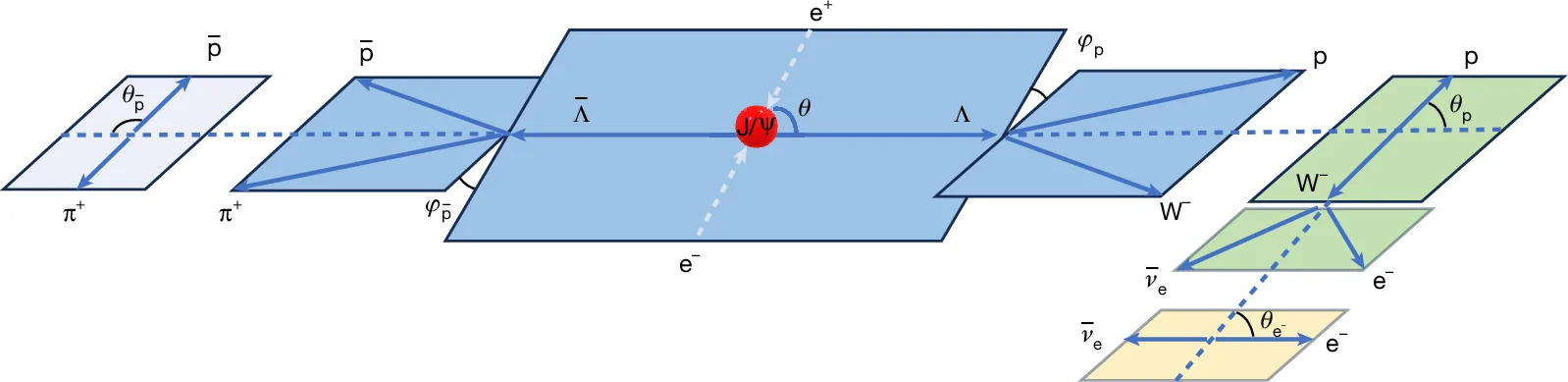
Definition of the helicity angles for ${{\bf{e}}}^{{\boldsymbol{+}}}{{\bf{e}}}^{{\boldsymbol{-}}}{\boldsymbol{\to }}{\bf{J}}/{\boldsymbol{\psi }}{\boldsymbol{\to }}{\boldsymbol{\Lambda }}({\boldsymbol{\to }}{{\bf{pe}}}^{{\boldsymbol{-}}}{\bar{{\boldsymbol{\nu }}}}_{{\bf{e}}}{\boldsymbol{)}}\bar{{\boldsymbol{\Lambda }}}{\boldsymbol{(}}{\boldsymbol{\to }}\bar{{\bf{p}}}{{\boldsymbol{\pi }}}^{{\boldsymbol{+}}}{\boldsymbol{)}}$. The angles *θ*, ${\theta }_{\bar{{\rm{p}}}}$, ${\varphi }_{\bar{{\rm{p}}}}$, *θ*_p_, *φ*_p_ and *θ*_e_ are the helicity angles of the Λ, $\bar{{\rm{p}}}$, p and lepton in the e^+^e^−^ c.m. system (blue plane), $\bar{\Lambda }$ rest frame (grey plane), Λ rest frame (green plane) and W^−^ rest frame (yellow plane), respectively.

The method, developed in refs. ^34,35^, first exploits entangled polarized baryon–antibaryon pairs from the process ${{\rm{e}}}^{+}{{\rm{e}}}^{-}\to {\rm{J}}/{\rm{\psi }}\to$ $\Lambda (\to {{\rm{pe}}}^{-}{\bar{\nu }}_{{\rm{e}}})\bar{\Lambda }(\to \bar{{\rm{p}}}{{\rm{\pi }}}^{+})$ with its corresponding charge-conjugated $\Lambda \bar{\Lambda }$ decay modes. The production of the Λ pairs with their subsequent decays are described with seven kinematic variables: six helicity angles and the four-momentum transfer between the Λ and proton, $\xi =\{\theta ,{\theta }_{{\rm{p}}},{\varphi }_{{\rm{p}}},{\theta }_{{\rm{e}}},{\theta }_{\bar{{\rm{p}}}},{\varphi }_{\bar{{\rm{p}}}},{q}^{2}\}$. The helicity angles are determined by boosting and rotating the particles into their respective helicity frames (Fig. 2). The production process is characterized by the Λ scattering angle relative to the positron beam axis in the centre-of-momentum (c.m.) frame, *θ*. The angles *θ*_p_ and *φ*_p_ (${\theta }_{\bar{{\rm{p}}}}$ and ${\varphi }_{\bar{{\rm{p}}}}$) are defined with respect to the proton (antiproton) direction in the reference system ${{\mathcal{R}}}_{\Lambda }({{\mathcal{R}}}_{\bar{\Lambda }})$, in which $\Lambda (\bar{\Lambda })$ is at rest and where the $\hat{z}$ axis points in the direction of the $\Lambda (\bar{\Lambda })$ in the c.m. system. The $\hat{y}$ axis is perpendicular to the production plane. The angle *θ*_e_ and the four-momentum transfer *q*^2^ give the direction of the electron (positron) in the direction of the $\Lambda (\bar{\Lambda })$ in the ${{\mathcal{R}}}_{\Lambda }$ (${{\mathcal{R}}}_{\bar{\Lambda }}$) frame. The structure of the seven-dimensional distribution of the kinematic variables is determined by six global parameters **ω** = {*α*_ψ_, Δ*Φ*, *g*_av_, *g*_w_, *g*_av2_, *α*_+_(*α*_−_)} and can be expressed in a modular form as^35^:

3 $${\mathcal{W}}(\xi ;\omega )=\mathop{\sum }\limits_{\mu ,\bar{\nu }=0}^{3}{C}_{\mu \bar{\nu }}{R}_{\mu \kappa }({\Omega }_{{\rm{p}}}){b}_{\kappa 0}^{\Lambda }{a}_{\bar{\nu }0}^{\bar{\Lambda }}+c.c.,$$

The ${\mathcal{W}}$ denotes the weights used in the maximum log-likelihood fit, as defined in Methods, for determining the form factors of interest. The production term ${C}_{\mu \bar{\nu }}(\theta ;{\alpha }_{{\rm{\psi }}},\Delta \varPhi )$ is a 4 × 4 spin density matrix describing the polarization and the spin correlations between the Λ pair and is defined in the reference frames ${{\mathcal{R}}}_{\Lambda }$ and ${{\mathcal{R}}}_{\bar{\Lambda }}$. The parameters *α*_ψ_ and Δ*Φ* are related to two production amplitudes, in which *α*_ψ_ governs the Λ angular distribution and sin(Δ*Φ*) is proportional to the hyperon polarization^34^. The 4 × 4 matrices ${R}_{\mu \kappa }{b}_{\kappa 0}^{\Lambda }$ in equation (3) represents the rotation and decay matrices between the Λ and the proton in the semileptonic decay, respectively, whereas matrix ${a}_{\bar{\nu }0}^{\bar{\Lambda }}$ describes the transition from the $\bar{\Lambda }$ to the antiproton in the non-leptonic decay by means of the asymmetry parameter *α*_+_ (*α*_−_ counts for Λ → p transition)^36^. The elements of these matrices are parametrized in terms of the helicity angles as well as the weak decay parameters ${R}_{\mu \kappa }({\theta }_{{\rm{p}}},{\varphi }_{{\rm{p}}}){b}_{\kappa 0}^{\Lambda }({\theta }_{{\rm{e}}},{q}^{2};{g}_{{\rm{av}}},{g}_{{\rm{w}}},{g}_{{\rm{av}}2})$ in reference system ${{\mathcal{R}}}_{\Lambda }$ and ${a}_{\bar{\nu }0}^{\bar{\Lambda }}({\theta }_{\bar{{\rm{p}}}},{\varphi }_{\bar{{\rm{p}}}};{\alpha }_{+})$ in reference system ${{\mathcal{R}}}_{\bar{\Lambda }}$. The full expressions of ${C}_{\mu \bar{\nu }}$, *R*_*μκ*_*b*_*κ*0_ and ${a}_{\bar{\nu }0}$ are given in refs. ^34,35^. Details of the coordinate system are provided in Methods.

**Fig. 3:**
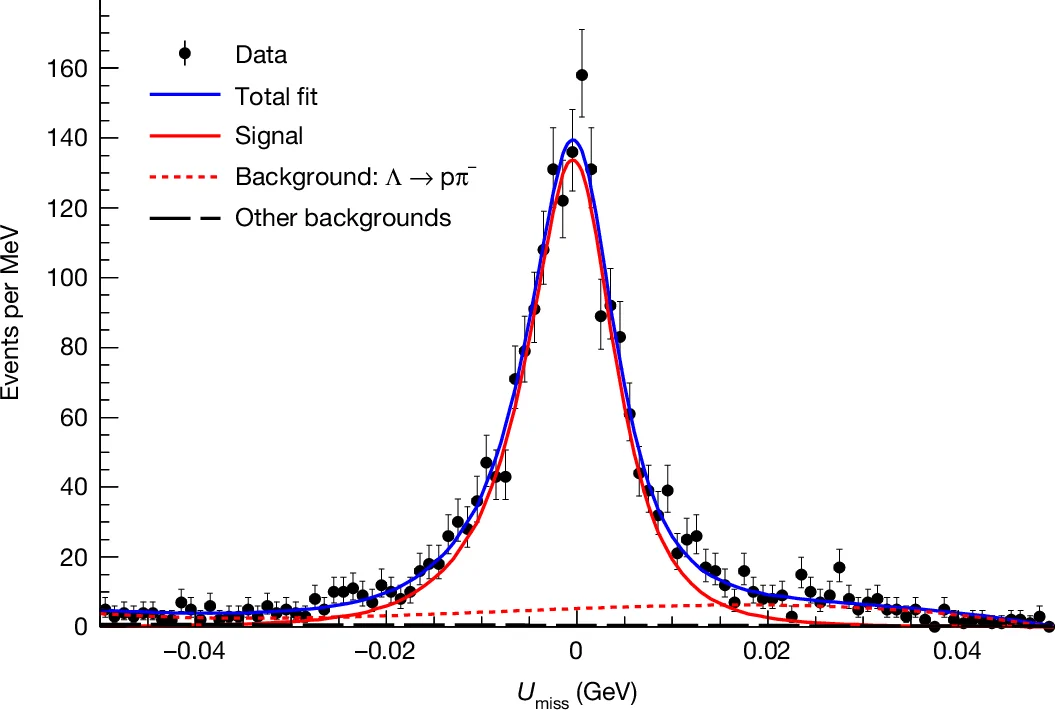
The *U*_miss_ distribution of ${\boldsymbol{\Lambda }}{\boldsymbol{\to }}{{\bf{pe}}}^{{\boldsymbol{-}}}{\bar{{\boldsymbol{\nu }}}}_{{\bf{e}}}$ and $\bar{{\boldsymbol{\Lambda }}}{\boldsymbol{\to }}\bar{{\bf{p}}}{{\bf{e}}}^{{\boldsymbol{+}}}{{\boldsymbol{\nu }}}_{{\bf{e}}}$ candidates. The points with error bars represent the observed number of events in each bin, with statistical uncertainties. The solid blue line shows the total fit, which includes the signal candidates (solid red line), the dominant background (dashed red line) and the other background contributions (long-dashed black line).

Our results are based on the (10.087 ± 0.044) × 10^9^ J/ψ events collected with the multipurpose detector Beijing Spectrometer III (BESIII; ref. ^37^). The J/ψ resonance decays into a $\Lambda \bar{\Lambda }$ pair with a probability of (1.89 ± 0.09) × 10^−3^ (ref. ^4^). All final-state particles except the neutrino are electrically charged and can therefore be reconstructed in the multilayer drift chamber (MDC), in which a superconducting solenoid provides a magnetic field enabling momentum determination with an accuracy of 0.5% at 1.0 GeV *c*^−1^. The semileptonic and hadronic Λ ($\bar{\Lambda }$) candidates are identified by combining ${{\rm{pe}}}^{-}\,(\bar{{\rm{p}}}{{\rm{e}}}^{+})$ pairs and $\bar{{\rm{p}}}{{\rm{\pi }}}^{+}\,({\rm{p}}{{\rm{\pi }}}^{-})$, respectively. To obtain electrons/positrons with sufficient quality, their momenta were required to be larger than 0.1 GeV *c*^−2^. The semileptonic decays are identified using the variable ${U}_{{\rm{miss}}}={E}_{{\rm{miss}}}-c\cdot |{\vec{p}}_{{\rm{miss}}}|$, in which *E*_miss_ and ${\vec{p}}_{{\rm{miss}}}$ denote the missing energy and momentum of the semileptonic decay, respectively. For candidates of semileptonic decay, the *U*_miss_ distribution is uniformly distributed around zero, whereas the hadronic Λ background features a right-skewed distribution that peaks at *U*_miss_ ≈ 0.022 GeV. The signal yield is determined from an extended unbinned maximum likelihood fit^38^ of the *U*_miss_ distribution, shown in Fig. 3, which combines both $\Lambda \to {{\rm{pe}}}^{-}{\bar{\nu }}_{{\rm{e}}}$ and $\bar{\Lambda }\to \bar{{\rm{p}}}{{\rm{e}}}^{+}{\nu }_{{\rm{e}}}$. The signal shape is described using Monte Carlo (MC) simulation convoluted with a Gaussian function to account for the resolution differences between data and MC. The shape of the dominant background contribution, Λ → pπ^−^, is obtained using the corresponding MC sample, whereas the rest of the background contribution is described by a linear function. The yields and the parameters of the Gaussian and linear functions are floated in the fit. More details of the selection and analysis procedure are given in Methods.

For the determination of the semileptonic absolute branching fraction, we use the double-tagging technique, as pioneered in the MARK-III experiment^39^. A double-tag event means that one Λ is reconstructed as $\Lambda \to {\rm{pe}}{\bar{\nu }}_{{\rm{e}}}$ and the antihyperon partner as $\bar{\Lambda }\to \bar{{\rm{p}}}{{\rm{\pi }}}^{+}$. The technique also requires knowledge of the single-tag sample, which means that only one side is reconstructed as $\bar{\Lambda }\to \bar{{\rm{p}}}{{\rm{\pi }}}^{+}$. It has the extra advantage that the four-momentum transfer *q*^2^ can be reconstructed unambiguously^40^. A more detailed account of the technique is provided in Methods. The branching fraction of the semileptonic decay of the Λ is determined from

4 $${\mathcal{B}}(\Lambda \to {{\rm{pe}}}^{-}{\bar{\nu }}_{{\rm{e}}})=\frac{{N}_{{\rm{DT}}}}{{{\epsilon }}_{{\rm{DT}}}}\frac{{{\epsilon }}_{{\rm{ST}}}}{{N}_{{\rm{ST}}}},$$

in which *N*_DT_ and *ϵ*_DT_ are the semileptonic event yield and detection efficiency in the double-tagging sample, respectively, and *N*_ST_ and *ϵ*_ST_ are the event yield and detection efficiency obtained using the single-tagging sample. The single-tagging yield and efficiency are determined using the same methods from ref. ^41^, in which they are obtained from a fit to the invariant mass distribution of the single-tagging candidate distribution. These are found to be *N*_ST_ = (14.328 ± 0.005) × 10^6^ and *ϵ*_ST_ = (54.09 ± 0.01)%, respectively. The signal event yield, *N*_DT_ = 1,854 ± 49, is obtained from the fit in Fig. 3. The double-tagging detection efficiency, *ϵ*_DT_ = (8.58 ± 0.01)%, is determined from a dedicated simulated sample including the production and decay dynamics of the full process. Equation (4) yields ${\mathcal{B}}(\Lambda \to {{\rm{pe}}}^{-}{\bar{\nu }}_{{\rm{e}}})=(8.16\,\pm \,0.22\,\pm \,0.15)\times 1{0}^{-4}$, in which the first uncertainty is statistical and the second is systematic, discussed in Methods. This is in agreement with the world average ${\mathcal{B}}(\Lambda \to {{\rm{pe}}}^{-}{\bar{\nu }}_{{\rm{e}}})=(8.34\,\pm \,0.14)\times 1{0}^{-4}$ (ref. ^4^).

Whereas the branching fraction determination requires only yields and efficiencies, the extraction of the form factors requires a detailed understanding of the decay kinematics. To increase the sample purity, we retain only events with |*U*_miss_| < 0.02 GeV, resulting in 1,791 ± 47 signal and 207 ± 30 background events. From these events, we reconstruct the kinematic variables **ξ** and determine the transition form factors in **ω** using an unbinned maximum log-likelihood fit that accounts for the multidimensional reconstruction efficiency. In contrast to previous measurements^8–10^, we can exploit the fact that the Λ is produced along with a $\bar{\Lambda }$ to our advantage. On an event-by-event basis, it allows us to fully reconstruct the kinematics of the semileptonic decay, whereas globally, it provides detailed information on the production mechanism of the quantum-entangled $\Lambda -\bar{\Lambda }$ pair. The Λ polarization and the spin correlations between the pair are uniquely determined by the two production parameters, *α*_ψ_ and Δ*Φ*. These, along with the two decay parameters, *α*_−_ and *α*_+_, have already been precisely measured using the same dataset in refs. ^36,42^. Therefore, the free physics parameters in **ω** are reduced to three from six—the couplings *g*_av_, *g*_w_ and *g*_av2_. These couplings can therefore be determined with optimal precision, even with a relatively modest data sample. The details of the maximum log-likelihood fit procedure and the systematic uncertainties are described in Methods.

The final results for the axial-vector and weak-magnetism couplings are summarized in Table 1. They are determined separately for the e^±^ semileptonic channels and simultaneously by assuming ${g}_{i}^{-}=-{g}_{i}^{+}$, *i* = {av, av2} and ${g}_{{\rm{w}}}^{-}={g}_{{\rm{w}}}^{+}$. Two results are provided: one in which the weak-electricity coupling is fixed to the SU(3) symmetry limit^7,31^, *g*_av2_ = 0, and the other in which it is determined along with the other two couplings. The first result allows for a more precise extraction of *g*_av_ and *g*_w_. The value of the weak-magnetism coupling, ⟨*g*_w_⟩ = 0.89 ± 0.38, is consistent with both the conserved vector current hypothesis prediction of 0.97 (ref. ^32^) and the previous measurement of *g*_w_ = 0.15 ± 0.30 (ref. ^10^), with the latter agreement at the 1.5*σ* level. The uncertainty achieved for ⟨*g*_w_⟩ in our analysis is comparable with that of the Fermilab measurement^10^. For the second set, the weak-electricity coupling is found to be $\langle {g}_{{\rm{av}}2}\rangle =-0.1{9}_{-0.63}^{+0.65}\,\pm \,0.18$, providing the first measurement for Λ semileptonic decay. It is also three times more precise compared with the KTeV determination of *g*_av2_ for the Ξ^0^ → Σ^+^ semileptonic decay^30^. The corresponding average axial-vector and weak-magnetism couplings are $\langle {g}_{{\rm{av}}}\rangle =0.70{6}_{-0.086}^{+0.089}$ and $\langle {g}_{{\rm{w}}}\rangle =0.7{7}_{-0.49}^{+0.53}$, consistent with the first set of results within the uncertainties; both are also compatible with the corresponding lattice QCD calculation^5^.

**Table 1:** Summary of the results

Observable	This work	Previous result	References
${\mathcal{B}}(\Lambda \to {{\rm{pe}}}^{-}{\bar{\nu }}_{{\rm{e}}})$	(8.16 ± 0.22 ± 0.15) × 10^−4^	(8.34 ± 0.14) × 10^−4^	^4^
${g}_{{\rm{av}}}^{-}$	$0.74{2}_{-0.057}^{+0.075}\pm 0.009$	0.718 ± 0.015	^4^
${g}_{{\rm{av}}}^{+}$	$-0.70{6}_{-0.069}^{+0.073}\pm 0.014$	–	
⟨*g*_av_⟩	$0.72{9}_{-0.047}^{+0.048}\pm 0.007$	–	
${g}_{{\rm{w}}}^{-}$	0.93 ± 0.51 ± 0.17	0.15 ± 0.30	^10^
${g}_{{\rm{w}}}^{+}$	0.89 ± 0.49 ± 0.20	–	
⟨*g*_w_⟩	0.89 ± 0.35 ± 0.14	–	
|*V*_us_|_SU(3)_	0.2194 ± 0.0094	0.2224 ± 0.0034	^7,31^
|*V*_us_|_LQCD_	0.2339 ± 0.0041		
$|{V}_{{\rm{us}}}|\cdot \sqrt{{f}_{1}^{2}+3{g}_{1}^{2}}$	0.4496 ± 0.0075	–	
⟨*g*_av_⟩	$0.70{6}_{-0.086}^{+0.089}\pm 0.026$	–	
⟨*g*_w_⟩	$0.7{7}_{-0.49}^{+0.53}\pm 0.14$	–	
⟨*g*_av2_⟩	$-0.1{9}_{-0.63}^{+0.65}\pm 0.18$	–	

The branching fraction ${\mathcal{B}}$ of $\Lambda \to {{\rm{pe}}}^{-}{\bar{\nu }}_{{\rm{e}}}$ and form-factor ratios for $\Lambda \to {{\rm{pe}}}^{-}{\bar{\nu }}_{{\rm{e}}}({g}_{{\rm{av}}}^{-},{g}_{{\rm{w}}}^{-})$, $\bar{\Lambda }\to \bar{{\rm{p}}}{{\rm{e}}}^{+}{\nu }_{{\rm{e}}}({g}_{{\rm{av}}}^{+},{g}_{{\rm{w}}}^{+})$ and the average ⟨*g*_av_⟩ and ⟨*g*_w_⟩ with the constraint that *g*_av2_ = 0, in which the first and second uncertainties are statistical and systematic, respectively; the value of |*V*_us_| within the SU(3) symmetry limit and with the lattice QCD prediction, the product of the |*V*_us_| and the form factors $|{V}_{{\rm{us}}}|\cdot \sqrt{{f}_{1}^{2}+3{g}_{1}^{2}}$, in which the uncertainties are total uncertainties calculated as a sum in quadrature of the individual uncertainties; the average ⟨*g*_av_⟩ and ⟨*g*_w_⟩ with the average ⟨*g*_av2_⟩ result with statistical and systematic uncertainties, respectively.

To extract the CKM matrix element |*V*_us_| from equation (1), we use the Λ lifetime *τ*_Λ_ = (2.617 ± 0.010) × 10^−10^ s (ref. ^4^) and our determined ⟨*g*_av_⟩, ⟨*g*_w_⟩ and ${\mathcal{B}}(\Lambda \to {{\rm{pe}}}^{-}{\bar{\nu }}_{{\rm{e}}})$. Meanwhile, we assume *g*_2_ = 0 (ref. ^7^), which is consistent with our second set of form-factor results and also assumed by the previous experimental measurements^9,10,43^ and for the determination of |*V*_us_| (refs. ^7,31^). The term ${\mathcal{O}}({\beta }^{3})$ is ignored, as its contribution is negligible within present experimental accuracy. For the decay rate, we also need to account for radiative effects, which modify the decay rate by (1.6 ± 0.5) × 10^−2^ (ref. ^44^).

The final hurdle before obtaining |*V*_us_| is that either *f*_1_ or *g*_1_ must be explicitly provided, either from theory or lattice QCD. This is similar to kaon semileptonic decays, in which the corresponding form factor *f*_+_ is required as input^4^. As a reference value for our discussions, we assume that flavour SU(3) symmetry is conserved. Under this assumption, *f*_1_ takes on the value $-\sqrt{3/2}$ (ref. ^7^) and $|{V}_{{\rm{us}}}{|}_{{\rm{SU}}(3)}=0.2194\,\pm$ $0.003{6}_{\text{BESIII BF}}\,\pm \,0.008{7}_{\mathrm{BESIIIFF}}\,\pm \,0.000{4}_{{\tau }_{\Lambda }}\,\pm \,0.000{5}_{{\rm{RC}}}$. The first and second uncertainties are from our measurements of the branching fraction and form factors, respectively, the third from the Λ lifetime and the fourth is the radiative correction uncertainties^44^. This result is in good agreement with the value extracted by Cabibbo from the $\Lambda \to {{\rm{pe}}}^{-}{\bar{\nu }}_{{\rm{e}}}$ world average, |*V*_us_| = 0.2224 ± 0.0034 (ref. ^7^). The value is also consistent with the CKM unitarity requirement within 0.9 standard deviations, |*V*_us_| = 0.22799 ± 0.00137 (refs. ^4,45^). Various theoretical models account for the potential effects of flavour SU(3) symmetry breaking, therefore we require a more accurate input that reflects this fact. The recent lattice QCD result^5^ provides the first detailed determination of the relevant form factors. Using these values together with our measured branching fraction, we obtain $|{V}_{{\rm{us}}}{|}_{{\rm{LQCD}}}=0.2339\,\pm \,0.003{8}_{\text{BESIII BF}}\,\pm \,0.000{4}_{{\tau }_{\Lambda }}\,\pm \,0.000{6}_{{\rm{RC}}}$ $\pm \,0.001{1}_{{\rm{LQCD}}}$, in which the fourth uncertainty arises from the lattice QCD calculation.

**Fig. 4:**
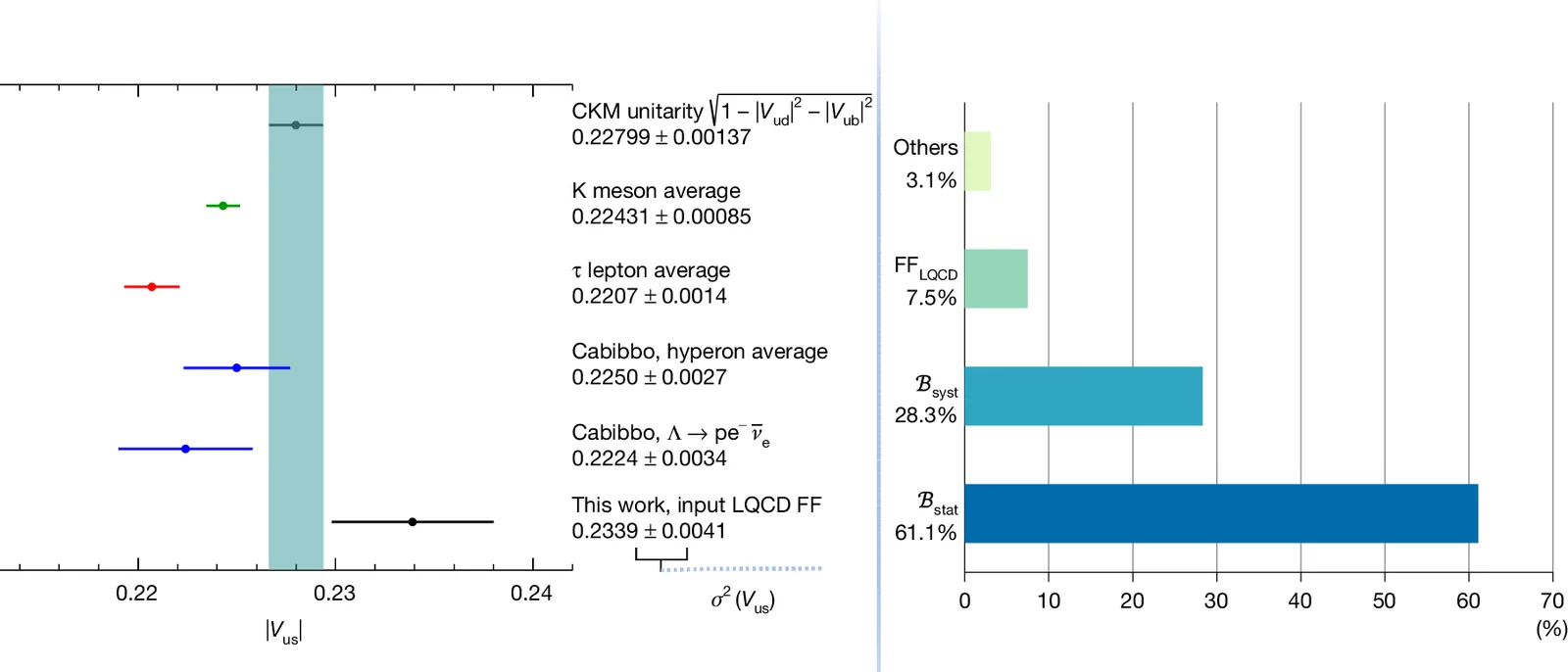
The |*V*_us_| result. Left, the first-row CKM unitarity constraint and *K* average are from ref. ^4^. The *τ* average is from the Heavy Flavor Averaging Group^45^. The two hyperon results were evaluated by Cabibbo^7,31^. Right, the contributing sources to the |*V*_us_| uncertainty, in terms of the variance.

The individual contributions to the uncertainty of this measurement are shown in Fig. 4. By using lattice-determined form factors, the corresponding contribution to the |*V*_us_| uncertainty is reduced from 84.9% to 7.5%. Although further improvements in the form factor remain, the statistical uncertainty of the branching fraction measurement, 0.0032, is now the dominant source. This demonstrates how the interplay between experimental measurements and lattice QCD calculations can enable increasingly precise determinations of CKM matrix elements. The combined BESIII and lattice QCD result, |*V*_us_|_LQCD_, is consistent with CKM unitarity at the 1.4*σ* level but is approximately two standard deviations larger than the value predicted by Cabibbo^7,31^, as shown in Fig. 4.

For future comparisons, we also provide an experimentally based result that does not rely on any theoretical form-factor input. The product of |*V*_us_| with the dominant form-factor contribution at zero momentum transfer, *q*^2^ = 0, is extracted as $|{V}_{{\rm{us}}}|\cdot \sqrt{{f}_{1}^{2}+3{g}_{1}^{2}}=0.4496\,\pm \,$ $0.006{1}_{{\text{BESIII BF}}_{{\rm{stat}}}}\,\pm \,0.004{1}_{{\text{BESIII BF}}_{{\rm{syst}}}}\,\pm \,0.000{9}_{{\tau }_{\Lambda }}\,\pm \,0.001{1}_{{\rm{RC}}}$. The reason for this is that, after assuming *g*_2_ = 0 and neglecting the ${{\mathcal{F}}}_{2}$ contribution, as the kinematic pre-factor is small, $\frac{2}{7}{\beta }^{2}\approx 0.007$, the partial decay width *Γ* is approximately proportional to ${V}_{{\rm{us}}}^{2}({f}_{1}^{2}+3{g}_{1}^{2})$. Under these two assumptions, the short-handed formula becomes identical to the standard next-to-leading order form in the lattice QCD framework^5^. As a technical side note, the radiative corrections applied to the weak decay constant differ between |*V*_us_|_SU(3)_ and |*V*_us_|_LQCD_. Consequently, when applying radiative corrections to the $|{V}_{{\rm{us}}}|\cdot \sqrt{{f}_{1}^{2}+3{g}_{1}^{2}}$, we use the same correction method as for |*V*_us_|_LQCD_. This product is model-independent, as it reduces reliance on theory-based form-factor inputs and thus provides a stringent constraint for validating theoretical descriptions of semileptonic baryon decays. To our knowledge, this is the first determination of such a product in any semileptonic baryon decay.

Summarizing our findings, using spin-entangled and polarized strange baryon–antibaryon pairs, we have reported the first absolute branching fraction determination for the semileptonic *e*-mode of strange baryon decays. We have determined the parameters related to the internal structure of the Λ baryon—the first measurement of the weak-electricity coupling *g*_av2_, as well as the axial-vector coupling *g*_av_ and the weak-magnetism coupling *g*_w_ in Λ semileptonic decay. We obtain two values for |*V*_us_|: one uses the SU(3)-symmetry-preserved vector form-factor value as normalization^46^ and the other uses form-factor input from lattice QCD calculations^5^. This is the first experimental determination for the |*V*_us_| after a more than 30-year break for the study of semileptonic Λ decay. The new modular approach^35^ is applied in the analysis, marking the first exploitation of polarization and quantum entanglement in baryon semileptonic decays. This innovative methodology greatly enhances the single-event sensitivity, as demonstrated by a direct comparison with the Fermilab measurement^10^: although their dataset is approximately 20 times larger than ours, we achieve a comparable precision for *g*_w_, whereas the uncertainty on *g*_av_ is only about a factor of 2.5 larger and, most notably, we determine *g*_av2_ for the first time, a parameter previously inaccessible. Along with the first exploitation of polarization and quantum entanglement in baryon semileptonic decays, this work establishes the foundation for a systematic research programme. The same method is already being extended to our following studies of other baryon semileptonic decays at BESIII (ref. ^47^) and applies to both present and future particle–antiparticle collider facilities, such as the upcoming PANDA experiment^48^ and the proposed super τ-charm factories^49,50^. Because form factors in different semileptonic decays are interrelated^7^, results obtained with this method can be combined across channels. Together with continued advances in lattice QCD^5^, this programme offers a clear path towards a model-independent determination of |*V*_us_| with precision competitive with that from kaon decays, thereby providing a critical and independent test of CKM unitarity.

## Methods

### Experimental apparatus

The BESIII detector^51^ records symmetric e^+^e^−^ collisions provided by the Beijing Electron–Positron Collider II (BEPCII) storage ring^52^. In this cylindrical system, tracks of charged particles in the detector are reconstructed from track-induced signals and their momenta are determined from the track curvature in the MDC. The flight time of the charged particles is recorded by a plastic scintillator time-of-flight subdetector. Electromagnetic showers from photons are reconstructed in the electromagnetic calorimeter. More details about the design and performance of the BESIII detector can be found in ref. ^51^.

### Monte Carlo simulation

For the selection, efficiency determination and background evaluation of the single-tag and double-tag processes, MC simulations have been used. The GEANT4-based package^53^ includes the geometric description and detector response of the BESIII detector. The inclusive MC sample includes both the production of the J/ψ resonance and the continuum processes incorporated in the KKMC simulation package^54^. All particle decays are modelled with EvtGen^55^ using the branching fractions from the Particle Data Group when available^4^ or else estimated with Lundcharm^56^. Final state radiation of the charged final state particles is simulated with PHOTOS^57^. The inclusive MC sample does not consider the decay dynamics and is thus only used for qualitative background studies. For the signal channel ${\rm{J}}/{\rm{\psi }}\to \Lambda (\to {{\rm{pe}}}^{-}{\bar{\nu }}_{{\rm{e}}})\bar{\Lambda }(\to \bar{{\rm{p}}}{{\rm{\pi }}}^{+})+c.c.$ and the background ${\rm{J}}/{\rm{\psi }}\to \Lambda (\to {\rm{p}}{{\rm{\pi }}}^{-})\bar{\Lambda }(\to \bar{{\rm{p}}}{{\rm{\pi }}}^{+})$, dedicated MC samples were produced. Also, these two modes include realistic production and decay dynamics using the physics parameter values, in agreement with the results in Table 1 and in ref. ^36^.

### Event selection criteria

For the single-tagged $\bar{\Lambda }(\Lambda )$ sample, the same selection procedure is applied as in ref. ^41^. The double-tag candidates are selected from the remaining tracks recoiling with respect to the single-tag candidates. All charged tracks must satisfy |cos*θ*_LAB_| < 0.93, in which *θ* is defined with respect to the *z*-axis, which is the symmetry axis of the MDC. Only events with four charged tracks are considered further. For the Λ reconstruction, the pair of charged tracks is constrained to originate from a common vertex. Furthermore, the decay length of the Λ candidate is required to be greater than zero. To identify whether a charged track is an electron/positron or a pion, particle identification is required. Each track is assigned a likelihood based on the MDC ionization energy loss and information from the time-of-flight subdetector and electromagnetic calorimeter subsystems. The charged track on the double-tagged side, other than the electron/positron, is assumed to be a proton. After these requirements have been imposed on the dataset, there is still a sizable contribution from the non-leptonic Λ → pπ^−^ decay. To suppress these events, we assume that the double-tagged semileptonic candidates are in fact the hadronic decay but with a misidentified electron. This modifies the Λ decay vertex, which in turn also modifies the Λ momentum vector. Then a four-constraint kinematic fit (4C-fit) is imposed, which tests energy and momentum conservation in the ${\rm{J}}/{\rm{\psi }}\to \Lambda \bar{\Lambda }$ hypothesis. If the *χ*^2^ of the 4C-fit is lower than 50, then the event is more likely to be a background and hence discarded. The ${\chi }_{4{\rm{C}}}^{2}$ selection removes (84.4 ± 0.2)% and (4.30 ± 0.02)% of the background and signal components, respectively. Except for Λ → pπ^−^, we find no other source of peaking background. A potential background that includes an extra photon, Λ → pπ^−^*γ* decay, was also studied with an exclusive MC simulation. Its contribution was found to be negligible, only 0.02%. To improve the data quality, the electron momentum is required to be larger than 0.1 GeV *c*^−1^. Because the neutrino is undetected, the kinematic variable ${U}_{{\rm{miss}}}\equiv {E}_{{\rm{miss}}}-c|{\vec{p}}_{{\rm{miss}}}|$ is introduced. Here *E*_miss_ and ${\vec{p}}_{{\rm{miss}}}$ are the missing energy and momentum carried by the neutrino, respectively. These are calculated from ${E}_{{\rm{miss}}}={E}_{{\rm{beam}}}-{E}_{{\rm{p}}}-{E}_{{{\rm{e}}}^{-}}$ and ${\vec{p}}_{{\rm{miss}}}={\vec{p}}_{\Lambda }-{\vec{p}}_{{\rm{p}}}-{\vec{p}}_{{{\rm{e}}}^{-}}$, respectively, in which *E*_beam_ is the beam energy, ${E}_{{\rm{p}}({{\rm{e}}}^{-})}$ and ${\vec{p}}_{{\rm{p}}({{\rm{e}}}^{-})}$ are the measured energies and momentum of the proton (electron), respectively. To determine the Λ momentum, ${\vec{p}}_{\Lambda }$, the momentum and magnitude from the produced $\bar{\Lambda }$ partner combined with the known beam energy are used. For the signal candidates, the *U*_miss_ distribution is expected to peak around zero. The four-momentum transfer *q*^2^ is determined as ${q}^{2}={({E}_{{\rm{beam}}}-{E}_{{\rm{p}}})}^{2}-{({\vec{p}}_{\Lambda }-{\vec{p}}_{{\rm{p}}})}^{2}$.

### Double-tagging technique

At BESIII, semileptonic events are produced in the reaction ${{\rm{e}}}^{+}{{\rm{e}}}^{-}\to$ ${\rm{J}}/{\rm{\psi }}\to \Lambda \bar{\Lambda }$. For each event, we first reconstruct the decay $\bar{\Lambda }\to \bar{{\rm{p}}}{{\rm{\pi }}}^{+}$ (or Λ → pπ^−^), which defines the single-tag sample. The semileptonic decay is then searched for in the system recoiling against the single-tag candidate; events in which it is identified from the double-tag sample. The single-tag and double-tag event yields are given by

5 $${N}_{{\rm{ST}}}=2{N}_{\Lambda \bar{\Lambda }}{{\mathcal{B}}}_{{\rm{ST}}}{{\epsilon }}_{{\rm{ST}}},$$

and

6 $${N}_{{\rm{DT}}}=2{N}_{\Lambda \bar{\Lambda }}{{\mathcal{B}}}_{{\rm{ST}}}{\mathcal{B}}(\Lambda \to {{\rm{pe}}}^{-}{\bar{\nu }}_{{\rm{e}}}){{\epsilon }}_{{\rm{DT}}},$$

in which ${N}_{\Lambda \bar{\Lambda }}$ is the number of produced $\Lambda \bar{\Lambda }$ pairs, ${{\mathcal{B}}}_{{\rm{ST}}}$ and ${\mathcal{B}}(\Lambda \to {{\rm{pe}}}^{-}{\bar{\nu }}_{{\rm{e}}})$ are the corresponding branching fractions and *ϵ*_ST(DT)_ is the ST(DT) reconstruction efficiency. The common factor $2{N}_{\Lambda \bar{\Lambda }}{{\mathcal{B}}}_{{\rm{ST}}}$ cancels in the ratio, allowing the absolute branching fraction to be determined directly from the measured yields and efficiencies,

7 $${\mathcal{B}}(\Lambda \to {{\rm{pe}}}^{-}{\bar{\nu }}_{{\rm{e}}})=\frac{{N}_{{\rm{DT}}}}{{{\epsilon }}_{{\rm{DT}}}}\frac{{{\epsilon }}_{{\rm{ST}}}}{{N}_{{\rm{ST}}}}.$$

### Definition of the helicity amplitudes

In the ${{\rm{e}}}^{+}{{\rm{e}}}^{-}\to \Lambda (\to {{\rm{pe}}}^{-}{\bar{\nu }}_{{\rm{e}}})\bar{\Lambda }(\to \bar{{\rm{p}}}{{\rm{\pi }}}^{+})$ process, the ‘master coordinate system’, denoted ${\mathcal{R}}$, is defined in the e^+^e^−^ c.m. system. In this system, we define the unit vector $\hat{\vec{z}}$ in the direction of the positron momentum. The coordinate system ${{\mathcal{R}}}_{\Lambda }$ is then defined in the rest frame of the Λ baryon, with the *z*-axis along the unit vector ${\hat{\vec{z}}}_{\Lambda }$ defined by the direction of the Λ momentum in the ${\mathcal{R}}$ system. A Cartesian coordinate system with ${\hat{\vec{x}}}_{\Lambda }$ and ${\hat{\vec{y}}}_{\Lambda }$ unit vectors is defined as

8 $${\hat{\vec{x}}}_{\Lambda }=\frac{\hat{\vec{z}}\times {\hat{\vec{z}}}_{\Lambda }}{|\hat{\vec{z}}\times {\hat{\vec{z}}}_{\Lambda }|}\times {\hat{\vec{z}}}_{\Lambda },\,{\hat{\vec{y}}}_{\Lambda }=\frac{\hat{\vec{z}}\times {\hat{\vec{z}}}_{\Lambda }}{|\hat{\vec{z}}\times {\hat{\vec{z}}}_{\Lambda }|}.$$

The helicity system ${{\mathcal{R}}}_{\bar{\Lambda }}$ is defined in the same way in the $\bar{\Lambda }$ rest frame and because ${\hat{\vec{z}}}_{\Lambda }=-{\hat{\vec{z}}}_{\bar{\Lambda }}$.

### The maximum log-likelihood fit procedure

A simultaneous fit is performed to the two *c*.*c*. channels, ${\rm{J}}/{\rm{\psi }}\to$ $\Lambda (\to {{\rm{pe}}}^{-}{\bar{\nu }}_{{\rm{e}}})\bar{\Lambda }(\to \bar{{\rm{p}}}{{\rm{\pi }}}^{+})$ and ${\rm{J}}/{\rm{\psi }}\to \Lambda (\to {\rm{p}}{{\rm{\pi }}}^{-})\bar{\Lambda }(\to \bar{{\rm{p}}}{{\rm{e}}}^{+}{\nu }_{{\rm{e}}})$. At this level of precision charge parity is a conserved symmetry, so these charge-parity-odd relations are valid: *α*_−_ = −*α*_+_, ${g}_{{\rm{av}}}^{-}=-{g}_{{\rm{av}}}^{+}$, ${g}_{{\rm{w}}}^{-}={g}_{{\rm{w}}}^{+}$ and ${g}_{{\rm{av}}2}^{-}=-{g}_{{\rm{av}}2}^{+}$, which reduces the number of free parameters in **ω**. For *N* experimental events, the likelihood, constructed from the probability density function for an event characterized by **ξ**_*i*_ is

9 $${\mathcal{L}}=\mathop{\prod }\limits_{i=1}^{N}{\mathcal{P}}({{\boldsymbol{\xi }}}_{i};\omega )=\mathop{\prod }\limits_{i=1}^{N}\frac{{\mathcal{W}}({{\boldsymbol{\xi }}}_{i};\omega ){\epsilon }({{\boldsymbol{\xi }}}_{i})}{{\mathcal{N}}(\omega )},$$

in which *ε*(**ξ**_*i*_) is the detection efficiency, the normalization factor ${\mathcal{N}}(\omega )=\int {\mathcal{W}}(\xi ;\omega ){\epsilon }(\xi ){\rm{d}}\xi$ with weights ${\mathcal{W}}(\xi ;\omega )$, as specified in equation (3). The normalization factor is approximated as ${\mathcal{N}}(\omega )=\frac{1}{M}{\sum }_{j=1}^{M}[{\mathcal{W}}({{\boldsymbol{\xi }}}_{j};\omega )/{\mathcal{W}}({{\boldsymbol{\xi }}}_{j};{\omega }_{\mathrm{gen}})]$ using *M* MC events **ξ**_*j*_ generated using the angular distribution of the full process (equation (3)) with fixed parameters ω_gen_, propagated through the detector and reconstructed in the same way as the experimental data. *M* is chosen to be much larger than *N*; in this case, the ratio is *M*/*N* = 470. To take into account the difference in detection efficiency between MC and data, the detection efficiency is corrected for the final state particles, p, $\bar{{\rm{p}}}$, π^+^, π^−^, e^+^ and e^−^. Applying the same method as described in ref. ^58^, the correction factors are obtained using the control samples ${\rm{J}}/{\rm{\psi }}\to {\rm{p}}\bar{{\rm{p}}}{{\rm{\pi }}}^{+}{{\rm{\pi }}}^{-}$ and e^+^e^−^ → e^+^e^−^*γ*, in which the correlation between the charged particle momentum and its polar angle acceptances is taken into account. Form factors can be expressed in different parametrizations. Our final results are quoted in the standard Weinberg classification, although an equivalent helicity-based representation also exists^59^. In the Weinberg basis, the axial-vector and weak-electricity couplings exhibit strong correlation, *ρ*(*g*_av_, *g*_av2_) ≥ 95%. To reduce this in the fit, we instead use the helicity form factors *g*_+_, *g*_⊥_, *f*_+_ and *f*_⊥_ in the joint angular distribution and subsequently transform them to the Weinberg representation. At zero momentum transfer, this relation is

10 $$\begin{array}{c}{f}_{+}={f}_{1},\,{f}_{\perp }={f}_{1}+(2-\beta ){f}_{2},\\ {g}_{+}={g}_{1},\,{g}_{\perp }={g}_{1}-\beta {g}_{2}.\end{array}$$

This procedure leads to slightly asymmetrical statistical uncertainties for the extracted form-factor ratios (Table 1). For ${g}_{{\rm{w}}}^{-}$, ${g}_{{\rm{w}}}^{+}$ and ⟨*g*_w_⟩ in the first set, this asymmetry is not visible at the quoted precision. For the final |*V*_us_| determination, the effect of these asymmetric uncertainties is negligible; we therefore quote a symmetric uncertainty.

To determine the parameters, the Minuit package from the CERN library is used^60^. The minimizing function is given by $S=-\mathrm{ln}{{\mathcal{L}}}_{\mathrm{data}}+\mathrm{ln}{{\mathcal{L}}}_{\mathrm{bkg}}$, in which ${{\mathcal{L}}}_{{\rm{data}}}$ and ${{\mathcal{L}}}_{{\rm{bkg}}}$ represent the two likelihoods for the semileptonic modes and the background events. The background contribution is evaluated using simulated data. Also, the operational conditions were slightly different for the four data-taking periods (2009, 2012, 2017–2018 and 2018–2019), most notably in the nominal value of the magnetic field. For this reason, the likelihoods are evaluated separately for the four different run periods.

### The |***V***_us_| determination

The partial decay width for $\Lambda \to {{\rm{pe}}}^{-}{\bar{\nu }}_{{\rm{e}}}$ decay, given in equation (1), has been used in the determination of |*V*_us_| using the Cabibbo method^6,7,31^, in which the *q*^2^ dependence of the form factors is not included. To test the systematic effects associated with this approximation, we include the ${\mathcal{O}}({q}^{2})$ terms in the expansion of the relevant form factors about *q*^2^ = 0, following equation (A11) of ref. ^5^:

11 $${f}_{i}({q}^{2})\approx {f}_{i}\cdot \left(1+\frac{\langle {r}_{{f}_{i}}^{2}\rangle }{6}{q}^{2}\right)\,\mathrm{and}\,{g}_{j}({q}^{2})\approx {g}_{j}\cdot \left(1+\frac{\langle {r}_{{g}_{j}}^{2}\rangle }{6}{q}^{2}\right),$$

in which the parameters $\langle {r}_{{f}_{i}}^{2}\rangle$ and $\langle {r}_{{g}_{j}}^{2}\rangle$ are defined consistently for both the dipole parametrization^61,62^ and the so called *z*-expansion used in the lattice QCD calculation^5^ and in the quasipotential approach^63^, although their numerical values differ between these approaches. For the dipole parametrization, these parameters are given by:

12 $$\langle {r}_{{f}_{i}}^{2}\rangle =6\mathop{\sum }\limits_{n=0}^{i}\frac{1}{{m}_{{\rm{V}}}^{2}+n{a}_{{\rm{R}}}^{-1}}\,\,\mathrm{and}\,\,\langle {r}_{{g}_{j}}^{2}\rangle =6\mathop{\sum }\limits_{n=0}^{i}\frac{1}{{m}_{{\rm{A}}}^{2}+n{a}_{{\rm{R}}}^{-1}},$$

with dominant pole masses $${m}_{{\rm{V}}}={m}_{{K}^{\ast }{(892)}^{0}}=0.892\,{\rm{GeV}}$$ and $${m}_{{\rm{A}}}=$$$${m}_{{K}_{1}(1270)}=1.273\,{\rm{GeV}}$$ (ref. ^62^). Here K*(892)^0^ and *K*_1_(1270) are the lowest-lying strange vector mesons with quantum numbers *J*^*P*^ = 1^−^ and 1^+^, respectively. The slope of the Regge trajectory is taken as *α*_R_ = 0.9 GeV^−2^.

The decay width, which takes into account radiative corrections, is given by^44^

13 $${\Gamma }^{c}=\Gamma \cdot (1+{\rm{Cr}}).$$

Here Cr is the radiative correction factor to the decay rate and it has been calculated to be Cr = (1.6 ± 0.5)% for the decay $\Lambda \to {{\rm{pe}}}^{-}{\bar{\nu }}_{{\rm{e}}}$ (ref. ^44^).

We determine |*V*_us_| in two ways. In the first approach, equation (1) is evaluated using our measured form factors ⟨*g*_av_⟩ and ⟨*g*_w_⟩, while fixing *f*_1_ and *g*_av2_ to their SU(3)-conserving values, $-\sqrt{3}/2$ and 0, respectively. Although the universal, nuclear-structure-independent electroweak radiative correction has been extensively studied in refs. ^64–71^, such studies for hyperon decays are very limited. The electroweak radiative correction is incorporated through the prescription ${G}_{{\rm{F}}}^{2}\equiv {G}_{{\rm{F}}}^{2}\cdot (1+{\Delta }_{{\rm{R}}}^{{\rm{V}}})={G}_{{\rm{F}}}^{2}\cdot (1+0.0191)$, following refs. ^64–66^, replacing the nucleon contribution to ${\Delta }_{{\rm{R}}}^{{\rm{V}}}$ with that of the Λ hyperon. In the second approach, |*V*_us_| is determined using lattice QCD form factors that include the *q*^2^ dependence^5^. Specifically, |*V*_us_| is extracted using their equation (A20). To remain consistent with the lattice calculation, we apply the corresponding electroweak radiative correction using *G*_F_ ≡ *G*_F_ ⋅ (1 + *C*) ≡ *G*_F_ ⋅ (1 × 0.0105) (refs. ^5,6^).

### Systematic uncertainties of branching fraction determination

Systematic studies have been performed separately for the branching fraction and form factors. The corresponding results are presented in Extended Data Tables 1, 2 and 3. For the branching fraction, the uncertainties arise from the requirement on the number of tracks and Λ vertex fit, the proton and electron detection, the 4C-fit and the estimation of the number of candidates using single-tagging and double-tagging techniques.

1. Requirement on the number of tracks and Λ vertex fit. The control sample of ${\rm{J}}/{\rm{\psi }}\to \Lambda (\to {\rm{p}}{{\rm{\pi }}}^{-})\bar{\Lambda }(\to \bar{{\rm{p}}}{{\rm{\pi }}}^{+})$ is used to study the systematic uncertainty owing to the requirement of four final tracks and the Λ reconstruction through the vertex fit. For the requirement on the number of tracks, the MC efficiency is corrected by multiplying the correction factor obtained from the control sample, whereas the uncertainty from the correction factor is assigned as the systematic uncertainty. The systematic uncertainty owing to the Λ vertex fit is assigned to be the efficiency difference between the data and MC.

2. p and e^−^ detection. To evaluate the systematic uncertainty from the track reconstruction of the protons and electrons, and their corresponding antiparticles, we use the two control channels ${\rm{J}}/{\rm{\psi }}\to {\rm{p}}\bar{{\rm{p}}}{{\rm{\pi }}}^{-}{{\rm{\pi }}}^{+}$ and e^+^e^−^ → e^+^e^−^*γ*. From these, two-dimensional efficiency plots (cos*θ*_LAB_,*p*_T_), one for each particle species, are obtained. For each track, the efficiency plots are used to obtain a correction factor. For the electron, the correction factor is sizable, that is, (6.81 ± 1.65)%; we therefore take the corrected result as the nominal value and assign the magnitude of the correction as the systematic uncertainty. For the proton, the correction is small, so the uncorrected result is retained as the nominal value and the corresponding correction-induced shift is included in the systematic uncertainty.

3. Kinematic fit. To test the better agreement of kinematic variables between data and MC samples, the simulated sample is corrected using the correction parameters for the charged tracks following the procedure from ref. ^72^. The systematic uncertainty is assigned to be the difference between the fit results with and without the application of correction parameters.

4. Single-tag and double-tag yield extraction. The extracted single-tag and double-tag event yields depend on the fitting functions used to model the kinematically constrained invariant mass and *U*_miss_ spectra. To evaluate the associated systematic uncertainties, we vary the signal and background line shapes in these fits. For the signal component in the *U*_miss_ distribution, the Gaussian convolved with the simulated line shape is removed and only the simulated shape is used, resulting in a 0.10% change in the yield. An analogous procedure is applied to the background: first, the linear component is omitted, leaving only the simulated non-leptonic line shape, and as an extra test, the background is modelled with a third-order Chebyshev polynomial, which produces a larger deviation of 0.79%. The total systematic uncertainty associated with the *U*_miss_ fit is therefore taken to be 0.80%. For the kinematically constrained invariant mass fit, the signal shape uncertainty is estimated by varying the width of the convolved Gaussian, yielding a 0.01% change in the branching fraction. Replacing the background model with a simulated MC shape results in a 0.37% variation. The total systematic uncertainty from this fit is assigned to be 0.37%. To test for potential bias in the fitting procedure, 300 simulated signal-like samples with the same event yields as observed in data are generated and fitted. The resulting fit parameters are consistent with the generated values and no indication of systematic bias is observed.

### Systematic uncertainties of form-factor determination

Systematic uncertainties are assigned on the basis of the electron momentum, parametrization of the *q*^2^ dependence, the estimator and the fit method. We also performed cross-checks on the kinematic fit and decay length but found no notable systematic effect.

1. Consistency check. For the form-factor measurement, 400 pseudo-data samples incorporating the full production and decay dynamics are generated and analysed using the maximum log-likelihood method. The distributions of the fitted parameter values are described by Gaussian functions, one for each form factor. The differences between the Gaussian mean values and the corresponding generated input parameters are assigned as systematic uncertainties.

2. Functional dependence of *q*^2^. The form factors depend on *q*^2^ and our nominal result uses a dipole parametrization. We test two alternative descriptions based on the ‘*z*-expansion’. In the first, the expansion parameters are taken from a relativistic quark model based on a quasipotential approach with a QCD-motivated potential^63^. In the second, they are taken from the lattice QCD calculation of ref. ^5^. The larger deviation from the nominal result is assigned as the systematic uncertainty.

3. Fit method. The fit method introduces a systematic uncertainty from three different sources: fixed value of production and *α*_+_(*α*_−_), parameters, number of background candidates from Λ → pπ^−^ and other sources, and MC efficiency correction factors. The contribution owing to the uncertainty from these three sources is obtained by repeating the fit procedure 100 times after randomly varying all parameters within their uncertainties. A Gaussian function is used to fit each form factor and its width is taken as the systematic uncertainty.

4. e^+^ and e^−^ reconstruction. To study the performance of electron and positron reconstruction, we vary the momentum selection criteria for the e^±^ candidates. The stability of the result is first tested by scanning a ±25 MeV *c*^−1^ window around the nominal value of 100 MeV *c*^−^^1^. Given the momentum resolution of approximately 2 MeV *c*^−^^1^, the requirement is then tightened to a ±5 MeV *c*^−^^1^ window. The systematic uncertainty is assigned as the largest deviation from the nominal result.

## Online content

Any methods, additional references, Nature Portfolio reporting summaries, source data, extended data, supplementary information, acknowledgements, peer review information; details of author contributions and competing interests; and statements of data and code availability are available at https://doi.org/10.1038/s41586-026-10818-8.

## Supplementary information

Peer Review File

## Figures and Tables

**Extended Data Table 1:**
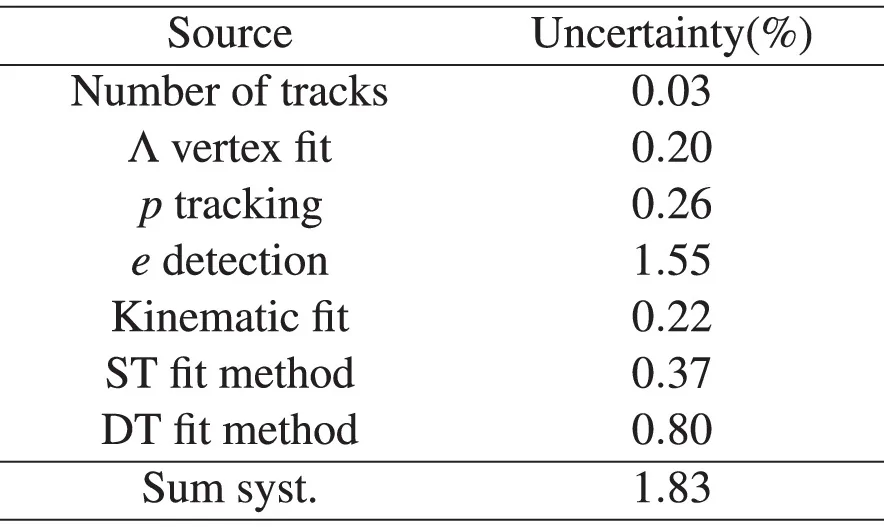
Systematic uncertainty for the branching fraction measurement, relative to the central value, and the sum in quadrature

**Extended Data Table 2:**
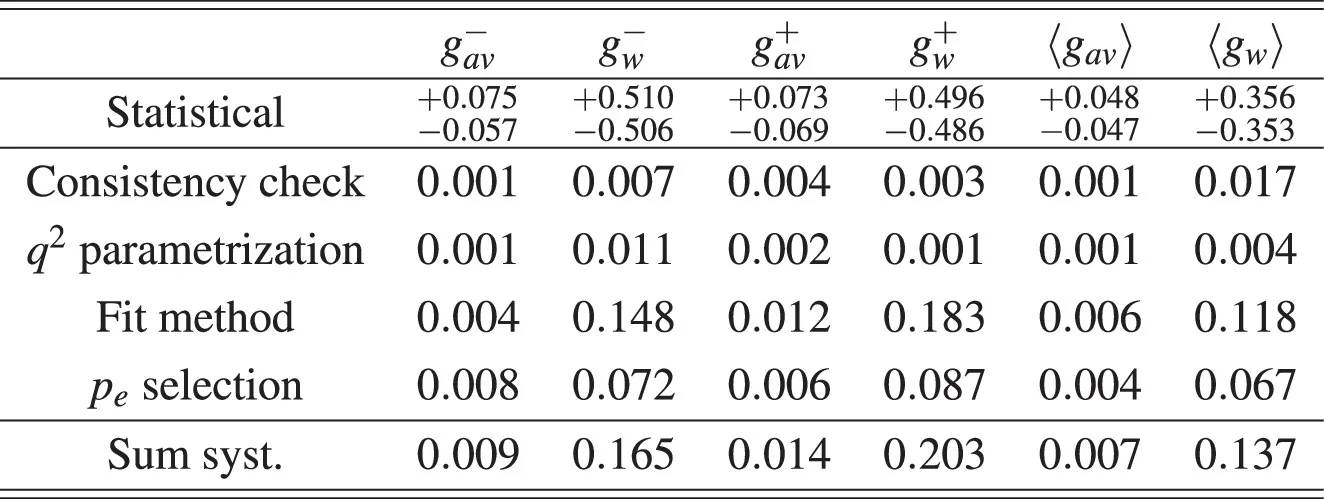
Absolute systematic uncertainties contribution to the first set of form-factor ratios and the final sum, in which the individual contributions are taken in quadrature

The first row shows the statistical uncertainty for reference.

**Extended Data Table 3:**
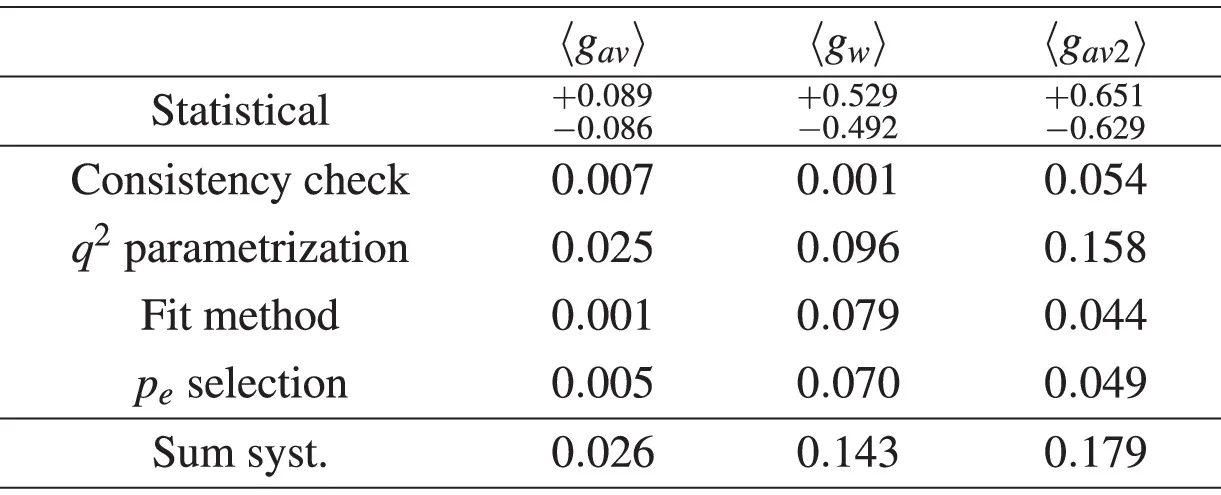
Absolute systematic uncertainties contribution to the second set of form-factor ratios and the final sum, in which the individual contributions are taken in quadrature

The first row shows the statistical uncertainty for reference.

## Data Availability

The data points shown in the plots in this paper are available on request to besiii-publications@ihep.ac.cn.

## Code availability

Software and code that are associated with this publication and publicly available are referenced within the publication content. Specific analysis software or code used to produce the results shown in the publication is preserved within the BESIII Collaboration internally and can be provided on reasonable request, provided it does not contain information that can be associated with unpublished results.
