# Supplementary material for: Exploring baryon semileptonic decays through polarization and entanglement
Source: Nature. 2026 Sep 2;657(8130):92–7. doi: 10.1038/s41586-026-10818-8 (PMC13538038; doi:10.1038/s41586-026-10818-8)
Supplement: Supplementary file 1 [file 41586_2026_10818_MOESM1_ESM.pdf]

nature portfolio

Peer Review File

Exploring baryon semi-leptonic decays through polarization and entanglement

Corresponding Author: Dr Tao Luo

Any redactions in this file are there to maintain patient confidentiality, the confidentiality of unpublished data, or to remove third-party material.

This file contains all reviewer reports in order by version, followed by all author rebuttals in order by version.

Version 0:

Reviewer comments:

Referee #1

(Remarks to the Author)
This manuscript reports the measurement of the structure of hyperons and of the element Vus of the CKM matrix. Both are extremely relevant in the understanding of the structure of matter and of the standard model of particle physics, thus granting immediate interest in the physics community at large but also to other curious readers that may want to learn from a novel experimental technique that is employed to measure fundamental physical quantities.

Semi-electronics decays of hyperons are used, from datasets collected by the BESIII experiment. The novelty of the work stems from the usage of quantum-correlated hyperon-antihyperon pairs, which enables good precision measurements of the above quantities by using semileptonic decays. A significant step forward with respect to existing measurements on semileptonic decays has been achieved, thereby enabling a comparison with similar measurements made with other techniques and with theoretical calculations of ever-increasing precision.

The presented methodology is solid, building from techniques that have been consolidated from previous work. They are reported in reasonable detail in the manuscript; some improvements are necessary to further clarify the methodology and motivate some choices e.g. in the determination of systematic uncertainties and in the choice of the form factor parameterisation (see detailed comments below).

The conclusions are reliable; some improvements and requests for clarifications, detailed below, will help the reader to fully understand the work performed and increase their confidence in the validity of the results. The manuscript references previous literature appropriately. Some suggestions are made below to improve clarity and context.

In summary, I recommend this manuscript for publication once the comments below are addressed.

- page 1, footnote: the argument “0” (==q^2) of f_i(0) and g_i(0) is not defined. Suggest to move this footnote after the q^2 variable is introduced.
- page 2, Fig. 1: you need a bar over the electron neutrino sign
- page 2, line 44: it would help the reader if you could gauge the relative magnitudes of the terms in front of the FFs of eqn. (1), i.e. 1-3/2beta ~0.76, 4beta~0.64, etc., so that the approximations that are made later on can be better appreciated.
- page 2, line 54: I’d suggest to anticipate the Ademollo-Gatto theorem and its reference here (it’s currently on line 57)
- page 3, line 91: are you assuming SU(3) symmetry when you extract the product of |Vus| and the dominant form-factor combination?
- page 4, figure 2 and related text: there is a mismatch between the definition of \theta in fig.2 and lines 97 and 100 and \theta_\Lambda in the caption of Fig. 2, please be consistent
- page 4, line 105: the “seven dimensional angular distribution” is actually 6 angles and one four-momentum transfer (q^2). I would drop the word “angular” or write “the seven-dimensional distribution of the kinematic variables”
- page 4, eqn. (2): I would say in the text that W is an event weight that is used in the max-L fit and refer to the Methods section.
- page 4, line 112: not clear what is the role of index “k” in R_\mu\kappa b_\kappa 0, is an implicit sum assumed? Otherwise

do you really need this index?
- page 4, line 116: \alpha_+ is not defined (and also \alpha_-)
- page 5, line 134: you determine the shape of the dominant background from MC samples, I was wondering if you could check it also on real data? Do you have enough statistics?
- page 5, lines 137-138 and page 3 line 153: I am confused on the event yields. You mention a "signal" event yield of 1791 and a "background" of 207 events, but then you quote a "semi-leptonic" event yield of 1854 events. I had assumed "signal" == "semi-leptonic", but it's evidently not the case. Could you please clarify?
- page 7, table 1: the PDG reports "gA/gV FOR Λ→p e−ve = -0.718 +/- 0.015", you report "g_av^- = 0.718 +/- 0.015". Are you using a different convention? Or should the PDG result reported on the following line ("g_av^+") ?
- page 7, table 1: even though you explicitly mention it in the caption, I find a bit confusing that the uncertainties are defined differently in the three parts of that table (the upper part: stat+syst, the middle part: total, the lower part: stat only). Also, you don't report any systematic uncertainties in the lower part, why? Are they negligible?
- page 7, table 1: how are your measurements of the FF ratios comparing with the LQCD results? Would this comparison be possible? It would further strengthen your manuscript.
- page 1, reference 27: this is not a published paper, but only an arXiv preprint; do you know if a publication is planned?
- page 13, line 307: you mentioned that you tune MC to match the production and decay dynamics that you measured in this manuscript. Isn't there a circular argument? Do you follow an iterative procedure?
- page 14, line 441: the symbol csi refers to the kinematic variables, I think you are referring to omega (i.e. the production and decay dynamics ones) here?
- page 15, eqn. 5: this is the likelihood for your signal, you mention later on (line 455) that your minimisation function also includes backgrounds, but you do not say too much about how you parameterise it, if there is any functional dependence on the seven kinematic variables, etc. Could you please give some details? In particular, it would be reassuring to know that the backgrounds do not produce any biases on the measured parameters (FF ratios).
- page 15, line 455: based on the previous comment, I would change L_data into L_signal and rename L in eqn. 5 accordingly
- page 15, eqn. 5: the efficiency depends only on csi (the kinematic variables); did you check/correct for any possible dependence on omega (==the production and decay dynamics variables)?
- page 15, line 461: the section on |Vus| determination also contains considerations on the fit to the form factor ratios, see lines 471 and following regarding the helicity-based definition of form factors. So if I understood correctly, in the end you are performing the likelihood fit in terms of the helicity-based form factor, correct? Could you please clarify? If this is the case, I think this information is important and should be mentioned beforehand.
- page 16, line 507 and following: you assign systematic a systematic uncertainty on the ST fitting function, but I realise you have not mentioned yet how you determine the yield and efficiency of the ST sample. You say at lines 150 and following that you take these numbers from reference 43, perhaps it would be helpful to remind how do you get those numbers (I guess an invariant mass fit to the proton-pion invariant mass distribution?)
- page 17, line 522 and following: it is not clear how you arrive at the conclusion of not correcting for any biasses nor assigning any systematic uncertainty to the parameterisation of the q2 dependence of the form factors. You motivate this choice to the consistency of the default and alternative fits within one standard deviation: is this the statistical standard deviation? Anything else? In any case, I would assign a systematic uncertainty as the difference between the default fit and the one obtained with the z-expansion parameterisation, no?
- page 17, line 535 and following: could you please detail how you determine this systematic uncertainty? You have a selection cut on 0.1GeV/c on the electron/positron momentum, how much do you vary this cut? How do you justify these variations? Btw shouldn't you assign a similar systematic uncertainty on the branching fraction determination?

Referee #2

(Remarks to the Author)
We congratulate the authors on this very important piece of work and read the paper draft with interest. We feel that the text itself could do with considerable improvement and clarity although cannot see any major issues regarding the robustness of the results. The procedure and presentation is at times rather unclear and we give several suggestions for areas in which we feel improvements are necessary.

The authors have delivered a technically sophisticated and carefully executed analysis, which has produced an important and timely measurement. The work clearly reflects substantial effort and expertise, and while we appreciate the authors' ambition to submit the results to Nature, the manuscript in its current form does not sufficiently convey the originality or broader significance of the study. As written, the novelty and impact of the results are not fully evident, which makes the case for publication less compelling than it could be. The headline results presented in Table 1 give uncertainties that are considerably less precise than the current world averages and it's unclear that this measurement makes significant gains on current understanding.

To strengthen the manuscript and more convincingly justify its suitability for Nature, we encourage the authors to elaborate on the aspects of the work that are genuinely novel, conceptually innovative, or uniquely challenging. This may include clarifying how the analysis advances beyond previous measurements, highlighting methodological developments or experimental insights that are introduced here for the first time, and articulating why the result provides qualitatively new understanding rather than an incremental refinement. Additionally, a clearer discussion of the broader implications in terms of theoretical interpretation and future experimental strategy would help to communicate the full significance of the work to a general scientific audience.

Please find below some general comments according to the headings laid out in Nature’s recommendations for reviewers. Below that we provide specific line-by-line comments on areas we found certain difficulties.

A. Summary of the key results

The article presents a measurement of the L -> p e nu branching fraction and differential form factors using data taken at the J/psi resonance (decaying coherently into L Lbar pairs) in e^+ e^- collisions at the BES III experiment. It exploits the well-established double tagging technique and uses a helicity formalism to describe the spin-entangled baryon states, which has been proposed in recent literature, but to our knowledge not exploited for baryon decays in an experiment such as this before. These measurements are then used, along with external theoretical inputs, to produce an extraction of the magnitude of the CKM matrix element, |Vus|.

Both the branching fraction measurement and the extraction of |Vus| are compatible with, but considerably less precise than, the current world averages. The charge averaged values of the form factors with the constraint that gav2=0 are also considerably less precise than the current state-of-the-art. The charge specific form factors are measured for the first time as is, in a different variant of the fit, the gav2 contribution, which is found to be consistent with the SU(3) symmetry expectation of zero.

B. Originality and significance: if not novel, please include reference

As discussed briefly above, it is hard to understand what is truly of originality and significance in the current manuscript. The double tagging technique is well established (MARK-III experiment, Ref[42] of paper) and the helicity formalism is used in several places for amplitude analyses of flavour decays. The form factors are already measured more precisely elsewhere (all-be-it charge averaged). The extraction of |Vus| is less precise than other measurements.

What certainly is new is the measurement of the charge specific form factors although these seem perfectly consistent with being charge symmetric. An improved revision could highlight the new method of spin-entangled polarised baryons and first determination of gav2 showing consistency with SU(3) symmetry.

C. Data & methodology: validity of approach, quality of data, quality of presentation

The collection and quality of the data, as well as the validity of the approach all seem perfectly valid and consistent. We had no issues with this.

The quality of the presentation however is severely lacking and could do with considerable improvement. We have several specific comments on the presentation (both visual and narrative) that we feel could improve the manuscript substantially.

Perhaps one remaining general question is on the use of whether any data-simulation corrections are incorporated to account for residual differences (seems relevant around L397). This seems potentially relevant for particle identification efficiencies (proton / electron).

We also found it a bit unclear how the Umiss fit is then subsequently used for the fit to kinematic variables.

D. Appropriate use of statistics and treatment of uncertainties

The use of statistics and treatment of uncertainties seem reasonable. The description provided in the Methods section (L437) is helpful. We are unsure whether the subsection beforehand on coordinates is really necessary. This coordinate system is not referred to elsewhere as far as we could see.

In L476 there is a mention that the statistical uncertainties are slightly asymmetrical but the end result is not.

For the systematic on p and e detection: why is the method with correction not the nominal method?

Are toy studies performed for the branching fraction fit? It’s not clear if the width is unbiased.

E. Conclusions: robustness, validity, reliability

On the whole the conclusions seem robust and valid and it’s only at this point of the text where we see some justification on the importance of the measurement.

F. Suggested improvements: experiments, data for possible revision

Our suggestions are only for presentation, readability, improvement of clarity and context, and justification of approach / significance of results.

G. References: appropriate credit to previous work?

Yes

H. Clarity and context: lucidity of abstract/summary, appropriateness of abstract, introduction and conclusions

There is a lot of technical jargon and detail that feels inappropriate for a Nature paper. There were also several grammatical issues that we do not highlight specifically and leave to the editorial process:

The title feels too long and yet is still incredibly vague. I am reluctant to come up with an alternative but I think it could be something more concise / attractive?
The abstract feels too long and reads more like an Introduction. I think it could be dramatically simplified. It is also rather unclear as several symbols are used but not properly defined until much later
I do not think that the decay rate being proportional to |Vus|^2 \cdot (f_1^2 + 3g1^2) needs to be written in the abstract. The form factor f_1 does not need to mentioned in the abstract. The formula g_av = g1/f1 nor does g_av2 = g2/f1. One can simply state the results found. It seems unusual that no value (or range of values) for |V_us| are provided in the abstract.
The footnote of the abstract says that f_i = f_i(0) but at this point it's unclear what the 0 would refer to
Fig 1 could do with some improvement. It's unclear what is is really meant to portray. It seems a mixture between a Feynman diagram and a topology diagram. The neutrino should have bar over it and the arrow should point in the opposite direction. The quarks within the hadrons are in the wrong place for a Feyman diagram (the s quark should be near or at the top because it is the one that undergoes the transition. The u and d should be near the bottom because they are the spectators. There should be a vertex where Vus is and Vus should be bigger. It feels a bit off to have the form factors displayed in the way they are. Is this really needed? Why the different colours? I think the diagram would be much better as purely a Feynman diagram without any of the decoration. The form factors are really properties of the hadron but the way the diagram is drawn (with the form factors in the middle) seems to imply that they are properties of the s -> u transition.
In Eq1 you say you are neglected the q^2 dependence but it would be explicit to write that here f1 and g1 are evaluated at q^2=0.
Do f1, f2, f3 (g1, g2, g3) have some physical interpretation. It would perhaps be helpful to explain if so. In particular in the context of what is said in L51 that f_1 in particular depends on SU(3) symmetry. I think it would be useful for a Nature reader if you explained that this SU(3) symmetry assumption is assuming that s and d quarks are equivalent and that the breaking of this symmetry is simply from their mass difference which is very small with respect to both M_\lambda and M_proton.
The definition of F_2 (Eq 6 of the methods) I think should be moved to the text. It could even be written inline but I think is an important piece to keep in.
L47 I think it would be clearer to write axial-vector, weak-magnetic and weak-electric couplings
L50: angular distributions here is very vague. What is meant by this? Is this from experimental determinations of the differential decay rate?
L56: QCD is not defined.
L66: you are talking about one single experiment but then quote an average value from the PDG.
L68: Ademollo-Gatto only protects at leading order right?
L75: you mention second-class interactions with no context. This feels too technical for Nature without giving some context or explanation of what it means. Some of the background should then be given on first-class vs second-class. I guess if removing the f_3 and g_3 then it is only g_2 left as the second-class? Can you provide an explanation of this which is more accessible to a broader audience.
L102: Is the symbol for the reference system really needed?
Fig 2. Now this is a topology like diagram which is very helpful but also has some issues. The electron and positron point outwards not inwards. On the left there should be \varphi_{\bar{p}}. In the Fig 2 caption you say the grey area is the Lambda rest frame but that's only true for part of the grey area (the left most), the right-most grey area is depicted in the Jpsi rest-frame (same comments for the green and yellow). I like the colouring but the description is incorrect. It seems unnecessary to define all of the axes in the caption here because they never seem to be used in the main text.
Fig 3 looks rather untidy. A log scale seems unnecessary, the signal shape seems to have considerable variations in its tails. Including a component of "Other backgrounds" at the 10^-3 seems rather pointless (it's inclusion can surely not have any effect). The background description at high values seems to be quite poor. It's rather unusual to show the pull distribution for these kinds of plots.
L120: "The particles are" -> "The reconstructed particles" or something that makes clear that you don't mean the neutrinos
L130ish. There doesn't seem to be any description of what the background is nor how it is simulated.
L141: The double tagging technique should be explained in methods. I think this could also be made clearer in the text at this point by simply stating that DT means one side is reconstructed as L -> p e nu and the other as L -> p pi and ST means that only one side is reconstructed as L -> p pi.
L156: bit unclear about the second uncertainties for the numbers and the BF. The N and eff all have one uncertainty, is this just statistical. The BF has two, is this statistical and systematic – what are the systematic sources here? Can it be reference to methods?
L158: it's not completely clear how this is interacting with the signal yield fit. Do you select a signal window in Umiss and analyse these events?
L179: is the previous measurement statistically independent?
L227: "uses the normalization based on …" -> this is a bit too unclear
Fig 4: Is this pie chart really necessary, we feel it doesn't add any information and pie charts are often discouraged by the data visualisation community.
L428: Is this subsection really needed. I don't see where the coordinates are ever used so I'm not sure it's necessary to have to define the coordinate systems.
L441: "in xi" -> shouldn't this be omega?
L458: "the four data taking period": these are not defined
L508: fitting functions are used for the ST and DT variables. It's not clear what you mean ST and DT variables. Did you change the PDFs in the Umiss fit? If yes, can you be more explicit in the parametrisation change?

Referee #3

(Remarks to the Author)
This manuscript reports a simultaneous experimental measurement of the $\Lambda\rightarrow pe^{-}\bar{\nu}_{e}$ branching ratio and form factor ratios $g_{a\nu}$, g_{w} at BESIII. Using the results, the authors determined the magnitude of CKM matrix element $|V_{us}|$. This is an important effort which is definitely worth publication. However, I am not fully convinced that it has reached the standard for publication in Nature. My concerns are as follows:

• The result of $\mathcal{B}(\Lambda\rightarrow pe^{-}\bar{\nu}_{e})$ reported in this manuscript is not the most precise determination comparing to existing publications, e.g. Ref.[10]. The authors may argue that their determination is direct, whereas Ref.[10] relied on a relative branching fraction, but it still does not change the conclusion.

• Subsequently, the value of $|V_{us}|$ reported in this manuscript is also not the most precise one. Not just that it is 5 times less precise than the average result from kaon decays, it is also less precise than those quoted in Refs.[9,34] which were obtained similarly from hyperon decays.

The reliability of the quoted theory uncertainty is also a concern; in particular, I argue that the study of radiative corrections in this manuscript is incomplete. The authors utilized the results in Ref.[47], but that paper only studied the so-called ``model-independent'' part of the radiative correction. They did not study the ``model-dependent'' piece which, according to the paper, only renormalizes the form factors. However, this piece is crucial if one tries to connect the measured branching ratio to the Standard Model (which is also a ``model'' according to the criteria of Ref.[47]) in order to extract $|V_{us}|$. For example, the universal electroweak logarithm (which is common to all semileptonic decays of hadron, see, e.g. Phys.Rev.Lett.71(1993)3629-3632)

$$S_{\text{EW}}=1+\frac{2\alpha}{\pi}\left(1-\frac{\alpha_{s}}{4\pi}\right)\ln\frac{M_{Z}}{M_{\rho}}+\mathcal{O}\left(\frac{\alpha\alpha_{s}}{\pi^{2}}\right)\approx1.0232$$

corrects f_{1}^{2} from its pure-QCD value by 2%, which is at same level with the experimental uncertainty. Such corrections must be applied. Then, there is the non-perturbative part of the radiative correction to f_{1} which must be accounted for in the error budget. This is a standard practice, for example, in the determination of $|V_{ud}|$ from nuclear, neutron or pion decay, as well as the determination of $|V_{us}|$ from kaon decays which, I believe, should also be followed by the authors of this manuscript.

I also had a hard time reproducing the numerical results in Table I, $|V_{us}|_{\text{SU(3)}}=0.2199(94)$. This result is said to be obtained from Eq.(1):

$$\mathcal{B}=\frac{\tau_{\Lambda}}{\hbar}G_{F}^{2}|V_{us}|^{2}\frac{\beta^{5}M_{\Lambda}^{2}}{60\pi^{3}}f_{1}^{2}\left[\left(1-\frac{3}{2}\beta\right)(1+3g_{av}^{2})+\frac{2}{7}\beta^{2}\left(3+3g_{w}+2g_{w}^{2}+6g_{av}^{2}\right)\right]$$

where $\beta\equiv(M_{\Lambda}-M_{p})/M_{\Lambda}$, and I have set $g_{2}=0$ following the treatment in the manuscript. The input I used are (here I only quote the central values, and notice that the error bars of all inputs below are almost symmetric):

$M_{\Lambda} = 1115.683\:\text{MeV}$
$M_{p} = 938.27208816\:\text{MeV}$
$G_{F} = 1.1663788\times10^{-11}\text{ MeV}^{-2}$
$\tau_{\Lambda} = 2.617\times10^{-10}\text{ s}$

together with the experimental results from this paper (obtained with the assumption $g_{2}=0$):

$\mathcal{B}_{c} = 8.16\times10^{-4}$
$g_{av} = 0.729$
$g_{w} = 0.89$

as well as $f_{1}=-\sqrt{3/2}$ in the SU(3) limit. Here, $\mathcal{B}_{c}$ is the actual measured branching ratio which is corrected from the one in Eq.(1) by the ``model-independent'' part of the radiative correction, given in Eq.(10):

$$\mathcal{B}_{c}=\mathcal{B}\cdot\left(1+Cr\right)$$

with Cr=0.016. With all these inputs, I obtain the central value $|V_{us}|_{\text{SU(3)}}=0.2260$ which is different from 0.2199 quoted in the paper.

It is also unclear which lattice inputs from Ref.[27] were used to obtain the second number, $|V_{us}|_{\text{LQCD}}=0.2332(42)$. Is it just the two q^{2}-dependence parameters $\langle r_{f_{1}}^{2}\rangle$ and $\langle r_{g_{1}}^{2}\rangle$, or also include the $q^{2}=0$ couplings f_{1}, f_{2}, g_{1} and g_{2}? Also, notice that Ref.[27] is not yet published, so the reliability of its results is in question.

I am also unable to confirm the consistency of the third result, $|V_{us}|\sqrt{f_{1}^{2}+3g_{1}^{2}}=0.4543(76)$ with the first two. In principle, if I plug in $f_{1}=-\sqrt{3/2}$ and $g_{av}=g_{1}/f_{1}=0.729$, I should get back $|V_{us}|_{\text{SU(3)}}=0.2199$; but I

get 0.2303 instead. Clarifications to all these apparent discrepancies would be useful.

Referee #4

(Remarks to the Author)
I co-reviewed this manuscript with one of the reviewers who provided the listed reports.

Version 1:

Reviewer comments:

Referee #1

(Remarks to the Author)
The revised manuscript addresses my previous remarks and observations. The authors have significantly improved the clarity of the manuscript by reworking paragraphs, adding supplementary material and providing a convincing justification for publishing in Nature. I am fully satisfied by the feedback provided by the authors and recommend publication.

My only comment at this point regards the new title: "Determining baryon semi-leptonic decays...", which I find a bit awkward. I would replace "Determining" by "Investigating" or "An investigation of".

A couple of other minor editorial comments:
- lines 78-79: "which was performed by" --> "by"
- line 90: "...the comparable level" --> "...a comparable level"

Referee #2

(Remarks to the Author)
Many thanks to the authors for their improvements and edits in the revised version. Your response helps to clarify the novel aspects. The added paragraph towards the ends helps to underline these and the additions to the Abstract also make it clearer from the start. There are still many editorial changes I would suggest to improve the readability and language. The second version is better but I'm not sure it is a considerable improvement. Some of these I give further guidance on below but I believe should predominantly lie under the remit of the Nature editorial team.

We are confident the scientific procedures are robust, it is just the description in various places that could do with improvement.

Follow ups:
Data and methodology. The response contained some explanation of how data/MC corrections (particularly with regard to PID) are accounted for using weights. We didn't find too much change to the text. It would be useful to provide some more details on MC corrections in the methods part.

Title still sounds a little odd to me. Is it really determining the decays? I would say it is “Determination of the decay properties”.

Abstract: Still needs some editorial fine tuning. The meaning is sometime ambiguous. For example I think in the first line you mean “the structure has been probed” and then “In order to access the static properties these determinations must be propagated to zero …”

L51: is the quantity under the symmetry precise (is it worth mentioning that the value if -sqrt(3)/2?)

L73 - L75: does the “new experimental value” mean the value determined in this work? This doesn't seem to target g_1 so not sure how it is an important cross validation?

L75: Is the term mirror-asymmetric well defined? Does this mean G-parity asymmetric or purely parity violating?

Eq3: Would be tempted to change the comma to a semi-colon to give a distinction in the pdf between the observables and parameters. I.e. W(\xi; \omega)

Fig 2. Much improved. Would prefer more spacing and less shadow over symbols.

L251: Grammar here. “Determination” twice in quick succession.

L252: What does the “modular” approach really refer to (this was a bit unclear to me - what is modular about it?)

L440: PID is defined as acronym but never used again

L442: Which is the “other track” in this case?

L453: GeV/c2 -> GeV/c

Referee #3

(Remarks to the Author)
The authors have adequately responded to the questions in my first report, and the manuscript is substantially improved. At this stage, I am happy to recommend its publication in Nature. Nonetheless, I invite the authors to consider the following optional suggestions:

1. The authors adopted the model-dependent radiative correction \Delta_R^V from Ref.[67]. That work, however, was explicitly focused on neutron beta decay. The Born term in the expression, for instance, involves the nucleon form factors (axial and isosinglet magnetic Sachs form factor, to be more precise). The appropriateness of applying this value to hyperon decay should be clarified. As a minimal improvement, the authors could at least replace the Born term with the corresponding hyperon contribution.

2. Even for \Delta_R^V alone, several new studies have appeared in recent years, so citing only Ref. [67] (which is based on a hadronic model evaluation) is incomplete. Other relevant evaluations include:

Dispersion relation: Phys.Rev.Lett. 121 (2018) 24, 241804, Phys.Rev.D 104 (2021) 3, 033003
Lattice QCD: Phys.Rev.D 101 (2020) 11, 111301, Phys.Rev.Lett. 132 (2024) 19, 191901
Hadron model: Phys.Rev.D 103 (2021) 11, 113001

I invite the authors to take these evaluations into account when quoting their final result.

Referee #4

(Remarks to the Author)
I co-reviewed this manuscript with one of the reviewers who provided the listed reports.

Open Access This Peer Review File is licensed under a Creative Commons Attribution 4.0 International License, which permits use, sharing, adaptation, distribution and reproduction in any medium or format, as long as you give appropriate credit to the original author(s) and the source, provide a link to the Creative Commons license, and indicate if changes were made.
In cases where reviewers are anonymous, credit should be given to 'Anonymous Referee' and the source.
The images or other third party material in this Peer Review File are included in the article's Creative Commons license, unless indicated otherwise in a credit line to the material. If material is not included in the article's Creative Commons license and your intended use is not permitted by statutory regulation or exceeds the permitted use, you will need to obtain permission directly from the copyright holder.
To view a copy of this license, visit https://creativecommons.org/licenses/by/4.0/

To the referee #1:

We really appreciate your effort in checking the paper. Please find our responses below. Paragraphs commenced with [Response] are the responses.

– –

Referee #1 (Remarks to the Author):

– –

This manuscript reports the measurement of the structure of hyperons and of the element Vus of the CKM matrix. Both are extremely relevant in the understanding of the structure of matter and of the standard model of particle physics, thus granting immediate interest in the physics community at large but also to other curious readers that may want to learn from a novel experimental technique that is employed to measure fundamental physical quantities.

Semi-electronics decays of hyperons are used, from datasets collected by the BESIII experiment. The novelty of the work stems from the usage of quantum-correlated hyperon-antihyperon pairs, which enables good precision measurements of the above quantities by using semileptonic decays. A significant step forward with respect to existing measurements on semileptonic decays has been achieved, thereby enabling a comparison with similar measurements made with other techniques and with theoretical calculations of ever-increasing precision.

The presented methodology is solid, building from techniques that have been consolidated from previous work. They are reported in reasonable detail in the manuscript; some improvements are necessary to further clarify the methodology and motivate some choices e.g. in the determination of systematic uncertainties and in the choice of the form factor parameterisation (see detailed comments below).

The conclusions are reliable; some improvements and requests for clarifications, detailed below, will help the reader to fully understand the work performed and increase their confidence in the validity of the results. The manuscript references previous literature appropriately. Some suggestions are made below to improve clarity and context.

In summary, I recommend this manuscript for publication once the comments below are addressed.

[Response]: Thank you for the recommendation to publish the paper. We have followed up on your comments and suggestions as follows.

- page 1, footnote: the argument "0" (== q^2) of $f_i(0)$ and $g_i(0)$ is not defined. Suggest to move this footnote after the q^2 variable is introduced.

[Response]: It has been moved to page 2 as a footnote, where the q^2 variable is introduced.

- page 2, Fig. 1: you need a bar over the electron neutrino sign

[Response]: It has been corrected.

- page 2, line 44: it would help the reader if you could gauge the relative magnitudes of the terms in front of the FFs of eqn. (1), i.e. $1 - 3/2\beta \sim 0.76$, $4\beta \sim 0.64$, etc., so that the approximations that are made later on can be better appreciated.

[Response]: We have included $\beta = (M_\Lambda - M_p)/M_\Lambda \approx 0.159$.

- page 2, line 54: I'd suggest to anticipate the Ademollo-Gatto theorem and its reference here (it's currently on line 57)

[Response]: Following your suggestion, the reference is now cited earlier in the manuscript (line 54).

- page 3, line 91: are you assuming SU(3) symmetry when you extract the product of $|V_{us}|$ and the dominant form-factor combination?

[Response]: Yes, we do. When calculating $|V_{us}| \cdot \sqrt{f_1^2 + 3g_1^2}$, we start from Eq.(1) and perform two simplifications: setting $g_2 = 0$ and neglecting the contribution of the $\mathcal{F}_2$ term. The first assumes the SU(3)-symmetry limit, while the second is suppressed by the factor, $\frac{2}{7}\beta^2 \approx 0.007$. With these simplifications, our formula becomes identical to Eq. (A19) in the lattice QCD work [2]. Hence, we have applied the same corrections to both the decay width and the weak decay constant constant as were used in the calculation of $|V_{us}|_{\mathrm{LQCD}}$. In order to clarify it, we have added the following sentences to the relevant section of the manuscript on lines 230-239:

"The product of $|V_{us}|$ with the dominant form factor contribution at zero momentum transfer, $q^2 = 0$, is extracted as $|V_{us}| \cdot \sqrt{f_1^2 + 3g_1^2} = 0.4496 \pm 0.0061_{\mathrm{BESIII\ BF_{stat}}} \pm 0.0041_{\mathrm{BESIII\ BF_{syst}}} \pm 0.0009_{\tau_\Lambda} \pm 0.0011_{\mathrm{RC}}$. The reason is that after assuming $g_2 = 0$ and neglecting the $\mathcal{F}_2$ contribution, as the kinematic pre-factor is small, $\frac{2}{7}\beta^2 \approx 0.007$, the partial decay width Γ is approximately proportional to $V_{us}^2(f_1^2 + 3g_1^2)$. Under these two assumptions, the short-handed formula

becomes identical to the the standard next-to-leading order form in the LQCD framework [2]. As a technical side note, the radiative corrections applied to the weak decay constant differ between $|V_{us}|_{\mathrm{SU(3)}}$ and $|V_{us}|_{\mathrm{LQCD}}$. Consequently, when applying radiative corrections to the $|V_{us}| \cdot \sqrt{f_1^2 + 3g_1^2}$, we adopt the same correction method as used for $|V_{us}|_{\mathrm{LQCD}}$."

- page 4, figure 2 and related text: there is a mismatch between the definition of θ in fig.2 and lines 97 and 100 and θ_Λ in the caption of Fig. 2, please be consistent

[Response]: Thank you for spotting this. We have now changed all instances of θ_Λ to be θ.

- page 4, line 105: the "seven dimensional angular distribution" is actually 6 angles and one four-momentum transfer (q^2). I would drop the word "angular" or write "the seven-dimensional distribution of the kinematic variables"

[Response]: We corrected the sentence according to your suggestion, see lines 109-110 of the updated manuscript.

- page 4, eqn. (2): I would say in the text that W is an event weight that is used in the max-L fit and refer to the Methods section.

[Response]: As you suggested, we have inserted the following sentence after Eq. (3): "The W denote the weights used in the maximum log-likelihood fit, as defined in Methods, for determining the form factors of interest. "

- page 4, line 112: not clear what is the role of index "k" in $R_{\mu\kappa} b_{\kappa 0}$, is an implicit sum assumed? Otherwise do you really need this index?

[Response]: The index "κ" gives the relation between the rotation matrix, which provides the angular information of the proton, to the semileptonic Λ decay matrix, which includes the decay couplings, the momentum transfer q^2 and the angular information of the electron.

- page 4, line 116: α_+ is not defined (and also α_-)

[Response]: Following your suggestion, we have revised the sentence (lines 119-120) where the red text indicates the added definition:

"while matrix $a^{\bar{\Lambda}}_{\bar{\nu}0}$ describes the transition from the $\bar{\Lambda}$ to the anti-proton in the non-leptonic decay via the asymmetry parameter α_+ (α_- counts for $\Lambda \to p$ transition) [3]."

- page 5, line 134: you determine the shape of the dominant background from MC samples, I was wondering if you could check it also on real data? Do you have enough statistics?

[Response]: The fully hadronic decay mode $J/\psi \to \Lambda(\to p\pi)\bar{\Lambda}(\to \bar{p}\pi^+)$ was studied by previous BESIII publications [3, 4]. Those results are from the same data taking period and provide much larger yields. It was based on those results that the dedicated generator was developed. Those studies show that the simulated $\Lambda \to p\pi^-$ is in good agreement with data. Therefore we directly use the developed generator from those analyses [3, 4] and employ the simulated shape to describe the contribution from the non-leptonic decay of Λ. The data-driven construction of the MC simulation model has already been mentioned in the revised manuscript, for example in lines 428–431 of the updated draft.

- page 5, lines 137-138 and page 3 line 153: I am confused on the event yields. You mention a "signal" event yield of 1791 and a "background" of 207 events, but then you quote a "semi-leptonic" event yield of 1854 events. I had assumed "signal" == "semi-leptonic", but it's evidently not the case. Could you please clarify?

[Response]: The 1854±49 semi-leptonic event yield corresponds to the signal yield integrated over $|U_{\rm miss}| < 0.05$ GeV (Fig. 3) and is used for the branching fraction extraction. For the form factor analysis, a tighter cut $|U_{\rm miss}| < 0.02$ GeV is adopted to improve the signal purity, yielding 1791 ± 47 signal events and 207 ± 30 background events. Thus the two yields refer to different $U_{\rm miss}$ selections and serve different purposes.

To clarify it, we have modified the original text in lines 136–138 and added the following text before the paragraph that starts with the description of the form factor measurement (line 164):

"Whereas the branching fraction determination requires only yields and efficiencies, the extraction of the form factors requires a detailed understanding of the decay kinematics. To increase the sample purity, we retain only events with $|U_{\rm miss}| < 0.02$ GeV, resulting in 1791 ± 47 signal and 207 ± 30 background events. "

- page 7, table 1: the PDG reports "gA/gV FOR $\Lambda \to pe^-\bar{\nu}_e = -0.718+/-0.015$", you report "$g_{av}^- = 0.718 + / - 0.015$". Are you using a different convention? Or should the PDG result reported on the following line ("g_{av}^+")?

[Response]: The sign difference arises from the choice between two equivalent conventions used in the PDG and introduced by Cabibbo. For this analysis, we adopt the Cabibbo conven-

Table 1: Comparison of the signs of axial-vector coupling within PDG and Cabibbo conventions.

$g_{av}(0)$	Last analysis result	PDG2024 [9]	Cabibbo [1]
$n \to pe^-\bar{\nu}_e$	$-$ [10]	$-$	$+$
$\Lambda \to pe^-\bar{\nu}_e$	$+$ [11]	$-$	$+$
$\Sigma^- \to ne^-\bar{\nu}_e$	$-$ [12]	$+$	$-$
$\Xi^- \to \Lambda e^-\bar{\nu}_e$	$+$ [13]	$-$	$+$

tion. The detailed difference between conventions is provided below.

The V-A (vector minus axial-vector currents) convention is used by PDG [5]: $\langle B_f|\gamma_\lambda(g_V - g_A\gamma_5)|B_i\rangle$, where $B_{i(f)}$ represents a spinor of the initial(final) baryon. The g_A/g_V is defined as $|g_A/g_V|e^{i\phi}$, where ϕ is $0 + n\pi$ if time reversal holds [6]. For all semi-leptonic baryon decays, except for the neutron, experiments assume either that ϕ is either 0 or π, hence only measuring the magnitude and sign of g_A/g_V. For the neutron experiments for semi-leptonic decay of neutron are very sensitive for $\phi = (180.017 \pm 0.026)^o$ [7]. Thus, the normalization over g_A for semi-leptonic neutron decay is applied to all semi-leptonic hyperon decays in PDG. It is also mentioned on PDG pages for g_A/g_V value for $\Lambda \to pe^-\bar{\nu}_e$: "Where necessary, signs have been changed to agree with our conventions, which are given in the “Note on Baryon Decay Parameters” in the neutron Listings.”

The V+A (vector plus axial-vector currents) convention is used by Cabibbo [1,8]: $\langle B_f|\gamma_\lambda(F_1^V(q^2)+F_1^A(q^2)\gamma_5)|B_i\rangle$. Both conventions are consistent while in many experimental analyses the Cabibbo convention is applied. In Table 1 we provide a sign of last experimental results for axial-vector coupling for different semi-leptonic decays with convention used in the analysis and what result is written in PDG within exact SU(3) symmetry.

- page 7, table 1: even though you explicitly mention it in the caption, I find a bit confusing that the uncertainties are defined differently in the three parts of that table (the upper part: stat+syst, the middle part: total, the lower part: stat only). Also, you don’t report any systematic uncertainties in the lower part, why? Are they negligible?

[Response]: The individual contributions to the uncertainty of the $|V_{us}|$ and $|V_{us}|\cdot\sqrt{f_1^2+3g_1^2}$ values are discussed in the text, while in the middle part of Table 1 we only provide their total uncertainty to optimize the size of the table. Regarding the systematics in the lower part of the table, we performed additional systematic studies and incorporated them into the updated table.

- page 7, table 1: how are your measurements of the FF ratios comparing with the LQCD results? Would this comparison be possible? It would further strengthen your manuscript.

[Response]: Our determination of the form factor ratios is model-independent, allowing direct comparison with lattice QCD calculations. Although consistent, the experimental results are currently limited by large uncertainties. Future applications of our method to the larger dataset will enable more stringent quantitative tests. The full potential of this approach is realized by combining experimental measurements with lattice QCD inputs, permitting a model-independent extraction of $|V_{us}|$.

- page 1, reference 27: this is not a published paper, but only an arXiv preprint; do you know if a publication is planned?

[Response]: It has now been published in PRL [2]. We have updated the reference in our new manuscript. In addition, the published version of this PRL paper includes slightly different final values of the form factors in comparison to its earlier arXiv preprint, which we used in the calculation of $|V_{us}|$. We have therefore updated our results to align with the values reported in the published article.

- page 13, line 307: you mentioned that you tune MC to match the production and decay dynamics that you measured in this manuscript. Isn't there a circular argument? Do you follow an iterative procedure?

[Response]: Owing to unclear wording in the original text, a misunderstanding arose. Our intention was to state that the parameters used in the analysis are consistent with our own measurement results presented in Table 1. Specifically, the values of α_{ψ}, $\Delta\Phi$, α_{Λ}, $\alpha_{\bar{\Lambda}}$, and g_{av} for MC simulation are taken from the latest and most precise experimental measurements [4,11], while the value of g_w is taken from the prediction based on Cabibbo theory [1].

We did not adopt an iterative procedure. Instead, systematic uncertainties were evaluated directly. In this manuscript, weighted factors derived from control samples are used to correct the Monte Carlo simulation. If a correction results in only a small change, that change is assigned as a systematic uncertainty. Otherwise, the corrected result is taken as the nominal value, and the uncertainty in the weighted factor is treated as a systematic uncertainty.

- page 14, line 441: the symbol csi refers to the kinematic variables, I think you are referring to omega (i.e. the production and decay dynamics ones) here?

[Response]: Thank you. We have corrected the text.

- page 15, eqn. 5: this is the likelihood for your signal, you mention later on (line 455) that your minimisation function also includes backgrounds, but you do not say too much about how you parameterise it, if there is any functional dependence on the seven kinematic variables, etc. Could you please give some details? In particular, it would be reassuring to know that the backgrounds do not produce any biases on the measured parameters (FF ratios).

[Response]: The likelihood function minimized in the fit comprises two terms, both parametrized by Eq. (9) (revised manuscript). The first term, $\mathcal{L}_{\rm data}$, describes the experimental signal candidates and is normalized using MC–simulated semi-leptonic events. The data sample contains genuine semi-leptonic decays together with a small background from misidentified $\Lambda \to p\pi^-$ decays. This background is explicitly modeled by the second term, $\mathcal{L}_{\rm bkg}$, which is constructed from simulated $\Lambda \to p\pi^-$ events incorporating the full production and decay dynamics as measured by BESIII [3, 4]. The simulated yield is normalized to the background level observed in data.

Potential biases in the fitting procedure are evaluated using an input–output test based on 400 pseudo-experiments, with the observed differences between the fitted and input values assigned as systematic uncertainties. Details are provided at lines 588-592 of the manuscript, in the paragraph beginning with "1. Consistency check."

- page 15, line 455: based on the previous comment, I would change L_{data} into L_{signal} and rename L in eqn. 5 accordingly

[Response]: Consistent with our earlier clarification, we would like to keep the current text.

- page 15, eqn. 5: the efficiency depends only on csi (the kinematic variables); did you check/correct for any possible dependence on omega (==the production and decay dynamics variables)?

[Response]: The treatment of the efficiency in Eq. (9) (revised manuscript) is a part of the method used to extract production- and decay-related parameters. We performed an input-output validation of this method, and the difference between the output and input values were assigned as systematic uncertainties. Details are provided at lines 588-592 of the manuscript, in the paragraph "1. Consistency check.". This approach is consistent with previously published studies, such as Refs. [3, 4]."

- page 15, line 461: the section on $|V_{us}|$ determination also contains considerations on the fit to the form factor ratios, see lines 471 and following regarding the helicity-based definition of form factors. So if I understood correctly, in the end you are performing the likelihood fit in terms of the helicity-based form factor, correct? Could you please clarify? If this is the case, I think this information is important and should be mentioned beforehand.

[Response]: Yes, we used the helicity-based definition of the form factors throughout the fitting procedure. After obtaining the fit results, we converted them into form factors defined according to Weinberg's classification. Following your suggestion, we have moved this description forward so that it now appears in the section "The maximum log-likelihood fit procedure" (lines 499-504).

- page 16, line 507 and following: you assign systematic a systematic uncertainty on the ST fitting function, but I realise you have not mentioned yet how you determine the yield and efficiency of the ST sample. You say at lines 150 and following that you take these numbers from reference 43, perhaps it would be helpful to remind how do you get those numbers (I guess an invariant mass fit to the proton-pion invariant mass distribution?)

[Response]: The Monte Carlo simulation is used to determine the ST efficiency. The ST yield is obtained from a maximum-likelihood fit to the beam-constrained mass distribution of the accepted ST candidates.

Following your suggestion, we have revised the corresponding text (lines 155-157) in the updated draft from

"The values of ST yield and detection efficiency for this sample were previously measured to be $N_{\rm ST} = (14.328 \pm 0.005) \times 10^6$ and $\epsilon_{\rm ST} = (54.09 \pm 0.01)\%$ [14], respectively."

to

"The ST yield and efficiency are from ref [14], where they are obtained from a fit to the invariant mass distribution of the ST candidate distribution. These were found to be $N_{\rm ST} = (14.328 \pm 0.005) \times 10^6$ and $\epsilon_{\rm ST} = (54.09 \pm 0.01)\%$, respectively."

- page 17, line 522 and following: it is not clear how you arrive at the conclusion of not correcting for any biasses nor assigning any systematic uncertainty to the parameterisation of the q2 dependence of the form factors. You motivate this choice to the consistency of the default and alternative fits within one standard deviation: is this the statistical standard deviation? Anything else? In any case, I would assign a systematic uncertainty as the difference between

the default fit and the one obtained with the z-expansion parameterisation, no?

[Response]: Yes, you are correct, and we should assign a corresponding systematic uncertainty. We have compared the results from our nominal fit with those obtained using the z-expansion parameterisation, as well as with those obtained using the parameterisation adopted in the LQCD study [2]. The larger of the resulting differences is taken as the associated systematic uncertainty. The description and result are updated in the manuscript.

- page 17, line 535 and following: could you please detail how you determine this systematic uncertainty? You have a selection cut on 0.1GeV/c on the electron/positron momentum, how much do you vary this cut? How do you justify these variations? Btw shouldn't you assign a similar systematic uncertainty on the branching fraction determination?

[Response]: Systematic effects related to electron and positron reconstruction were investigated by varying the momentum selection requirement for the e^- and e^+ candidates from 75 to 125 MeV/c, with a nominal threshold of 100 MeV/c. The larger difference between the nominal result and those obtained using thresholds of 95 and 105 MeV/c is taken as the associated systematic uncertainty. Given that the momentum resolution is approximately 2 MeV/c in this momentum range, both the range of variation of this cut and the treatment of the systematic uncertainty are sufficiently conservative. In response to your comment, we have replaced the original text with this clarification in the revised manuscript.

For the branching fraction determination, the relevant uncertainty arises from the efficiency of this selection criterion. We therefore used a control sample to correct the electron and positron reconstruction efficiency and assigned the uncertainty in the correction factor as the corresponding systematic uncertainty. The relevant description can be found at lines 553–561, in the paragraph beginning with "2. p and e^- detection."

In contrast, for the extraction of form factors, the corresponding uncertainty involves not only the efficiency of this selection criterion but also the shapes of the distributions of the kinematic variables $\boldsymbol{\xi} = \theta, \theta_p, \varphi_p, \theta_e, \theta_{\bar{p}}, \varphi_{\bar{p}}, q^2$. Consequently, a different treatment was adopted.

References

[1] N. Cabibbo, E. C. Swallow, and R. Winston, Ann. Rev. Nucl. Part. Sci. **53**, 39-75 (2003).

[2] S. Bacchio and A. Konstantinou, Phys. Rev. Lett. **135**, 231901 (2025).

[3] M. Ablikim *et al.* (BESIII Collaboration), Nature Phys. **15**, 631-634 (2019).

[4] M. Ablikim *et al.* (BESIII Collaboration), Phys. Rev. Lett. **129**, 131801 (2022).

[5] M. L. Goldberger and S. B. Treiman, Phys. Rev. **111**, 354-361 (1958).

[6] J. D. Jackson, S. B. Treiman, and H. W. Wyld, Phys. Rev. **106**, 517-521 (1957).

[7] R. L. Workman *et al.* (Particle Data Group), PTEP **2022**, 083C01 (2022).

[8] N. Cabibbo, Phys. Rev. Lett. **10**, 531-533 (1963).

[9] S. Navas *et al.* (Particle Data Group), Phys. Rev. D **110**, 030001 (2024).

[10] F. E. Wietfeldt *et al.*, Phys. Rev. C **110**, 015502 (2024).

[11] J. Dworkin *et al.*, Phys. Rev. D **41**, 780-800 (1990).

[12] S. Y. Hsueh *et al.*, Phys. Rev. D **38**, 2056 (1988).

[13] M. Bourquin *et al.* (Bristol-Geneva-Heidelberg-Orsay-Rutherford-Strasbourg Collaboration), Z. Phys. C **21**, 1 (1983).

[14] M. Ablikim *et al.* (BESIII Collaboration), Phys. Rev. Lett. **127**, 121802 (2021).

To the referee #2:

We really appreciate your effort in checking the paper. Please find our responses below. Paragraphs commenced with [Response] are the responses.

– –

Referee #2 (Remarks to the Author):

– –

We congratulate the authors on this very important piece of work and read the paper draft with interest. We feel that the text itself could do with considerable improvement and clarity although cannot see any major issues regarding the robustness of the results. The procedure and presentation is at times rather unclear and we give several suggestions for areas in which we feel improvements are necessary.

The authors have delivered a technically sophisticated and carefully executed analysis, which has produced an important and timely measurement. The work clearly reflects substantial effort and expertise, and while we appreciate the authors' ambition to submit the results to Nature, the manuscript in its current form does not sufficiently convey the originality or broader significance of the study. As written, the novelty and impact of the results are not fully evident, which makes the case for publication less compelling than it could be. The headline results presented in Table 1 give uncertainties that are considerably less precise than the current world averages and it's unclear that this measurement makes significant gains on current understanding.

To strengthen the manuscript and more convincingly justify its suitability for Nature, we encourage the authors to elaborate on the aspects of the work that are genuinely novel, conceptually innovative, or uniquely challenging. This may include clarifying how the analysis advances beyond previous measurements, highlighting methodological developments or experimental insights that are introduced here for the first time, and articulating why the result provides qualitatively new understanding rather than an incremental refinement. Additionally, a clearer discussion of the broader implications in terms of theoretical interpretation and future experimental strategy would help to communicate the full significance of the work to a general scientific audience.

[Response to General Remarks]: We thank you for this important feedback, which has helped us sharpen the presentation of the core advance of our work.

As you note later, we performed the first measurement of the charge-specific form factors,

even though they appear perfectly consistent with charge symmetry. In addition, we carried out the first determination of g_{av2}, showing consistency with SU(3) symmetry. Both of these "two firsts" explicitly demonstrate that our analysis advances beyond previous measurements.

More importantly, as you noted, the method itself is unequivocally novel, as it marks the first exploitation of polarization and quantum entanglement in baryon semileptonic decays. The revised manuscript has now highlighted this explicitly. The advantage of the approach is quantified through a direct comparison with the Fermilab measurement [1]: although their dataset is approximately twenty times larger than ours, we achieve a comparable precision for g_w, while the uncertainty on g_{av} is only about a factor of 2.5 larger, and, most notably, we determine g_{av2} for the first time, a parameter previously inaccessible. This demonstrates a substantially enhanced single-event sensitivity. This study thus represents a foundational demonstration of the method.

Furthermore, along with the first exploitation of polarization and quantum entanglement in baryon semileptonic decays, this work establishes the foundation for a systematic research program. The same method is already being extended to our following studies of other baryon semileptonic decays at BESIII [2] and is applicable to both present and future particle-antiparticle collider facilities such as the upcoming PANDA experiment [3] and the proposed Super Tau-Charm Factories [4,5]. Because form factors in different semileptonic decays are interrelated [6], results obtained with this method can be combined across channels. Together with continued advances in lattice QCD [7], this program offers a clear path toward a model-independent determination of $|V_{us}|$ with precision competitive with that from kaon decays, thereby providing a critical and independent test of CKM unitarity.

Following your suggestion, we have revised the corresponding discussion in the manuscript as follows:

"The novel modular approach [8] is applied in the analysis marking the first exploitation of polarization and quantum entanglement in baryon semi-leptonic decays. This innovative methodology significantly enhances the single-event sensitivity, as demonstrated by a direct comparison with the Fermilab measurement [1]: although their dataset is approximately twenty times larger than ours, we achieve a comparable precision for g_w, while the uncertainty on g_{av} is only about a factor of 2.5 larger, and, most notably, we determine g_{av2} for the first time, a parameter previously inaccessible. Along with the first exploitation of polarization and quantum entanglement in baryon semileptonic decays, this work establishes the foundation for a systematic research program. The same method is already being extended to our following studies of other baryon semileptonic decays at BESIII [2] and is applicable to both present and

future particle-antiparticle collider facilities such as the upcoming PANDA experiment [3] and the proposed Super Tau-Charm Factories [4, 5]. Because form factors in different semileptonic decays are interrelated [6], results obtained with this method can be combined across channels. Together with continued advances in lattice QCD [7], this program offers a clear path toward a model-independent determination of $|V_{us}|$ with precision competitive with that from kaon decays, thereby providing a critical and independent test of CKM unitarity."

Please find below some general comments according to the headings laid out in Nature's recommendations for reviewers. Below that we provide specific line-by-line comments on areas we found certain difficulties.

A. Summary of the key results

The article presents a measurement of the L $\rightarrow$ p e nu branching fraction and differential form factors using data taken at the J/psi resonance (decaying coherently into L Lbar pairs) in e^+e^- collisions at the BES III experiment. It exploits the well-established double tagging technique and uses a helicity formalism to describe the spin-entangled baryon states, which has been proposed in recent literature, but to our knowledge not exploited for baryon decays in an experiment such as this before. These measurements are then used, along with external theoretical inputs, to produce an extraction of the magnitude of the CKM matrix element, $|V_{us}|$.

Both the branching fraction measurement and the extraction of $|V_{us}|$ are compatible with, but considerably less precise than, the current world averages. The charge averaged values of the form factors with the constraint that gav2=0 are also considerably less precise than the current state-of-the-art. The charge specific form factors are measured for the first time as is, in a different variant of the fit, the gav2 contribution, which is found to be consistent with the SU(3) symmetry expectation of zero.

B. Originality and significance: if not novel, please include reference

As discussed briefly above, it is hard to understand what is truly of originality and significance in the current manuscript. The double tagging technique is well established (MARK-III experiment, Ref[42] of paper) and the helicity formalism is used in several places for amplitude analyses of flavour decays. The form factors are already measured more precisely elsewhere (all-be-it charge averaged). The extraction of $|V_{us}|$ is less precise than other measurements.

What certainly is new is the measurement of the charge specific form factors although these seem perfectly consistent with being charge symmetric. An improved revision could highlight the new method of spin-entangled polarised baryons and first determination of gav2 showing

consistency with SU(3) symmetry.

[Response]: We are grateful for this insightful comment, which has allowed us to better articulate the central novelty of our work. As you have noted, the present precision of the branching fraction and $|V_{us}|$ results is not the central highlight of this study. Instead, our core advance lies in a novel method that enables the first exploitation of polarization and quantum entanglement in baryon semileptonic decays.

The specific advantages of this approach, as well as the substantial impact it can bring to broader and even future studies, as it serves as the foundation of a systematic research program, have already been elaborated in detail earlier in our response. We therefore contend that the novelty and impact of this work lie in pioneering an efficient new experimental paradigm that has the potential to redefine precision studies of baryon semileptonic decays.

Following your suggestion, we have revised the manuscript to highlight the new method of spin-entangled polarized baryons and the first measurement of g_{av2}; the corresponding changes are presented in the revised text (see the red passages above).

C. Data & methodology: validity of approach, quality of data, quality of presentation

The collection and quality of the data, as well as the validity of the approach all seem perfectly valid and consistent. We had no issues with this.

The quality of the presentation however is severely lacking and could do with considerable improvement. We have several specific comments on the presentation (both visual and narrative) that we feel could improve the manuscript substantially.

Perhaps one remaining general question is on the use of whether any data-simulation corrections are incorporated to account for residual differences (seems relevant around L397). This seems potentially relevant for particle identification efficiencies (proton / electron).

[Response]: Yes, we use weight factors derived from control samples to correct the Monte Carlo simulation. These corrections account not only for the tracking and particle identification efficiencies of protons and electrons, but also for the requirements on the number of reconstructed tracks, the Λ vertex fit, and the kinematic fit. If a correction results in only a small change in the results, that change is assigned as a systematic uncertainty. Otherwise, the corrected results are taken as the nominal values, and the uncertainty in the corresponding weight factor is treated as a systematic uncertainty.

We also found it a bit unclear how the Umiss fit is then subsequently used for the fit to

kinematic variables.

[Response]: When fitting the kinematic variables, the parameters are determined by minimizing $S = -\ln \mathcal{L}_{\rm data} + \ln \mathcal{L}_{\rm bkg}$. The likelihood function is defined in Eq. (9) as $\mathcal{L} = \prod_{i=1}^{N} \mathcal{P}(\boldsymbol{\xi}_i; \boldsymbol{\omega}) = \prod_{i=1}^{N} \frac{\mathcal{W}(\boldsymbol{\xi}_i;\boldsymbol{\omega})\epsilon(\boldsymbol{\xi}_i)}{\mathcal{N}(\boldsymbol{\omega})}$. The value of N associated with the background component is determined from a fit to the $U_{\rm miss}$ distribution.

D. Appropriate use of statistics and treatment of uncertainties

The use of statistics and treatment of uncertainties seem reasonable. The description provided in the Methods section (L437) is helpful. We are unsure whether the subsection beforehand on coordinates is really necessary. This coordinate system is not referred to elsewhere as far as we could see.

[Response]: This section is directly related to the discussion surrounding Eq. (3). To make this connection explicit, we have added the sentence "More details of the coordinate system are given in the Methods" at the end of that paragraph. In addition, during the internal review within the BESIII collaboration, we found that the use of an inconsistent coordinate system can lead to incorrect results. For this reason, we believe it is important to retain the relevant passage in the Methods section.

In L476 there is a mention that the statistical uncertainties are slightly asymmetrical but the end result is not.

[Response]: For the form factor ratios, the statistical uncertainties are asymmetric, as reported in Table 1. In particular, for g_w^-, g_w^+, and $\langle g_w \rangle$ in the first set of results, the asymmetry is not visible at the quoted numerical precision. For the final determination of $|V_{us}|$, the asymmetric uncertainties of the form factor ratios have a negligible impact at the current level of precision. We therefore quote a symmetric uncertainty for the $|V_{us}|$ result. To clarify this point, we have added the following text (lines 506-508) to the revised manuscript:

" For g_w^-, g_w^+, and $\langle g_w \rangle$ in the first set, this asymmetry is not visible at the quoted precision. For the final $|V_{us}|$ determination, the effect of these asymmetric uncertainties is negligible; we therefore quote a symmetric uncertainty."

For the systematic on p and e detection: why is the method with correction not the nominal method?

[Response]: For electron detection, we use the corrected result as the nominal value and

assign the uncertainty in the correction factor as a systematic uncertainty. For proton detection, since the correction has only a minor impact, we retain the uncorrected result as the nominal value and treat the effect of the correction as a systematic uncertainty. To avoid confusion, we have revised the corresponding text (lines 557-561) in the updated manuscript from

"For the proton, the difference between the nominal and track-corrected branching fraction is assigned to be the systematic uncertainty. For the electron, the uncertainty associated with the correction factor is assigned as the systematic uncertainty."

to

"For the electron, the correction factor is sizable, i. e. $(6.81 \pm 1.65)\%$; we therefore take the corrected result as the nominal value and assign the magnitude of the correction as the systematic uncertainty. For the proton, the correction is small, so the uncorrected result is retained as the nominal value, and the corresponding correction-induced shift is included in the systematic uncertainty."

Are toy studies performed for the branching fraction fit? It's not clear if the width is unbiased.

[Response]: Based on 300 input–output tests using exclusive Monte Carlo samples, we validated the branching fraction measurement and found no evidence of bias. This information has been incorporated into the updated manuscript at lines 579-582.

E. Conclusions: robustness, validity, reliability

On the whole the conclusions seem robust and valid and it's only at this point of the text where we see some justification on the importance of the measurement.

F. Suggested improvements: experiments, data for possible revision

Our suggestions are only for presentation, readability, improvement of clarity and context, and justification of approach / significance of results.

G. References: appropriate credit to previous work?

Yes

H. Clarity and context: lucidity of abstract/summary, appropriateness of abstract, introduction

and conclusions

There is a lot of technical jargon and detail that feels inappropriate for a Nature paper. There were also several grammatical issues that we do not highlight specifically and leave to the editorial process:

The title feels too long and yet is still incredibly vague. I am reluctant to come up with an alternative but I think it could be something more concise / attractive?

[Response]: Following your suggestion, we have changed it to be "Determining baryon semi-leptonic decays using polarization and entanglement".

The abstract feels too long and reads more like an Introduction. I think it could be dramatically simplified. It is also rather unclear as several symbols are used but not properly defined until much later

[Response]: Following your suggestion, we have reduced the abstract from 28 to 23 lines. For comparison, the abstract in Ref. [4] (*Nature* **614**, 48–53 (2023)) spans 22 lines, and we therefore consider the current length to be appropriate. In addition, as advised, we have ensured that no symbols other than $|V_{us}|$ appear in the abstract.

I do not think that the decay rate being proportional to $|V_{us}|^2 \cdot (f_1^2 + 3g1^2)$ needs to be written in the abstract. The form factor f_1 does not need to mentioned in the abstract. The formula g_{av} = g1/f1 nor does g_{av2} = g2/f1. One can simply state the results found. It seems unusual that no value (or range of values) for $|V_{us}|$ are provided in the abstract. The footnote of the abstract says that $f_i = f_i(0)$ but at this point it's unclear what the 0 would refer to.

[Response]: Following your suggestion, the symbols, you noted, have been removed, and the numerical value of $|V_{us}|_{\rm LQCD}$ has been included. To improve clarity, the footnote has been moved to the same page as the definition of q^2 and revised to read: "$f_i \equiv f_i(q^2 = 0)$ and $g_i \equiv g_i(q^2 = 0)$."

Fig 1 could do with some improvement. It's unclear what is is really meant to portray. It seems a mixture between a Feynman diagram and a topology diagram. The neutrino should have bar over it and the arrow should point in the opposite direction. The quarks within the hadrons are in the wrong place for a Feyman diagram (the s quark should be near or at the top because it is the one that undergoes the transition. The u and d should be near the bottom because they are the spectators. There should be a vertex where Vus is and Vus should be

bigger. It feels a bit off to have the form factors displayed in the way they are. Is this really needed? Why the different colours? I think the diagram would be much better as purely a Feynman diagram without any of the decoration. The form factors are really properties of the hadron but the way the diagram is drawn (with the form factors in the middle) seems to imply that they are properties of the s → u transition.

[Response]: We thank you for your careful review of the figure. We would like to clarify that the illustration is intended as a schematic representation of the decay process for a broad readership, rather than as a precise Feynman or topology diagram. Nevertheless, we have taken all of your suggestions into account to improve the figure, and the revised version is now presented as Fig. 1.

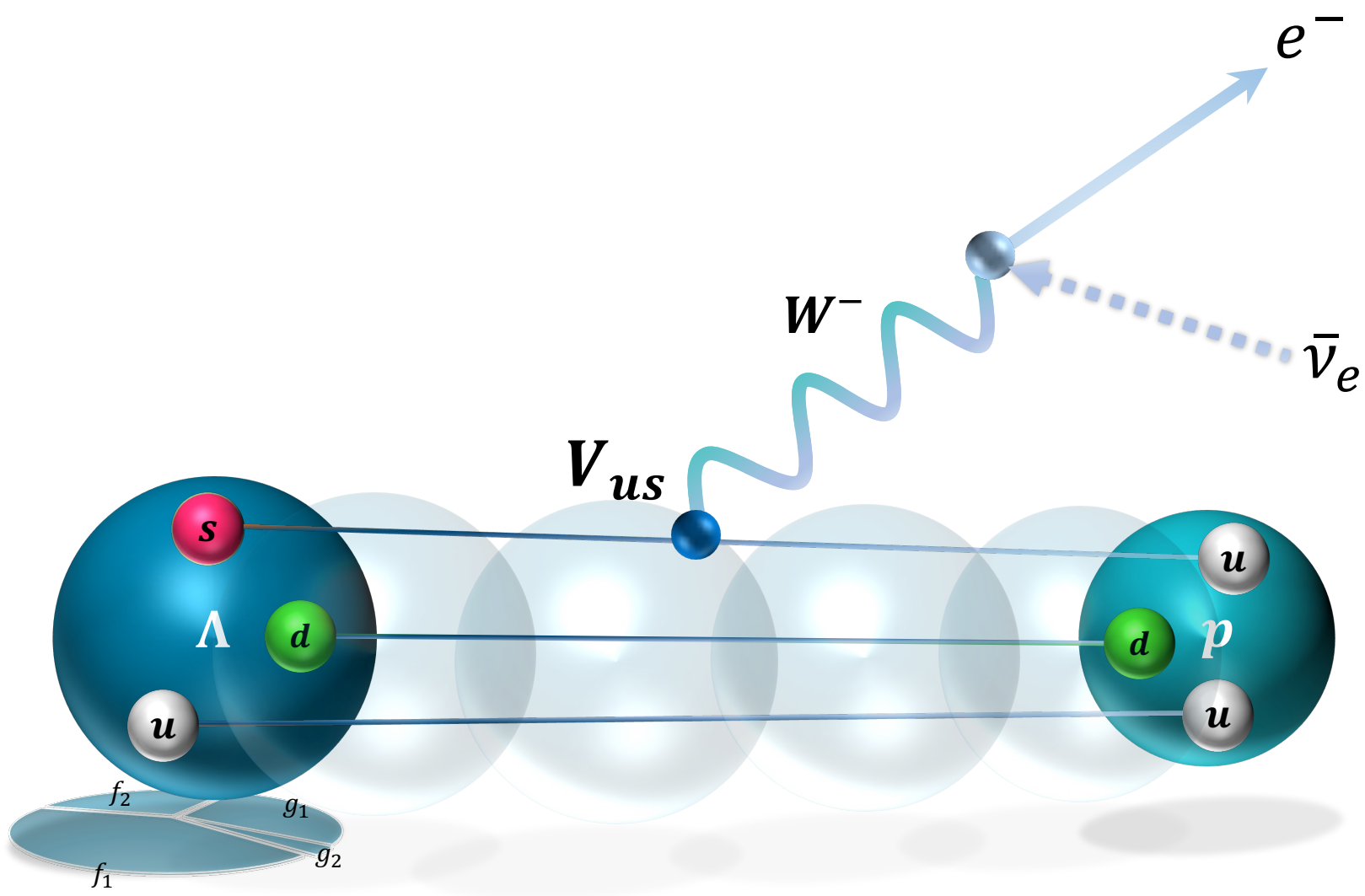

Figure 1: **Schematic representation of the Λ decay to a proton, an electron and an electric anti-neutrino via virtual (mediating) W^- boson. The pie-chart shows the relevant magnitudes of the dominant vector and axial-vector form factors partaking in the transition.**

In Eq1 you say you are neglected the q^2 dependence but it would be explicit to write that here f1 and g1 are evaluated at $q^2 = 0$.

[Response]: The footnote from previous page is moved here to clearly provide that $f_1 \equiv f_1(q^2 = 0)$ and $g_1 \equiv g_1(q^2 = 0)$.

Do f1, f2, f3 (g1, g2, g3) have some physical interpretation. It would perhaps be helpful to explain if so. In particular in the context of what is said in L51 that f_1 in particular depends on

SU(3) symmetry. I think it would be useful for a Nature reader if you explained that this SU(3) symmetry assumption is assuming that s and d quarks are equivalent and that the breaking of this symmetry is simply from their mass difference which is very small with respect to both M_Λ and M_{proton}.

[Response]: The $f_i(q^2)$ and $g_i(q^2)$ $(i = 1, 2, 3)$ represent the vector and axial vector parts of the weak interaction, respectively, and depend on the squared momentum transfer, q^2, of the virtual W boson. The vector form factors are commonly denoted as the vector $f_1(q^2)$, weak magnetism $f_2(q^2)$, and scalar $f_3(q^2)$ form factors. The axial vector form factors are further subdivided into the axial vector $g_1(q^2)$, weak electricity $g_2(q^2)$, and pseudoscalar $g_3(q^2)$ form factors [9]. Each form factor corresponds to a specific current and encodes information about the underlying hadronic structure. Following your suggestion, we have included this description in the revised manuscript, together with a clarification of the SU(3) symmetry assumption.

The definition of F_2 (Eq 6 of the methods) I think should be moved to the text. It could even be written inline but I think is an important piece to keep in.

[Response]: Following your suggestion, we have moved the definition of F_2 to the main text.

L47 I think it would be clearer to write axial-vector, weak-magnetic and weak-electric couplings

[Response]: All of these have been revised in accordance with your suggestion.

L50: angular distributions here is very vague. What is meant by this? Is this from experimental determinations of the differential decay rate?

[Response]: Here, the angular distributions correspond to the kinematic angular variables of the proton and electron in the Λ rest frame, as extracted from the experimental data. In response to your comment, we have replaced the term “angular distributions” with “kinematic angular variables” to improve clarity and precision.

L56: QCD is not defined.

[Response]: The definition is added.

L66: you are talking about one single experiment but then quote an average value from the

PDG.

[Response]: In response to the feedback, we have updated the manuscript to include the Fermilab result while retaining the PDG average, since both sets of values serve as relevant references.

L68: Ademollo-Gatto only protects at leading order right?

[Response]: Yes, Ademollo and Gatto demonstrated that there is no first-order correction to the vector form factor f_1 [10].

L75: you mention second-class interactions with no context. This feels too technical for Nature without giving some context or explanation of what it means. Some of the background should then be given on first-class vs second-class. I guess if removing the f_3 and g_3 then it is only g_2 left as the second-class? Can you provide an explanation of this which is more accessible to a broader audience.

[Response]: The first-class currents - f_1, f_2, g_1, g_3 - are mirror-symmetric transformation of weak hadronic currents while the second-class currents - f_3, g_2 - are mirror-asymmetric. This classification was introduced by Weinberg [11].

The additional description of second-class currents is added in the draft and the corresponding reference is updated.

L102: Is the symbol for the reference system really needed?

[Response]: Thank you for raising this point. We find that retaining the symbol ensures a concise and accurate description throughout the manuscript, and we therefore plan to keep it.

Fig 2. Now this is a topology like diagram which is very helpful but also has some issues. The electron and positron point outwards not inwards. On the left there should be $\varphi_{\bar{p}}$. In the Fig 2 caption you say the grey area is the Lambda rest frame but that's only true for part of the grey area (the left most), the right-most grey area is depicted in the Jpsi rest-frame (same comments for the green and yellow). I like the colouring but the description is incorrect. It seems unnecessary to define all of the axes in the caption here because they never seem to be used in the main text.

[Response]: We have incorporated all of your suggestions to improve the figure, and the

updated version is now presented as Fig. 2. In addition, following your recommendation, all axis descriptions have been removed from the caption.

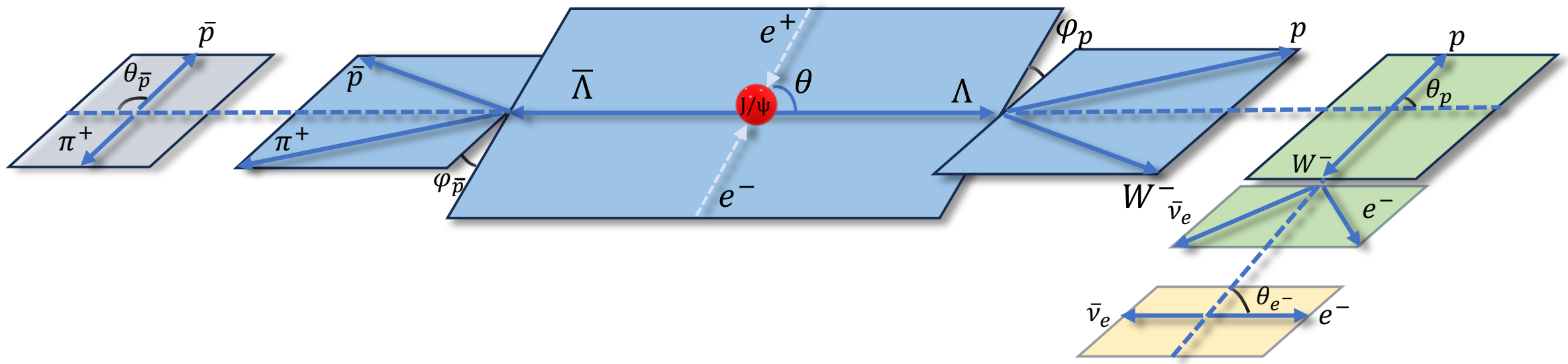

Figure 2: **Definition of the helicity angles for $e^+e^- \to J/\psi \to \Lambda(\to pe^-\bar{\nu}_e)\bar{\Lambda}(\to \bar{p}\pi^+)$.** The angles θ, $\theta_{\bar{p}}$, $\varphi_{\bar{p}}$, θ_p, φ_p, and θ_e are the helicity angles of the Λ, $\bar{p}$, p, and lepton in the e^+e^- c.m. system (blue area), $\bar{\Lambda}$ rest frame (gray area), Λ rest frame (green area), and W^- rest frame (yellow area), respectively.

Fig 3 looks rather untidy. A log scale seems unnecessary, the signal shape seems to have considerable variations in its tails. Including a component of "Other backgrounds" at the 10^-3 seems rather pointless (it's inclusion can surely not have any effect). The background description at high values seems to be quite poor. It's rather unusual to show the pull distribution for these kinds of plots.

[Response]: Regarding figure revisions, we have updated Figure 3 (now presented as Fig. 3) to use a linear scale and have removed the pull distribution.

For the signal description, we employed the MC-derived shape. Although limited statistics in the far tails produce minor shape irregularities, we have verified that generating significantly larger MC samples produces a signal shape whose variations in these regions have a negligible impact on the fit results (on the order of 10^{-1}).

Concerning background contributions, including additional backgrounds in the fit does not change the central value of our result but affects their uncertainty. Studies using inclusive MC indicate that a small level of such backgrounds is expected in the final data sample. The fit yields 0 ± 31 events for these components. Their inclusion is therefore primarily relevant for correctly evaluating the associated uncertainty, which has been incorporated as a systematic effect in our form factor analysis.

Regarding the fit quality in the high-end region, the limited event count leads to large statistical uncertainties. In the current fit, the curve remains within the error bars of the data

points. Given the negligible signal contribution in this region, we consider the fit quality acceptable. As a part of our systematic study on background modeling, we also tested an alternative description using a third-order Chebyshev polynomial, which showed some improvement in the high-end fit. However, the MC-based shape is more physically motivated, and we retain it as our nominal model, assigning the difference from the Chebyshev polynomial fit as a systematic uncertainty.

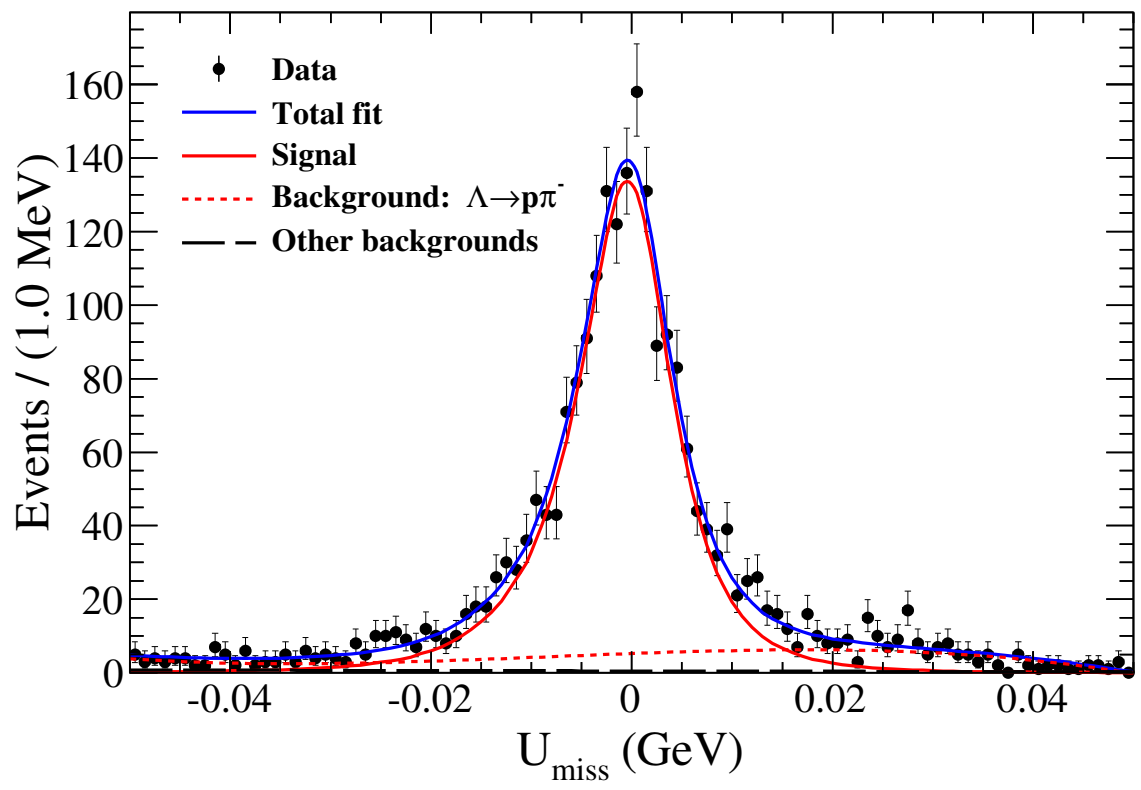

Figure 3: **The $U_{\mathbf{miss}}$ distribution of $\Lambda \to p e^- \bar{\nu}_e$ and $\bar{\Lambda} \to \bar{p} e^+ \nu_e$ candidates**. The black points with error bars represent the observed number of events in each bin, with statistical uncertainties. The blue solid line shows the total fit, which includes the signal candidates (red solid), the dominant background (red dotted line), and the other background contributions (black long-dashed line).

L120: "The particles are" → "The reconstructed particles" or something that makes clear that you don't mean the neutrinos

[Response]: The sentence is modified to take into account that only reconstructed particles can be recorded.

L130ish. There doesn't seem to be any description of what the background is nor how it is simulated.

[Response]: Thank you for raising this point. The description of the background and its simulation is provided in two parts of the manuscript: (1) In the main text (lines 141-143): "The shape of the dominant background contribution, $\Lambda \to p\pi^-$, is obtained using the corresponding MC sample, while the remaining background is described by a linear function." (2) Full simulation details are described in the dedicated "Monte Carlo Simulation" subsection of the Methods.

The main text (line 144) also notes: "More details of the selection and analysis procedure are given in Methods." This subsection provides full details of the event generation, detector simulation, and background modeling for all samples, including the $\Lambda \to p\pi^-$ background.

L141: The double tagging technique should be explained in methods. I think this could also be made clearer in the text at this point by simply stating that DT means one side is reconstructed as L $\to$ p e nu and the other as L $\to$ p pi and ST means that only one side is reconstructed as L $\to$ p pi.

[Response]: We thank you for this helpful suggestion to improve clarity and have implemented both recommendations. In the main text, we now include a precise definition: "A double tag event means that one Λ is reconstructed as $\Lambda \to pe\bar{\nu}_e$ and the antihyperon partner as $\bar{\Lambda} \to \bar{p}\pi^+$. The technique also requires knowledge of the single tag sample which means that only one side is reconstructed as $\bar{\Lambda} \to \bar{p}\pi^+$." Furthermore, as recommended, we have added a new subsection in the Methods that provides a full explanation of the double-tagging technique.

L156: bit unclear about the second uncertainties for the numbers and the BF. The N and eff all have one uncertainty, is this just statistical. The BF has two, is this statistical and systematic – what are the systematic sources here? Can it be reference to methods?

[Response]: Yes, that is correct. The uncertainty quoted for the yields (N) and efficiencies (ϵ) is purely statistical. In contrast, the final branching fraction (BF) is reported with both statistical and systematic uncertainties. We have clarified this distinction in the revised text (lines 160-162) and refer the reader to the Methods section for a detailed discussion of all sources of systematic uncertainty.

L158: it's not completely clear how this is interacting with the signal yield fit. Do you select a signal window in Umiss and analyse these events?

[Response]: Yes. In response to this point, we have added the following clarification in the revised manuscript (lines 164-167): "Whereas the branching fraction determination requires only yields and efficiencies, the extraction of the form factors requires a detailed understanding of the decay kinematics. To increase the sample purity, we retain only events with $|U_{\text{miss}}| < 0.02$ GeV, resulting in 1791 ± 47 signal and 207 ± 30 background events."

L179: is the previous measurement statistically independent?

[Response]: The term "the previous measurement" here refers specifically to the Fermilab result [1] and is therefore statistically independent of our measurement.

L227: "uses the normalization based on ..." → this is a bit too unclear

[Response]: Here, the vector form factor value, $f_1 = \sqrt{3/2}$, under the assumption of exact SU(3) symmetry, is used a normalization. To improve clarity, the sentence has been revised from "one of which uses the normalization based on the SU(3) symmetry ..."

to be

"one uses the SU(3) symmetry-preserved vector form factor value as normalization ..."

Fig 4: Is this pie chart really necessary, we feel it doesn't add any information and pie charts are often discouraged by the data visualisation community.

[Response]: Thank you for this suggestion. Following your recommendation, we have replaced the pie chart with a bar chart (Fig. 4). The bar chart provides a clearer and more quantitative comparison of the individual uncertainty contributions, making it easier to identify which aspects of the measurement could benefit most from further refinement.

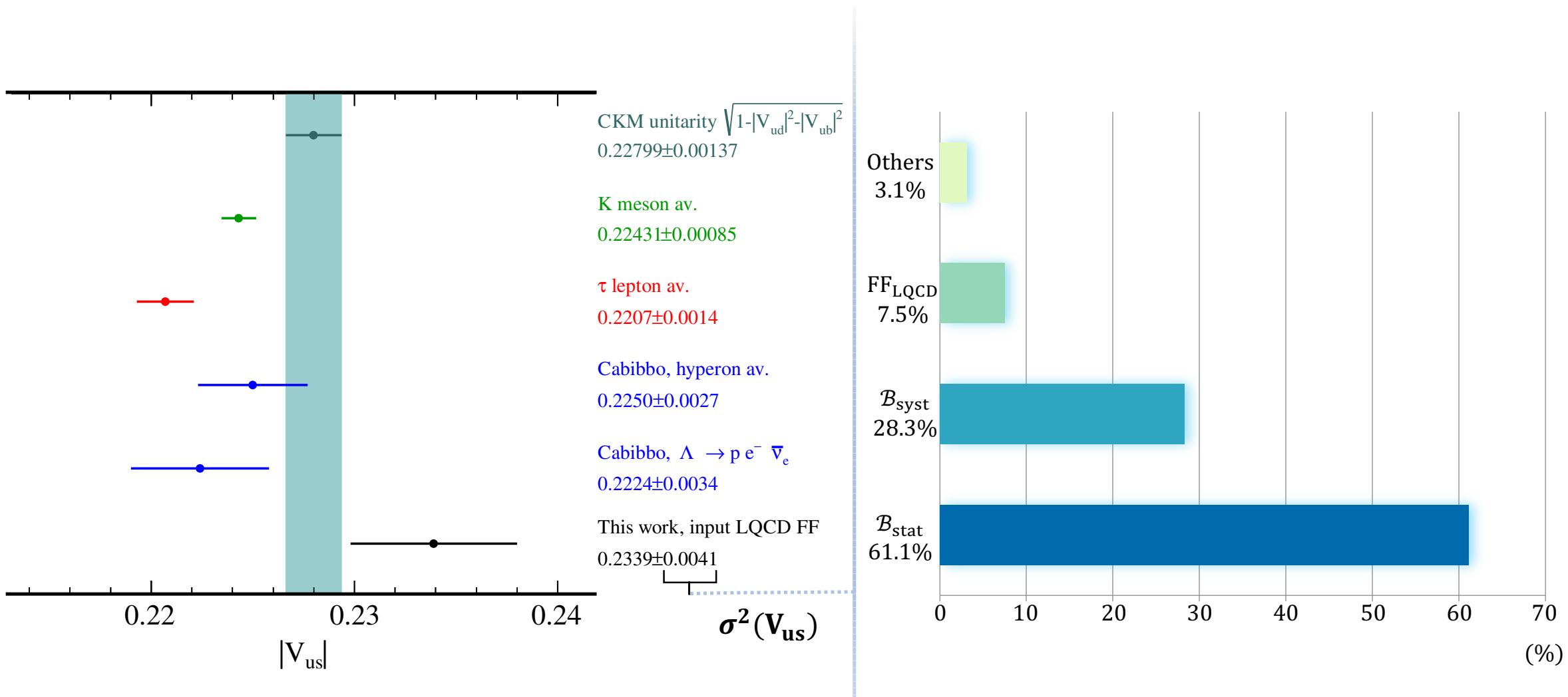

Figure 4: **The $|V_{us}|$ result.** (left) The first-row CKM unitarity constraint and K average are from Ref. [9]. The τ average is from the Heavy Flavor Averaging Group [49]. The two hyperon results were evaluated by Cabibbo in [10,35]. (right) The contributing sources to the $|V_{us}|$ uncertainty, in terms of the variance.

L428: Is this subsection really needed. I don't see where the coordinates are ever used so I'm not sure it's necessary to have to define the coordinate systems.

[Response]: During the BESIII collaboration's internal review, we found that using an inconsistent coordinate system can lead to incorrect results. For this reason, we would like to retain the relevant passage in the Methods section.

L441: "in xi" → shouldn't this be omega?

[Response]: Thank you for pointing this out. It has now been corrected.

L458: "the four data taking period": these are not defined

[Response]: Thank you for this comment. We have clarified the text by specifying the years in parentheses. The revised sentence now reads: "...the four data taking periods (2009, 2012, 2017–2018, and 2018–2019)..."

L508: fitting functions are used for the ST and DT variables. It's not clear what you mean ST and DT variables. Did you change the PDFs in the Umiss fit? If yes, can you be more explicit in the parametrisation change?

[Response]: The ST and DT variables correspond to the beam-constrained mass and $U_{\rm miss}$, respectively. To avoid confusion, the text (lines 566-569) has been updated from

"To test if the fit method produces systematically biased results, alternative fitting functions are used for the ST and DT variables."

to

"The extracted single-tag and double-tag event yields depend on the fitting functions used to model the kinematically constrained invariant mass and $U_{\rm miss}$ spectra. To evaluate the associated systematic uncertainties, we vary the signal and background line shapes in these fits."

We also modified the PDFs in the $U_{\rm miss}$ fit. Following your suggestion, the text (lines 569-574) now explicitly clarifies this change in parametrization:

"For the signal component in the $U_{\rm miss}$ distribution, the Gaussian convolved with the simulated line shape is removed and only the simulated shape is used, resulting in a 0.10% change in the yield. An analogous procedure is applied to the background: first, the linear component is omitted, leaving only the simulated non-leptonic line shape, and as an additional test, the background is modelled with a third-order Chebyshev polynomial, which produces a larger deviation

of 0.79%."

References

[1] J. Dworkin *et al.*, Phys. Rev. D **41**, 780-800 (1990).

[2] M. Ablikim *et al.* (BESIII Collaboration), arXiv:2512.15273 [hep-ex].

[3] G. Barucca *et al.* (PANDA Collaboration), Eur. Phys. J. A **57**, 154 (2021).

[4] M. Achasov *et al.*, Front. Phys. (Beijing) **19**, 14701 (2024).

[5] A. Y. Barnyakov (Super Charm-Tau Factory Collaboration), J. Phys. Conf. Ser. **1561**, 012004 (2020).

[6] N. Cabibbo, E. C. Swallow, and R. Winston, Ann. Rev. Nucl. Part. Sci. **53**, 39-75 (2003).

[7] S. Bacchio and A. Konstantinou, Phys. Rev. Lett. **135**, 231901 (2025).

[8] V. Batozskaya, A. Kupsc, N. Salone, and J. Wiechnik, Phys. Rev. D **108**, 016011 (2023).

[9] A. Garcia, P. Kielanowski, and A. Bohm, Lect. Notes Phys. **222**, 1-173 (1985).

[10] M. Ademollo and R. Gatto, Phys. Rev. Lett. **13**, 264-265 (1964).

[11] S. Weinberg, Phys. Rev. **112**, 1375-1379 (1958).

To the referee #3:

We really appreciate your effort in checking the paper. Please find our responses below. Paragraphs commenced with [Response] are the responses.

– –

Referee #3 (Remarks to the Author):

– –

This manuscript reports a simultaneous experimental measurement of the $\Lambda \to pe^-\bar{\nu}_e$ branching ratio and form factor ratios $g_{a\nu}$, g_w at BESIII. Using the results, the authors determined the magnitude of CKM matrix element $|V_{us}|$. This is an important effort which is definitely worth publication. However, I am not fully convinced that it has reached the standard for publication in *Nature*. My concerns are as follows:

- The result of $\mathcal{B}(\Lambda \to pe^-\bar{\nu}_e)$ reported in this manuscript is not the most precise determination comparing to existing publications, e.g. Ref.[10]. The authors may argue that their determination is direct, whereas Ref.[10] relied on a relative branching fraction, but it still does not change the conclusion.

- Subsequently, the value of $|V_{us}|$ reported in this manuscript is also not the most precise one. Not just that it is 5 times less precise than the average result from kaon decays, it is also less precise than those quoted in Refs.[9,34] which were obtained similarly from hyperon decays.

[Response]: We thank you for the detailed and constructive evaluation of our manuscript. Before starting with detailed answers we would like to address the concerns regarding precision and suitability for *Nature*.

- **Our result is based on the first absolute branching fraction measurement that benefits from the updated α_Λ decay asymmetry parameter determined by BESIII [1, 2]. In comparison, all previous measurements of this channel adopted an outdated value of α_Λ, which may have introduced unknown biases.**

  As noted by you, our work reports the first absolute branching fraction measurement for this decay. More importantly, it is also the first such measurement to incorporate the re-evaluated α_Λ decay asymmetry parameter from BESIII [1, 2]. All earlier determinations, including those in Refs. [3] (which used $\Lambda \to p\pi^-$ as a normalization channel) and [4]

(where the Λ originated from $\Xi^- \to \Lambda\pi^-$), relied on the previous, now-superseded value of α_Λ. While we cannot quantify the exact impact of this change on those results, it raises a legitimate question regarding the validity of earlier measurements. This further underscores the need for a new, direct determination such as ours.

- **The present precision of the branching fraction and $|V_{us}|$ results is not the central highlight of this study. Instead, our core advance lies in a novel method that enables the first exploitation of polarization and quantum entanglement in baryon semileptonic decays.**

  Our method itself is unequivocally novel, as it marks the first exploitation of polarization and quantum entanglement in baryon semileptonic decays. The revised manuscript has now highlighted this explicitly. The advantage of the approach is quantified through a direct comparison with the Fermilab measurement [5]: although their dataset is approximately twenty times larger than ours, we achieve a comparable precision for g_w, while the uncertainty on g_{av} is only about a factor of 2.5 larger, and, most notably, we determine g_{av2} for the first time, a parameter previously inaccessible. This demonstrates a substantially enhanced single-event sensitivity. This study thus represents a foundational demonstration of the method.

- **The novelty of our work is further evidenced by two "firsts" that explicitly advance beyond previous measurements.**

  We performed the first measurement of charge-specific form factors (consistent with charge symmetry) and the first determination of g_{av2} (consistent with SU(3) symmetry). Both of these "firsts" explicitly demonstrate that our analysis advances beyond previous measurements, even though the absolute precision of the branching fraction and $|V_{us}|$ is not yet the world's best.

- **This work has established a new experimental paradigm that enables future high-precision studies of baryon semi-leptonic decays.**

  Along with the first exploitation of polarization and quantum entanglement in baryon semileptonic decays, this work establishes the foundation for a systematic research program. The same method is already being extended to our following studies of other baryon semileptonic decays at BESIII [6] and is applicable to both present and future particle-antiparticle collider facilities such as the upcoming PANDA experiment [7] and the proposed Super Tau-Charm Factories [8,9]. Because form factors are interrelated in different semileptonic decays [10], results obtained with this method can be combined across

channels. Together with continued advances in lattice QCD [11], this program offers a clear path toward a model-independent determination of $|V_{us}|$ with precision competitive with that from kaon decays, thereby providing a critical and independent test of CKM unitarity. We therefore contend that the novelty and impact of this work lie in pioneering an efficient new experimental paradigm that has the potential to redefine precision studies of baryon semi-leptonic decays.

We have revised the corresponding discussion in the manuscript to emphasize these points. The updated text (shown in red) reads:

"The novel modular approach [12] is applied in the analysis marking the first exploitation of polarization and quantum entanglement in baryon semi-leptonic decays. This innovative methodology significantly enhances the single-event sensitivity, as demonstrated by a direct comparison with the Fermilab measurement [5]: although their dataset is approximately twenty times larger than ours, we achieve a comparable precision for g_w, while the uncertainty on g_{av} is only about a factor of 2.5 larger, and, most notably, we determine g_{av2} for the first time, a parameter previously inaccessible. Along with the first exploitation of polarization and quantum entanglement in baryon semileptonic decays, this work establishes the foundation for a systematic research program. The same method is already being extended to our following studies of other baryon semileptonic decays at BESIII [6] and is applicable to both present and future particle-antiparticle collider facilities such as the upcoming PANDA experiment [7] and the proposed Super Tau-Charm Factories [8, 9]. Because form factors in different semileptonic decays are interrelated [10], results obtained with this method can be combined across channels. Together with continued advances in lattice QCD [11], this program offers a clear path toward a model-independent determination of $|V_{us}|$ with precision competitive with that from kaon decays, thereby providing a critical and independent test of CKM unitarity."

The reliability of the quoted theory uncertainty is also a concern; in particular, I argue that the study of radiative corrections in this manuscript is incomplete. The authors utilized the results in Ref.[47], but that paper only studied the so-called "model-independent" part of the radiative correction. They did not study the "model-dependent" piece which, according to the paper, only renormalizes the form factors. However, this piece is crucial if one tries to connect the measured branching ratio to the Standard Model (which is also a "model" according to the criteria of Ref.[47]) in order to extract $|V_{us}|$. For example, the universal electroweak logarithm (which is common to all semileptonic decays of hadron, see, e.g. Phys.Rev.Lett.71(1993)3629-3632)

$S_{\mathrm{EW}} = 1 + \frac{2\alpha}{\pi}\left(1 - \frac{\alpha_s}{4\pi}\right) \ln \frac{M_Z}{M_\rho} + \mathcal{O}\left(\frac{\alpha\alpha_s}{\pi^2}\right) \approx 1.0232$

corrects f_1^2 from its pure-QCD value by 2%, which is at same level with the experimental uncertainty. Such corrections must be applied. Then, there is the non-perturbative part of the radiative correction to f_1 which must be accounted for in the error budget. This is a standard practice, for example, in the determination of $|V_{ud}|$ from nuclear, neutron or pion decay, as well as the determination of $|V_{us}|$ from kaon decays which, I believe, should also be followed by the authors of this manuscript.

[Response]: You are correct. The radiative correction employed in our analysis corresponds to the model-independent part of the radiative corrections in Ref. [13].

Regarding electroweak radiative corrections for β decay, we adopt the following correction in order to remain consistent with the assumptions underlying the SU(3) and lattice QCD-based approaches.

For the determination of $|V_{us}|_{\mathrm{SU(3)}}$, we apply the revised universal electroweak correction formula [14, 15], consistent with the treatment of the model-independent radiative correction in Ref. [13]:

$$\Delta_R^V = \frac{\alpha}{2\pi}\left[3 \ln\left(\frac{M_Z}{M_p}\right) + \ln\left(\frac{M_Z}{M_A}\right) + 2C_{\mathrm{Born}} + A_g\right] = 0.0227,$$

where $M_Z = 91.188$ GeV/c^2, $M_p = 0.938$ GeV/c^2, $M_A = 1.2$ GeV/c^2 (corresponding to the axial-vector meson $a_1(1260)$), $C_{\mathrm{Born}} \simeq 0.891$, and $A_g \simeq -0.34$. This correction is applied to the Fermi coupling constant as:

$$G_F^2 \equiv G_F^2(1 + \Delta_R^V).$$

For the determination of $|V_{us}|_{\mathrm{LQCD}}$, we follow the prescription

$$G_F \equiv G_F(1 + C) = G_F(1 + 0.0105)$$

as calculated by Garcia and Kielanowski [16] and adopted in recent lattice QCD studies [11].

With respect to the uncertainty associated with electroweak radiative corrections, previous studies indicate that the relative uncertainty of the correction factor is very small, at most 1.6% [14]. Moreover, the referenced lattice QCD calculation does not include an uncertainty associated with this correction [11]. Given the current precision of our measurement, we therefore do not assign an additional systematic uncertainty to this correction.

Concerning model-dependent corrections to the vector form factor f_1, no additional correction is required in either approach. For $|V_{us}|_{\mathrm{SU(3)}}$, the normalization of f_1 is inherently based on SU(3) symmetry assumptions. For $|V_{us}|_{\mathrm{LQCD}}$, the value of f_1 is obtained directly from first-

principle lattice QCD calculations, which likewise do not require a separate model-dependent correction.

After incorporating these updated treatments of radiative corrections, our results are revised to

$$|V_{us}|_{\mathrm{SU(3)}} = 0.2190 \pm 0.0094 \qquad and \qquad |V_{us}|_{\mathrm{LQCD}} = 0.2339 \pm 0.0041.$$

The corresponding discussion has been added to the revised manuscript (lines 535-540). Detailed derivations of $|V_{us}|_{\mathrm{SU(3)}}$ and $|V_{us}|_{\mathrm{LQCD}}$ are provided in Appendix 1 and Appendix 2, respectively.

I also had a hard time reproducing the numerical results in Table I, $|V_{us}|_{\mathrm{SU(3)}} = 0.2199(94)$. This result is said to be obtained from Eq.(1):

$\mathcal{B} = \frac{\tau_\Lambda}{\hbar} G_F^2 |V_{us}|^2 \frac{\beta^5 M_\Lambda^2}{60\pi^3} f_1^2 \left[\left(1 - \frac{3}{2}\beta\right)\left(1 + 3g_{av}^2\right) + \frac{2}{7}\beta^2 \left(3 + 3g_w + 2g_w^2 + 6g_{av}^2\right)\right]$

where $\beta \equiv (M_\Lambda - M_p)/M_\Lambda$, and I have set $g_2 = 0$ following the treatment in the manuscript. The input I used are (here I only quote the central values, and notice that the error bars of all inputs below are almost symmetric):

$M_\Lambda = 1115.683\ \mathrm{MeV}$

$M_p = 938.27208816\ \mathrm{MeV}$

$G_F = 1.1663788 \times 10^{-11}\ \mathrm{MeV}^{-2}$

$\tau_\Lambda = 2.617 \times 10^{-10}\ \mathrm{s}$

together with the experimental results from this paper (obtained with the assumption $g_2 = 0$):

$\mathcal{B}_c = 8.16 \times 10^{-4}$

$g_{av} = 0.729$

$g_w = 0.89$

as well as $f_1 = -\sqrt{3/2}$ in the SU(3) limit. Here, $\mathcal{B}_c$ is the actual measured branching ratio which is corrected from the one in Eq.(1) by the "model-independent" part of the radiative correction, given in Eq.(10):

$\mathcal{B}_c = \mathcal{B} \cdot (1 + Cr)$

with Cr=0.016. With all these inputs, I obtain the central value $|V_{us}|_{\mathrm{SU(3)}} = 0.2260$ which is different from 0.2199 quoted in the paper.

[Response]: The discrepancy between our calculated results and yours arises from our inclusion of the q^2 dependence of the form factors, which was not taken into account in your evaluation. To avoid any possible misinterpretation, we have revised the description of the $|V_{us}|$ determination accordingly.

In addition, we identified that the originally implemented q^2 dependence was not fully consistent with the description provided in the text. This inconsistency has now been corrected, and the manuscript has been updated accordingly (lines 519-529).

We have further performed an independent cross-check among the authors and verified that consistent results are obtained by all using the revised framework. Details of the calculation of $|V_{us}|_{\mathrm{SU(3)}}$ are provided in Appendix 1.

It is also unclear which lattice inputs from Ref.[27] were used to obtain the second number, $|V_{us}|_{\mathrm{LQCD}} = 0.2332(42)$. Is it just the two q^2-dependence parameters $\langle r^2_{f_1}\rangle$ and $\langle r^2_{g_1}\rangle$, or also include the $q^2 = 0$ couplings f_1, f_2, g_1 and g_2? Also, notice that Ref.[27] is not yet published, so the reliability of its results is in question.

[Response]: Ref. [27] has now been published in *Physical Review Letters* [11], and the reference has been updated accordingly in the revised manuscript. We note that the published PRL version reports slightly different final values of the form factors compared with the earlier arXiv preprint that was used in our original $|V_{us}|$ calculation. We have therefore updated our results to align with the values quoted in the published article.

The determination of $|V_{us}|_{\mathrm{LQCD}}$ incorporates the lattice QCD form factors f_1, f_2, g_1, and g_2, including their full q^2 dependence, as reported in Ref. [11]. The value of $|V_{us}|_{\mathrm{LQCD}}$ is extracted using Eq. (A20) of that reference. For consistency with the lattice QCD studies, the electroweak radiative correction is implemented via $G_F \equiv G_F(1 + C) \equiv G_F(1 + 0.0105)$. The corresponding description has been added to the revised manuscript (lines 533-540). Further details of the $|V_{us}|_{\mathrm{LQCD}}$ calculation are provided in Appendix 2.

I am also unable to confirm the consistency of the third result, $|V_{us}|\sqrt{f_1^2 + 3g_1^2} = 0.4543(76)$ with the first two. In principle, if I plug in $f_1 = -\sqrt{3/2}$ and $g_{av} = g_1/f_1 = 0.729$, I should get back $|V_{us}|_{\mathrm{SU(3)}} = 0.2199$; but I get 0.2303 instead. Clarifications to all these apparent discrepancies would be useful.

[Response]: When calculating $|V_{us}| \cdot \sqrt{f_1^2 + 3g_1^2}$, we start from Eq.(1) and adopt two well-motivated simplifications. First, we set $g_2 = 0$, as expected in the SU(3)-symmetry limit. Second, we neglect the contribution of the $\mathcal{F}_2$ term, since it is multiplied by the small factor $\frac{2}{7}\beta^2 \approx 0.007$. With these simplifications, the resulting expression coincides with the standard next-to-leading order form of the semi-leptonic decay rate used in lattice QCD calcultions as Eq. (A19) [11]. Consequently, we apply the same corrections to both the decay width and the weak decay constant as those adopted in the determination of $|V_{us}|_{\mathrm{LQCD}}$.

To clarify this point, we have added the following sentences to the relevant section of the

manuscript (lines 230-239):

"The product of $|V_{us}|$ with the dominant form factor contribution at zero momentum transfer, $q^2=0$, is extracted as $|V_{us}|\cdot\sqrt{f_1^2+3g_1^2} = 0.4496 \pm 0.0061_{\text{BESIII BF}_{\text{stat}}} \pm 0.0041_{\text{BESIII BF}_{\text{syst}}} \pm 0.0009_{\tau_\Lambda} \pm 0.0011_{\text{RC}}$. The reason is that after assuming $g_2=0$ and neglecting the $\mathcal{F}_2$ contribution, as the kinematic pre-factor is small, $\frac{2}{7}\beta^2 \approx 0.007$, the partial decay width Γ is approximately proportional to $V_{us}^2(f_1^2+3g_1^2)$. Under these two assumptions, the short-handed formula becomes identical to the the standard next-to-leading order form in the LQCD framework [11]. As a technical side note, the radiative corrections applied to the weak decay constant differ between $|V_{us}|_{\text{SU(3)}}$ and $|V_{us}|_{\text{LQCD}}$. Consequently, when applying radiative corrections to the $|V_{us}|\cdot\sqrt{f_1^2+3g_1^2}$, we adopt the same correction method as used for $|V_{us}|_{\text{LQCD}}$."

Details of the calculation of $|V_{us}|\cdot\sqrt{f_1^2+3g_1^2}$ are provided in Appendix 3.

References

[1] M. Ablikim *et al.* (BESIII Collaboration), Nature Phys. **15**, 631-634 (2019).

[2] M. Ablikim *et al.* (BESIII Collaboration), Phys. Rev. Lett. **129**, 131801 (2022).

[3] J. Wise *et al.*, Phys. Lett. B **91**, 165-168 (1980).

[4] M. Bourquin *et al.* (Bristol-Geneva-Heidelberg-Orsay-Rutherford-Strasbourg Collaboration), Z. Phys. C **21**, 1 (1983).

[5] J. Dworkin *et al.*, Phys. Rev. D **41**, 780-800 (1990).

[6] M. Ablikim *et al.* (BESIII Collaboration), arXiv:2512.15273 [hep-ex].

[7] G. Barucca *et al.* (PANDA Collaboration), Eur. Phys. J. A **57**, 154 (2021).

[8] M. Achasov *et al.*, Front. Phys. (Beijing) **19**, 14701 (2024).

[9] A. Y. Barnyakov (Super Charm-Tau Factory Collaboration), J. Phys. Conf. Ser. **1561**, 012004 (2020).

[10] N. Cabibbo, E. C. Swallow, and R. Winston, Ann. Rev. Nucl. Part. Sci. **53**, 39-75 (2003).

[11] S. Bacchio and A. Konstantinou, Phys. Rev. Lett. **135**, 231901 (2025).

[12] V. Batozskaya, A. Kupsc, N. Salone, and J. Wiechnik, Phys. Rev. D **108**, 016011 (2023).

[13] R. Flores-Mendieta, A. Garcia, A. Martinez, and J. J. Torres, Phys. Rev. D **55**, 5702-5718 (1997).

[14] A. Czarnecki, W. J. Marciano, and A. Sirlin, Phys. Rev. D **100**, 073008 (2019).

[15] A. Czarnecki, W. J. Marciano, and A. Sirlin, Phys. Rev. D **70**, 093006 (2004).

[16] A. Garcia, P. Kielanowski, and A. Bohm, Lect. Notes Phys. **222**, 1-173 (1985).

Appendix 1

222

```
In[1]:= (*Calculation of the mean value of |Vus|_{SU(3)}*)
(*Quit *)

gav = 0.729;
gw = 0.89;
f10 = Sqrt[3/2];
f20 = gw * f10;
g10 = gav * f10;

BF0 = 8.16 * 10^-4;
MIDPRC = 0.016;(*Model-independent radiative correction factor*)
BF = BF0/(1 + MIDPRC);(*Model-independent radiative correction*)

tau = 2.617 * 10^-10;(*s*)
hbar = 6.582119569 * 10^-22;(*MeV s*)
GamLambda = 1/tau * hbar;(*MeV*)
Gamsemi = GamLambda * BF;(*MeV*)
mLambda = 1115.683;(*MeV*)
mProton = 938.27208816;(*MeV*)
mElectron = 0.51099895;

Delta = mLambda - mProton;(*MeV*)
delta = (mLambda - mProton)/mLambda;
pi = 3.141592654;

GF0 = 1.1663788 * 10^-11;(*MeV^-2*)
MDPRC = 0.0227;(*Model-dependent ratiative correction factor*)
GF = GF0 * Sqrt[1 + MDPRC];(*Model-dependent ratiative correction*)

mV = 892;(*MeV*)
mA = 1273;(*MeV*)
aR = 0.9 * 10^-6;(*MeV^-2*)
q2min = mElectron^2;
q2max = (mLambda - mProton)^2;

f1[q2_] := f10 * (1 + q2 * (1/mV^2 + 1/(mV^2 + aR^-1)));
f2[q2_] := f20 * (1 + q2 * (1/mV^2 + 1/(mV^2 + aR^-1) + 1/(mV^2 + 2 * aR^-1)));
g1[q2_] := g10 * (1 + q2 * (1/mA^2 + 1/(mA^2 + aR^-1)));
```

223

```
f1c = Integrate[f1[q2], {q2, q2min, q2max}] / (q2max - q2min);
f2c = Integrate[f2[q2], {q2, q2min, q2max}] / (q2max - q2min);
g1c = Integrate[g1[q2], {q2, q2min, q2max}] / (q2max - q2min);

Vus =
  Sqrt[(Gamsemi * (60 * pi^3) / (GF^2 * Delta^5 * ((1 - 3/2 * delta + 6/7 * delta^2) * f1c^2 + 4/7 * delta^2 * f2
      + (3 - 9/2 * delta + 12/7 * delta^2) * g1c^2 + 6/7 * delta^2 * f1c * f2c)))]
```

Out[33]=

```
0.219011
```

Appendix 2

224

```
(*Calculation of the mean value of |Vus|_{LQCD}*)
tau = 2.617 * 10^-10;
hbar = 6.582119569 * 10^-22; (*MeV s*)
mLambda = 1115.683; (*MeV*)
mProton = 938.27208816; (*MeV*)
mElectron = 0.51099895;
delta = (mLambda - mProton)/mLambda;
pi = 3.141592654;

GF0 = 1.1663788 * 10^-11; (*MeV^-2*)
MDPRC = 0.0105; (*Model-dependent ratiative correction factor*)
GF = GF0 * (1 + MDPRC); (*Model-dependent ratiative correction*)

BF0 = 8.16 * 10^-4;
MIDPRC = 0.016; (*Model-independent radiative correction factor*)
BF = BF0/(1 + MIDPRC); (*Model-independent radiative correction*)

GamLambda = 1/tau * hbar; (*MeV*)
Gamsemi = GamLambda * BF; (*MeV*)

(*All of the following are from the LQCD paper,
Phys.Rev.Lett.135,231901 (2025).*)
(*These values are cited from Table 1 of the LQCD paper*)

f1 = 1.1849;
f2 = 0.821;
g1 = 0.8178;
g2 = -0.082;
radiiF1 = 9.50;
radiiG1 = 6.46;
(*The following equations are Eq.(A19) and Eq.(A20) of the LQCD paper*)
partNLO = 1/(60 * pi^3) * (1 - 3/2 * delta) * (f1^2 + 3 * g1^2);
partNNLO = delta^2/(70 * pi^3) *
   (f1 * f2 + 2/3 * f2^2 - 14/3 * (g1 * g2)/delta + f1^2 (1 + 1/9 * radiiF1) + g1^2 (2 + 5/9 * radiiG1));
Vus2 = Gamsemi / (GF^2 * mLambda^5 * delta^5 * (partNLO + partNNLO));
Vus = Sqrt[Vus2]

Out[25]=
0.23388
```

Appendix 3

225

```
(*Calculation of the mean value of |Vus|*Sqrt[f1^2+3*g1^2]*)
tau = 2.617 * 10^-10;
hbar = 6.582119569 * 10^-22; (*MeV s*)
GamLambda = 1/tau * hbar; (*MeV*)
Gamsemi = GamLambda * BF; (*MeV*)

delta = (mLambda - mProton)/mLambda;
pi = 3.141592654;

GF = 1.1663788 * 10^-11; (*MeV^-2*)
mLambda = 1115.683; (*MeV*)
mProton = 938.27208816; (*MeV*)
mElectron = 0.51099895;

GF0 = 1.1663788 * 10^-11; (*MeV^-2*)
MDPRC = 0.0105; (*Model-dependent ratiative correction factor*)
GF = GF0 * (1 + MDPRC); (*Model-dependent ratiative correction*)

BF0 = 8.16 * 10^-4;
MIDPRC = 0.016; (*Model-independent radiative correction factor*)
BF = BF0/(1 + MIDPRC); (*Model-independent radiative correction*)

(*The following equation is Eq.(A19) of the LQCD paper,
Phys.Rev.Lett.135,231901 (2025).*)

partNLO = 1/(60 * pi^3) * (1 - 3/2 * delta);

VusProd2 = Gamsemi / (GF^2 * mLambda^5 * delta^5 * partNLO);

VusProd = Sqrt[VusProd2]
```

Out[19]=

```
0.449598
```

To the referee #1:

We really appreciate your effort in checking the paper. Please find our responses below. Paragraphs commenced with [authors] are the responses.

– –

Referee #1 (Remarks to the Author):

– –

The revised manuscript addresses my previous remarks and observations. The authors have significantly improved the clarity of the manuscript by reworking paragraphs, adding supplementary material and providing a convincing justification for publishing in Nature. I am fully satisfied by the feedback provided by the authors and recommend publication.

[authors]: Thank you for the recommendation to publish the paper. We have addressed your comments and suggestions as follows.

My only comment at this point regards the new title: "Determining baryon semi-leptonic decays...", which I find a bit awkward. I would replace "Determining" by "Investigating" or "An investigation of".

[authors]: Following your advice and taking into account the editor's input, we have revised the title to: "Exploring baryon semi-leptonic decays with polarization and entanglement".

A couple of other minor editorial comments:

- lines 78-79: "which was performed by" → "by"

- line 90: "...the comparable level" → "...a comparable level"

[authors]: Both of these textual modifications have been made according to your advice.

To the referee #2:

We really appreciate your effort in checking the paper. Please find our responses below. Paragraphs commenced with [authors] are the responses.

– –

Referee #2 (Remarks to the Author):

– –

Many thanks to the authors for their improvements and edits in the revised version. Your response helps to clarify the novel aspects. The added paragraph towards the ends helps to underline these and the additions to the Abstract also make it clearer from the start. There are still many editorial changes I would suggest to improve the readability and language. The second version is better but I'm not sure it is a considerable improvement. Some of these I give further guidance on below but I believe should predominantly lie under the remit of the Nature editorial team.

We are confident the scientific procedures are robust, it is just the description in various places that could do with improvement.

[authors]: Thank you for your feedback. In addition, following your further suggestions, we have made further revisions to the manuscript, as detailed below.

Follow ups:

Data and methodology. The response contained some explanation of how data/MC corrections (particularly with regard to PID) are accounted for using weights. We didn't find too much change to the text. It would be useful to provide some more details on MC corrections in the methods part.

[authors]: Thank you for your comment. In fact, our previous response was only a summary of our data/MC corrections. The detailed description was already included in the original version of the manuscript submitted to the journal, so we did not make extensive changes. Specifically, for the measurement of form factor ratios, the corrections for the detection efficiencies of final-state particles (including tracking and PID) are described in lines 502–507. For the branching fraction measurement, the details of the MC corrections are provided in the subsections "1. Requirement on the number of tracks and Λ vertex fit", "2. p and e^- detection", and "3. Kinematic fit" under the section "Systematic uncertainties of branching fraction determination".

Regarding the MC corrections for PID that you specifically pointed out, to help readers further understand the details, we have cited a dedicated study on electron tracking and PID efficiency corrections at BESIII [1] in the updated manuscript, and we explicitly state that we used the same method to obtain our correction factors for tracking and PID.

Title still sounds a little odd to me. Is it really determining the decays? I would say it is "Determination of the decay properties".

[authors]: Following your advice and taking into account the editor's input, we have revised the title to: "Exploring baryon semi-leptonic decays with polarization and entanglement".

Abstract: Still needs some editorial fine tuning. The meaning is sometime ambiguous. For example I think in the first line you mean "the structure has been probed" and then "In order to access the static properties these determinations must be propagated to zero ..."

[authors]: We appreciate your careful reading and your constructive comment on the abstract. Accordingly, under the editor's guidance and to adhere to Nature's formatting and content requirements, we have thoroughly rewritten the abstract. The revised abstract is presented below:

"The unitarity of the Cabibbo–Kobayashi–Maskawa (CKM) matrix is a cornerstone of the Standard Model (SM). Precise tests of this unitarity require independent determinations of its elements, such as $|V_{us}|$, which governs the transition between strange and up quarks. Current measurements from kaon and tau decays show tensions that may hint at physics beyond the SM [2–4]. Hyperon semi-leptonic decays provide alternative probes, but have remained largely untapped because previous experiments lacked sufficient kinematic information, rendering the measurements insensitive to the relevant form factors [5]. Here we report measurements of the axial-vector and weak-magnetism couplings, as well as the first determinations of the absolute branching fraction and weak-electricity coupling in $\Lambda \to pe^-\bar{\nu}_e$, achieved by exploiting the polarization and quantum entanglement of $\Lambda\bar{\Lambda}$ pairs produced at the J/ψ resonance. Combining our results with recent lattice Quantum Chromodynamics calculations [6] gives $|V_{us}|_{\rm LQCD} = 0.2339 \pm 0.0041$, a model-independent determination consistent with CKM unitarity. By pioneering the exploitation of polarization and quantum entanglement in baryon semi-leptonic decays, our method enhances single-event sensitivity and it is broadly applicable to other baryon semi-leptonic decays, establishing the foundation for a systematic research program that can achieve precision comparable to that of kaon decays and provide a stringent

independent test of the SM."

L51: is the quantity under the symmetry precise (is it worth mentioning that the value if -sqrt(3)/2?)

[authors]: Under $SU(3)$ symmetry, the value of f_1 is precisely $-\sqrt{3/2}$. However, since this part is intended only as a methodological overview and introduction, we prefer not to explicitly quote the value of f_1 here. Similarly, we survey multiple alternative models that yield predictions for f_1, and we likewise refrain from listing their concrete numerical values. Later in the manuscript, when this input is actually needed, we clearly provide the value $-\sqrt{3/2}$ (see lines 207–208: "Under this assumption, f_1 takes on the value $-\sqrt{3/2}$").

L73 - L75: does the "new experimental value" mean the value determined in this work? This doesn't seem to target g_1 so not sure how it is an important cross validation?

[authors]: Thank you for the question. Yes, the "new experimental value" refers to the value determined in this work. You are also correct that we do not directly target g_1. To avoid any misunderstanding, we have removed this sentence from the updated manuscript.

L75: Is the term mirror-asymmetric well defined? Does this mean G-parity asymmetric or purely parity violating?

[authors]: Thank you for raising this important point. The term "mirror-asymmetric" was used to describe G-parity asymmetry. To ensure the meaning is unambiguous to all readers, we have revised the manuscript by replacing "mirror-asymmetric" with "G-parity asymmetric".

Eq3: Would be tempted to change the comma to a semi-colon to give a distinction in the pdf between the observables and parameters. I.e. W(ξ; ω)

[authors]: You are correct. We have made the change as suggested: Eq.(3) now reads $\mathcal{W}(\xi;\omega)$ in the updated manuscript.

Fig 2. Much improved. Would prefer more spacing and less shadow over symbols.

[authors]: Thank you for your feedback on Fig. 2. We have modified the figure according to your suggestions. The updated version is shown below.

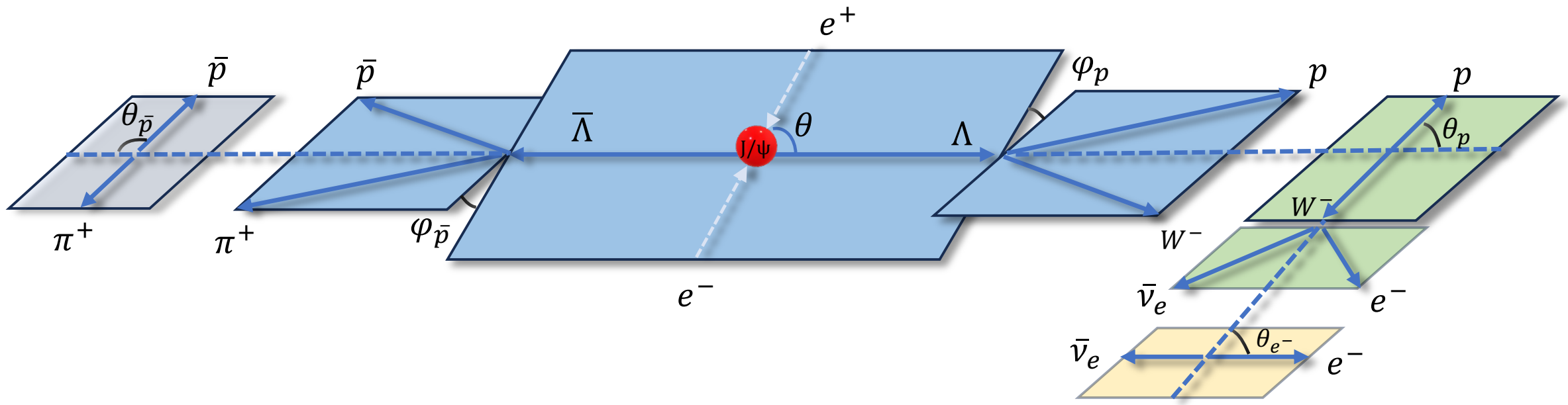

Figure 1: **Definition of the helicity angles for $e^+e^- \to J/\psi \to \Lambda(\to pe^-\bar{\nu}_e)\bar{\Lambda}(\to \bar{p}\pi^+)$.** The angles θ, $\theta_{\bar{p}}$, $\varphi_{\bar{p}}$, θ_p, φ_p, and θ_e are the helicity angles of the Λ, $\bar{p}$, p, and lepton in the e^+e^- c.m. system (blue plane), $\bar{\Lambda}$ rest frame (gray plane), Λ rest frame (green plane), and W^- rest frame (yellow plane), respectively.

L251: Grammar here. "Determination" twice in quick succession.

[authors]: Thank you for pointing out the grammatical issue. In the updated manuscript, we have removed the second occurrence of "Determination".

L252: What does the "modular" approach really refer to (this was a bit unclear to me - what is modular about it?)

[authors]: The "modular" approach (Ref. [39]) means the decay chain is broken into independent modules: production, semileptonic decay, and tagging decay. Each module is represented as a matrix. The total angular distribution is obtained by combining these matrices, which allows easy adaptation to other decay modes.

L440: PID is defined as acronym but never used again

[authors]: Thanks for noting this. We have deleted the acronym definition for "PID" in the revised version, as it was not used again.

L442: Which is the "other track" in this case?

[authors]: Thank you for the question. In our event selection, we require exactly four charged tracks. Apart from the two tracks on the tag side, there are two tracks on the double-tag (DT) side. For these two DT tracks, we require one of them to satisfy the electron/positron PID criteria. The remaining track is referred to as "the other track". To avoid ambiguity, we have revised the wording in the updated manuscript, changing "The other track" to "The

charged track on the DT side other than the electron/positron".

L453: GeV/c2 → GeV/c

[authors]: Thanks for pointing this out. We have made the change from "GeV/c^2" to "GeV/c" in the revised version.

References

[1] Chai, X., Wang, M., Ji, X. *et al.*, Radiat Detect Technol Methods (2025).

[2] C. Y. Seng, D. Galviz, W. J. Marciano, and U. G. Meißner, Phys. Rev. D **105**, 013005 (2022).

[3] P. Boyle *et al.* (RBC and UKQCD Collaborations), Phys. Rev. Lett. **121**, 202003 (2018).

[4] R. J. Hudspith, R. Lewis, K. Maltman, and J. Zanotti, Phys. Lett. B **781**, 206-212 (2018).

[5] S. Navas *et al.* (Particle Data Group), Phys. Rev. D **110**, 030001 (2024).

[6] S. Bacchio and A. Konstantinou, Phys. Rev. Lett. **135**, 231901 (2025).

To the referee #3:

We really appreciate your effort in checking the paper. Please find our responses below. Paragraphs commenced with [authors] are the responses.

– –

Referee #3 (Remarks to the Author):

– –

The authors have adequately responded to the questions in my first report, and the manuscript is substantially improved. At this stage, I am happy to recommend its publication in Nature. Nonetheless, I invite the authors to consider the following optional suggestions:

[authors]: Thank you for the recommendation to publish the paper. We have followed up on your comments and suggestions as follows.

1. The authors adopted the model-dependent radiative correction Δ_R^V from Ref.[67]. That work, however, was explicitly focused on neutron beta decay. The Born term in the expression, for instance, involves the nucleon form factors (axial and isosinglet magnetic Sachs form factor, to be more precise). The appropriateness of applying this value to hyperon decay should be clarified. As a minimal improvement, the authors could at least replace the Born term with the corresponding hyperon contribution.

2. Even for Δ_R^V alone, several new studies have appeared in recent years, so citing only Ref. [67] (which is based on a hadronic model evaluation) is incomplete. Other relevant evaluations include:

Dispersion relation: Phys.Rev.Lett. 121 (2018) 24, 241804, Phys.Rev.D 104 (2021) 3, 033003

Lattice QCD: Phys.Rev.D 101 (2020) 11, 111301, Phys.Rev.Lett. 132 (2024) 19, 191901

Hadron model: Phys.Rev.D 103 (2021) 11, 113001

I invite the authors to take these evaluations into account when quoting their final result.

[authors]: Thank you for raising these important points.

The model-independent radiative correction Δ_R^V from Ref. [67] was indeed derived specifically for neutron beta decay, as you noted. Now we have replaced the Born term with the Λ hyperon contribution, and the calculated value is [1–3]:

$$C = 3g_A(0.266)(\mu_p + \mu_n) = -0.6286, \quad (1)$$

compared to $C_{\rm Born} = 0.891$ [4]. Here, $\mu_p + \mu_n = 0.8798$ is the sum of the proton and neutron magnetic moments [5], and $g_A = -0.8953$ is the axial-vector form factor under the SU(3) assumption [6]. This gives $\Delta_R^V = 0.0191$ for the Λ form factors, compared to $\Delta_R^V = 0.0227$ for the nucleon form factors.

After incorporating the updated radiative correction, our result is revised to $|V_{us}|_{\rm SU(3)} = 0.2194 \pm 0.0094$. The corresponding discussion has been updated in the revised manuscript (Lines. 546-551) as follows:

"Although the universal, nuclear-structure-independent electroweak radiative correction has been extensively studied in Refs. [1–3, 7–11], such studies for hyperon decays are very limited. The electroweak radiative correction is incorporated through the prescription $G_F^2 \equiv G_F^2 \cdot (1 + \Delta_R^V) = G_F^2 \cdot (1 + 0.0191)$, following Refs. [1–3], replacing the nucleon contribution to Δ_R^V with that of the Λ hyperon."

We thank the referee for the list of recent evaluations of Δ_R^V. Although those works are not directly applicable to hyperon decays, we have added a corresponding discussion and included them as references in the updated manuscript for completeness.

References

[1] W. J. Marciano and A. Sirlin, Phys. Rev. Lett. **56**, 22 (1986).

[2] W. J. Marciano and A. Sirlin, Phys. Rev. Lett. **96**, 032002 (2006).

[3] A. Czarnecki, W. J. Marciano and A. Sirlin, Phys. Rev. D **100**, 073008 (2019).

[4] A. Czarnecki, W. J. Marciano and A. Sirlin, Phys. Rev. D **70**, 093006 (2004).

[5] S. Navas *et al.* (Particle Data Group), Phys. Rev. D **110**, 030001 (2024).

[6] N. Cabibbo, E. C. Swallow and R. Winston, Ann. Rev. Nucl. Part. Sci. **53**, 39-75 (2003).

[7] C. Y. Seng, M. Gorchtein, H. H. Patel, and M. J. Ramsey-Musolf, Phys. Rev. Lett. **121**, 241804 (2018).

[8] K. Shiells, P. G. Blunden, and W. Melnitchouk, Phys. Rev. D **104**, 033003 (2021).

[9] C. Y. Seng, X. Feng, M. Gorchtein, and L. C. Jin, Phys. Rev. D **101**, 111301 (2020).

[10] P. X. Ma, X. Feng, M. Gorchtein, L. C. Jin, K. F. Liu, C. Y. Seng, B. G. Wang, and Z. L. Zhang, Phys. Rev. Lett. **132**, 191901 (2024).

[11] L. Hayen, Phys. Rev. D **103**, 113001 (2021).
